# Assessing the Role of a Malonamide Linker in the Design of Potent Dual Inhibitors of Factor Xa and Cholinesterases

**DOI:** 10.3390/molecules27134269

**Published:** 2022-07-02

**Authors:** Rosa Purgatorio, Nicola Gambacorta, Francesco Samarelli, Gianfranco Lopopolo, Modesto de Candia, Marco Catto, Orazio Nicolotti, Cosimo D. Altomare

**Affiliations:** Department of Pharmacy—Pharmaceutical Sciences, University of Bari Aldo Moro, Via Orabona 4, 70125 Bari, Italy; rosa.purgatorio@uniba.it (R.P.); nicola.gambacorta1@uniba.it (N.G.); francesco.samarelli@uniba.it (F.S.); gianfranco.lopopolo@newchemspa.it (G.L.); marco.catto@uniba.it (M.C.); orazio.nicolotti@uniba.it (O.N.); cosimodamiano.altomare@uniba.it (C.D.A.)

**Keywords:** antithrombotic agents, *N*,*N*′-malonamides, fXa inhibitors, thrombin inhibitors, acetylcholinesterase, butyrylcholinesterase, Alzheimer’s disease

## Abstract

The rational discovery of new peptidomimetic inhibitors of the coagulation factor Xa (fXa) could help set more effective therapeutic options (to prevent atrial fibrillation). In this respect, we explored the conformational impact on the enzyme inhibition potency of the malonamide bridge, compared to the glycinamide one, as a linker connecting the P1 benzamidine anchoring moiety to the P4 aryl group of novel selective fXa inhibitors. We carried out structure–activity relationship (SAR) studies aimed at investigating *para*- or *meta*-benzamidine as the P1 basic group as well as diversely decorated aryl moieties as P4 fragments. To this end, twenty-three malonamide derivatives were synthesized and tested as inhibitors of fXa and thrombin (thr); the molecular determinants behind potency and selectivity were also studied by employing molecular docking. The malonamide linker, compared to the glycinamide one, does significantly increase anti-fXa potency and selectivity. The *meta*-benzamidine (P1) derivatives bearing 2′,4′-difluoro-biphenyl as the P4 moiety proved to be highly potent reversible fXa-selective inhibitors, achieving inhibition constants (*K*_i_) in the low nanomolar range. The most active compounds were also tested against cholinesterase (ChE) isoforms (acetyl- or butyrylcholinesterase, AChE, and BChE), and some of them returned single-digit micromolar inhibition potency against AChE and/or BChE, both being drug targets for symptomatic treatment of mild-to-moderate Alzheimer’s disease. Compounds **19h** and **22b** were selected as selective fXa inhibitors with potential as multimodal neuroprotective agents.

## 1. Introduction

Cardiovascular diseases (CVDs) and coronary artery disease are the most prominent groups of complex disorders, causing high rates of morbidity and mortality worldwide [1]. About 80% of mortality in men and 75% in women resulted from CVDs; according to World Health Organization (WHO), approximately 18 million people died from CVD in 2019, among which, 85% were primarily due to heart attack and stroke [2]. In the past, using structure-based drug design strategies, new ‘direct oral anticoagulants’ (DOACs) were discovered, which act as selective inhibitors of the blood coagulation factors, namely factor Xa (fXa) and thrombin (thr) [3]. Some of them entered the clinic in patients with coronary artery disease, venous thromboembolism, and atherothrombosis, often associated with atrial fibrillation [4].

DOACs represent valid alternatives to low molecular weight heparins (LMWHs) [5], orally-administered warfarin, and other vitamin K antagonists (VKAs). The latter is characterized by a narrow therapeutic window and wide interactions with other administered drugs and food, requiring continuous dosing monitoring to ensure efficacy and avoid bleeding risks [6,7].

**Figure 1:**
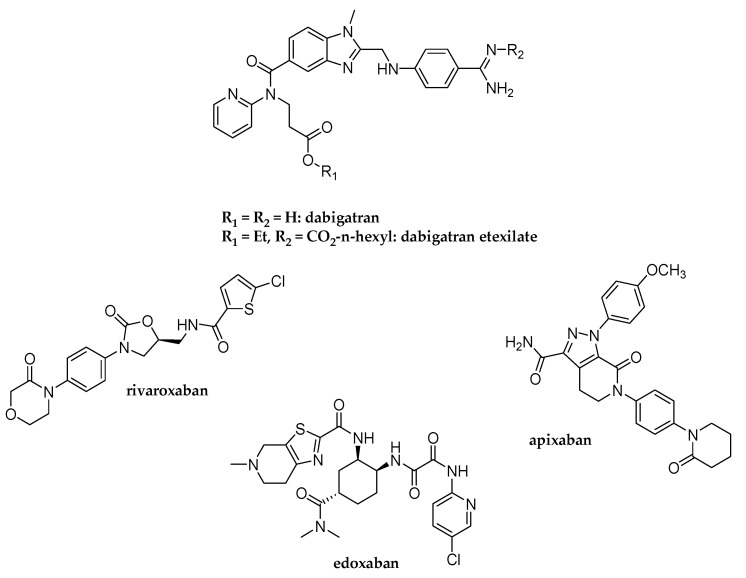
Approved direct fXa and thr inhibitors for oral administration (DOACs).

Currently approved DOACs (Figure 1) are dabigatran (available as the etexilate prodrug (Pradaxa), which acts as an inhibitor of both free and fibrin-bound thrombin), as well as apixaban (Eliquis), rivaroxaban (Xarelto), and edoxaban (Lixiana), which instead are factor Xa (fXa)-selective reversible inhibitors [8,9].

DOACs find clinical applications in stroke prevention in patients with nonvalvular atrial fibrillation (AF) or following orthopedic replacement surgery (hip or knee). Rivaroxaban is also indicated for acute treatment and secondary deep venous thrombosis (DVT) and pulmonary embolism. DOACs can be administered in standard doses without continuous adjustment and overcome several shortcomings often associated with the anticoagulant treatment with VKAs. DOACs are not indicated in patients with severe renal failure or impaired liver disease. In addition, they must be used with caution in patients with renal impairment. Nevertheless, despite their good benefit/toxicity/risk profile, these drugs suffer from the common drawbacks of anticoagulant drugs, such as the risk of major bleeding, mostly in the GI tract [10]. Consequently, novel anticoagulants that can effectively reduce thrombosis without an enhanced risk of bleeding are needed [11].

FXa and thr belong to the trypsin-like serine protease family and share high structural homology. The active domain shows a closed β-barrel fold with the catalytic triad Ser195-His57-Asp102. Additional essential subsites are responsible for selectivity against the two enzymes. Thr active site encompasses four distinct essential subsites S1−S4 [12]. The S1 site is a deep pocket containing Asp189 at the bottom, which may interact with Arg residue in the substrate or benzamidine and other basic groups in the synthetic ligands. A proximal S2 pocket (Tyr60A, Pro60B, Pro60C, and Trp60D loop 60A−D) sterically hinders the cavity of the active site, allowing only accommodation of small-sized hydrophobic substrate moieties. The flat S3/S4 is a large region lined by the hydrophobic residues Leu99, Ile174, and Trp215, recognizing large and lipophilic or aromatic moieties. The main differences between the thr and fXa active sites are the absence of the S2 in fXa and a different feature of the S4 subsite, which in fXa is a cylinder box-shaped aromatic cavity, lined by Tyr99, Phe174, and Trp215. As a consequence, selective fXa ligands can adopt an L-shaped binding conformation [13,14]. The identification of a suitable central scaffold, able to direct fragments in engaging interactions within the selectivity enzyme pockets, represents an important focus in the structure-based design of inhibitors of the blood coagulation fXa and thr.

In this study, we focus on the identification of new selective inhibitors of fXa, possibly targeting other serine proteases involved in neurodegenerative diseases, namely cholinesterases (ChEs). Due to the position of fXa in the coagulation cascade, its selective inhibitors are considered effective options in the treatment of venous thromboembolism and stroke prevention in patients with atrial fibrillation. FXa inhibitors prevent the generation of the new thrombin without affecting the basal thrombin level, thus ensuring primary hemostasis [13,14]. Compared to the classical antithrombotic drugs (heparins and warfarin), and direct thrombin inhibitors, fXa selective inhibitors should be characterized by lower bleeding risks.

Malonamide derivatives (MAMDs) are privileged chemical structures in drug design, widely employed to obtain peptidomimetic and chelating compounds [15]. MAMDs occur in many natural products and pharmaceuticals [16], and have been exploited to identify antidiabetics, k-opioid receptor agonists, and anticancer agents.

The malonamide bridge can be considered similar to the glycine residue in which one of the peptide bonds has been inverted [17]. Such a reversed amide link contributes toward maintaining chemical properties, stereochemical features, and binding modes similar to those of the parent peptide; these isomeric forms can however explore diverse conformational spaces [18]. Moreover, the amide retro-inversion can affect both pharmacodynamic and pharmacokinetic profiles, leading (in most cases) to an increase in target selectivity, a reduction in off-target undesired activities, and enhanced proteolytic resistance to proteases and CYP (cytochrome P)-mediated metabolism. The crossing of BBB, due to the change in the polar surface area, has also been reported. We recently described several peptidomimetic derivatives, which selectively inhibit fXa or thr depending upon the scaffold and moieties able to bind the specificity subsites S1−S4 of both enzymes [12,14,19,20,21].

MAMDs can be considered as retro-inverted analogues of the tripeptide sequence Phe-Gly-Arg recognized by fXa. Herein, we focus on the synthesis and activity of new compounds built on the malonamide bridge as the molecular core, linking a benzamidine as an arginine-mimetic moiety (P1 moiety) and varying the aromatic/lipophilic P4 fragments. The newly synthesized compounds were firstly assayed as fXa/thr inhibitors and compared with the corresponding glycinamide analogues.

## 2. Chemistry

**Scheme 1:**
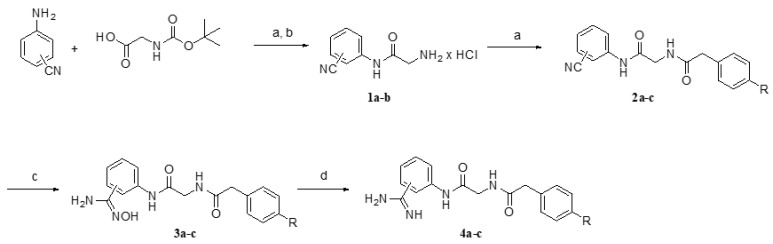
Reagents and conditions: (**a**) DCC, HOBt, TEA, dry THF, r.t.; (**b**) HCl solution in dioxane (4.0 M); (**c**) H_2_NOH×H_2_O, Na_2_CO_3_, abs. EtOH, reflux; (**d**) Pd/C (10%), EtOH/H_2_O (1:1), HCl (1N).

**Scheme 2:**
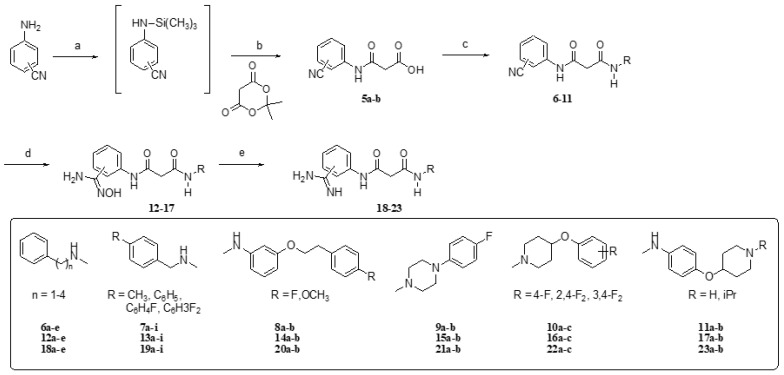
Reagents and conditions: (**a**) HMDS, K10 Montmorillonite, CH_2_Cl_2_, r.t.; (**b**) dry THF, reflux; (**c**) DCC, HOBt, TEA, dry THF, r.t.; (**d**) H_2_NOH×H_2_O, Na_2_CO_3_, abs. EtOH, reflux; (**e**) Pd/C (10%), EtOH/H_2_O (1:1), HCl (1N).

The newly synthesized glycinamide (GlyNH_2_) and malonamide (MalNH_2_) derivatives, containing a benzamidine P1 group, were obtained by following the reaction pathway shown in Scheme 1 and Scheme 2 [15]. The N-cyanophenyl GlyNH_2_ derivatives **2a–b** were synthesized by condensation of 3-(or 4-) aminobenzonitrile with N-BOC-glycine, by using dicyclohexylcarbodiimide (DCC), hydroxybenzotriazole (HOBt), and triethylamine (TEA).

After the removal of the BOC protecting group, the obtained hydrochloride salts **1a–b** were coupled with suitable phenylacetic acids as the P4 element. The intermediates **2a–c** underwent a two-step transformation. They were converted to the corresponding hydroxyimino derivatives **3a–c** by reacting with hydroxylamine hydrochloride and sodium carbonate in abs. EtOH, and in the second step, underwent a catalytic reduction in an acidic hydroalcoholic mixture (EtOH: water, 2:1 *v*/*v*; dilute HCl), using palladium on carbon (10% Pd-C) to obtain the final benzamidine derivatives **4a–c**.

The benzamidine-bearing MalNH_2_ derivatives were synthesized by the reactions shown in Scheme 2. The 3- (or 4-) cyanomalonamic acids **5a–b** were initially prepared by refluxing Meldrum’s acid and suitable aminobenzonitrile in dry toluene, leading to desired malonamic acids in low yield due to thermal decomposition and formation of the cyanoacetanilides. They were alternatively prepared in two sequential steps and a satisfactory yield without decomposition under mild conditions. As shown, 3 or 4-aminobenzonitrile were activated as sylilanilide, by treating them overnight with hexamethyldisilazane (HMDS) and K10 montmorillonite in methylene chloride at room temperature. The crude products were used without any purification in the aminolysis of Meldrum acid, carried out under reflux in dry THF. The malonamic acids **5a–b** were then coupled with amines (synthesized as reported elsewhere, when not commercially available). Finally, the cyano malonamide intermediates **6–11** were converted in benzamidino derivatives **18–23** in two steps, as above described in Scheme 1.

## 3. Results and Discussion

### 3.1. In Vitro Antithrombotic Activity

All the newly synthesized glycinamide and malonamide derivatives were evaluated for in vitro inhibition activity against fXa and thr, and the data, expressed as inhibition constants (K_i_), are listed in Table 1, Table 2, Table 3 and Table 4. The K_i_ values were calculated by applying the Cheng−Prusoff equation, starting from the IC_50_ values, based on dose–response curves through standard spectrophotometric assays. Apixaban and dabigatran, as selective inhibitors of fXa and thr, respectively, were used as positive controls.

In designing the novel peptidomimetic analogs of the Phe-Gly-Arg sequence, which is selectively recognized by fXa, and not by thr, the guanidino propyl chain of Arg was replaced by the basic pharmacophore *meta-* and *para*-benzamidine groups (P1). The P1 basic groups were connected to the unsubstituted benzyl moiety as P4 moiety through a GlyNH_2_ or a MalNH_2_ linker. The GlyNH_2_ analogues **4a** and **4b**, bearing *meta-* and *para*-benzamidine functioning as the P1 group, respectively, and the unsubstituted benzyl moiety as P4 moiety, as well as the corresponding MalNH_2_ derivatives **18a** and **18b**, were evaluated for their anti-fXa and anti-thr activities (Table 1).

GlyNH_2_ compounds **4a** and **4b** elicited a weak inhibition of both fXa and thr. The *para*-amidine isomer **4a** was less active than the *meta*-amidino isomer **4b**, which in turn resulted equipotent against fXa (K_i_ = 25.6 μM) and thr (K_i_ = 24.1 μM). The malondiamide analogues **18a–b** were found as fXa selective inhibitors. The position of the amidine group yet affected the activity profile, as the *meta*-substituted **18b** proved to be two times more potent than the *para* isomer **18a**. Interestingly, in addition, we tested succinyl analogues of the bis-malonamide **18a–b** (data in Supplementary Information), which showed a dramatic loss of activity against both the target enzymes, as inhibition of less than 10% was achieved at the highest tested concentration (50 μM). This clearly proved that the length and conformation of the carbon chain linking the P1 and P4 pharmacophores significantly affect the recognition of the binding sites.

On this basis, a series of new MalNH_2_ derivatives was synthesized by exploring the P4 feature (Table 2), as, to date, to the best of our knowledge, no similar investigation has been carried out [13,22]. Derivatives **18c–e** were designed to investigate the effect of the carbon chain length on N^3^ phenylamide. As a result, elongation from benzyl to phenylbutyl shifted selectivity toward thr inhibition. The potency was enhanced by increasing the alkyl chain length, and the highest inhibitory activity was achieved in compound **18e**, which contains a butyl spacer.

Investigation of N^3^-(benzyl)malonamide showed that substituents on the P4 moiety affected the inhibition profile. Introduction of the 4-methyl group in **19a–b** proved moderate inhibition of fXa, and weak activity against thr. However, they were about 3–4-fold more active than the corresponding unsubstituted analogues **18a–b**, without exhibiting differences between meta- and para-substituted amidino derivatives.

We envisaged that fXa/thr inhibition potency can be favorably affected by introducing a second phenyl ring in P4 moiety. The positional amidino isomers of unsubstituted biphenyl derivatives **19c** and **19d** showed significantly different activity, clearly indicating that, as a mimetic of the Arg side chain, the *meta*-benzamidine group works better than the *para*-substituted isomer. The *para*-amidino derivative **19c** showed a dramatic loss of activity against both fXa and thr, whilst the *meta* congener **19d** showed enhanced anti-fXa activity. It clearly appeared that the more rigid and oriented aromatic biphenyl fragment, which can become stuck inside the S4 aromatic box of fXa, could become a fXa selectivity key feature, efficaciously cooperating with the P1 meta amidino group.

These results prompted us to investigate the effect due to the introduction of one or more fluorine atoms on the distal phenyl ring (Table 2). Except for the 4-F derivative **19g**, the new fluorinated derivatives did increase fXa inhibition potency and selectivity. The fluorine substitution pattern deeply affected the activity profile. The 2-F (**19e**, K_i_ = 0.93 μM) and 4-F (**19g**, K_i_ = 0.91 μM) congeners were equipotent, achieving fXa inhibition at submicromolar concentration. The 3-F derivative **19f** (K_i_ = 0.35 μM) enhanced anti-fXa potency and selectivity. The introduction of a second F atom favorably affected the activity. The disubstitution with fluorine atoms at positions 2,4- proved to be the most effective, thus resulting in compound **19h** as the best in the series (K_i_ = 0.057 μM). The F-disubstitution at positions 3 and 5 furnished the analogue **19i**, with activity close to that of 3-F analogue **19f**.

We further compared compound **19h** and the corresponding GlyNH_2_ derivative **4c** (Table 3), showing once again that the replacement of the central glycinamide with the malonamide bridge did increase inhibition potency towards fXa, thus resulting in being the most advantageous in designing novel fXa selective inhibitors.

The anti-fXa activity decreased about 20 times from the best decorated malonamide **19h** to glycinamide **4c**, along with a loss of selectivity over thr, as well as the α-chymotrypsin, taken as a further representative enzyme of the serine protease family. The investigation of steric hindered on the C2 atom of MAMDs, by introducing two geminal methyl groups (**19l**), resulted in a loss of inhibition activity of both fXa and thr.

We further investigated alternative F-containing P4 groups, which differed in electronic, lipophilicity, and bulkiness properties (Table 4).

Replacement of biphenyl with phenyl-ethyloxy fragment led to compound **20a-b** (Table 4). The 4-F derivative **20a** (fXa K_i_ = 0.413 μM) showed moderate-to-good selective inhibition of fXa, one order of magnitude less active than the most active **19h**. Surprisingly, the 4-OCH_3_ bearing analogue **20b** (fXa, K_i_ = 0.205 μM) enhanced the two-time activity against fXa, without changing selectivity over thr and *α*-chymotrypsin.

A 4-fluorinated phenylpiperazine moiety introduced a tertiary amide and did not improve fXa inhibition (**21a** proved to be almost equipotent with **20a**). The F-substituted phenoxy-piperidine P4 fragment elicited derivatives endowed with relevant and selective anti-fXa properties, and the F atoms pattern affected activity. The 4-F analogue **22a** was three times less potent than 2,4-F_2_ substituted congener **22b**, which achieved fXa inhibition close to that of the above-mentioned 2,4-F_2_ biphenyl derivative **19h**. The 3,4-F_2_ substitution resulted in a slight loss of potency, with compound **22c** one-half less active than **22b**. Finally, the reversal of phenoxy-piperidine from N-linked to O-linked achieved in compound **23a** a moderate fXa inhibition, weak activity against thrombin, and complete absence of α-chymotrypsin inhibition. Introducing an isopropyl group on piperidine significantly reduced potency against fXa.

In silico calculated physicochemical properties (Table S1) are within the applicability domain and comply with the “Lipinski’s Rule of Five”. Based on calculations, derivatives **19h**, **19l**, **22b**, and **22c** were predicted by the ‘admetSAR’ software as able to cross the BBB, non-hepatotoxic, absorbed through the human intestine [23].

As far as metabolism prediction is concerned, they were predicted as CYP3A4 and 2C9 substrate (no inhibitors), and both substrate and inhibitors of Pgp. Compound **19h** was, in addition, predicted as less bound (about 25%) to the plasma protein than glycinamide **4c**. Compounds **19h**, **19l**, **22b**, and **22c** all passed the pan assay interference compounds (PAINS) filter [24].

### 3.2. Molecular Modeling

To understand the molecular rationale behind the observed affinity, we investigated by molecular docking simulation the binding to fXa of the most active MalNH_2_-containing inhibitors **19h** and **22b**, comparing them with the corresponding GlyNH_2_-based inhibitors **4c**. The molecular docking calculations, in agreement with the experimental observations, suggested that **19h**, due to the bismalonamide bridge connecting P1 and P4 moieties, can achieve, more easily than the glycinamide **4c**, an L-shaped conformation, which would allow the benzamidine P1 group and the 2,4-difluorophenyl P4 moiety, to bind more efficiently onto the respective S1 and S4 enzyme subpockets.

Ab initio calculation was carried out to reveal the lowest-energy tautomers and conformers for **19h** and **4c**, bearing malonamide and glycinamide bridges, respectively. As far as the tautomers are concerned, the single diketo form was predicted for compound **19h**, which is consistent with similar computational r reported elsewhere [25]. Interestingly, the analysis of conformers disclosed a slight but relevant difference between **19h** and **4c**. The former adopted a more bent conformation for the occurrence of an intramolecular HB, which is typical in the malonamide bridge [17]. The conformational feature showing opposing C=O groups was confirmed by the observed differences of 30–40 cm^−1^ between the carbonyl IR signals [26].

**Figure 2:**
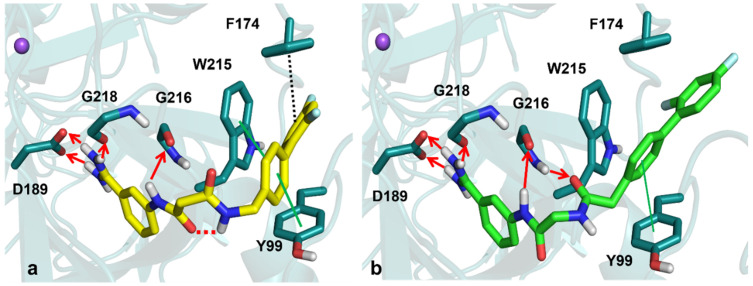
Panels (**a**,**b**) report the top-score docking poses of compounds **19h** and **4c**, respectively. Hydrogen bonds, π−π interactions, and hydrophobic contacts are represented as red arrows, and green and dashed black lines, respectively.

Molecular docking simulations and binding-free energy calculations were then performed to shed light on the interactions driving the inhibition of factor Xa. Top-scored docking poses are shown in Figure 2.

The amidine group of **19h** can strongly interact through HBs and electrostatic interactions with the carboxylate side chain of D189 at the S1 pocket, and with the backbone carbonyl of G218. In addition, the nitrogen atom of the malonamide bridge can make HB with the backbone carbonyl of G216, and the 2,4-fluorine-biphenyl moiety can engage hydrophobic contacts with W215, Y99, and F174 at the S4 subsite. Similarly, **4c** can establish a network of polar interactions through its amidine group with the carboxylate side chain of D189 at the S1 pocket, and with the backbone carbonyl of G218. Likewise, the glycinamide bridge can interact with G216, and the biphenyl moiety can make *π*–*π* interaction with Y99 at S4.

**Figure 3:**
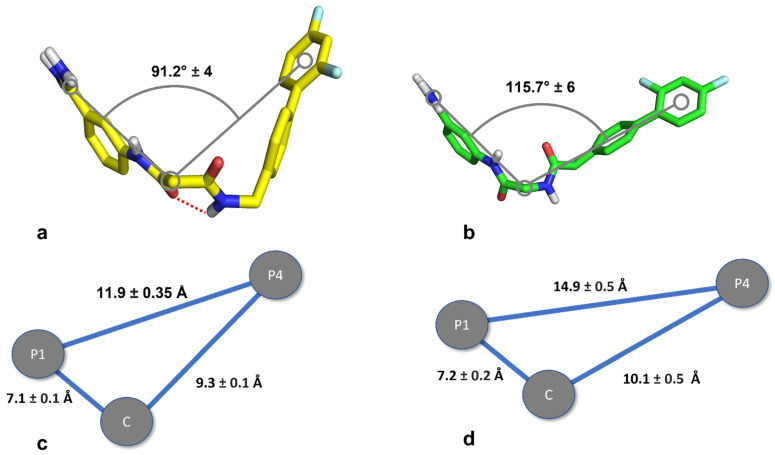
Panels (**a**,**b**) show the best conformer of **19h** and **4c** depicted as yellow and green sticks, respectively. The intramolecular HB is shown as dashed red lines. Joined by gray lines, the three gray circles indicate the anchoring point of the amidine group (P1), the center of malonamide/glycinamide bridge (C), and the centroid of the terminal phenyl of the P4 group. Panels (**c**,**d**) report the distances and the standard deviation among P1-C-P4 for both compounds.

Docking score and binding-free energy values were equal to −10.17 kcal/mol and −64.31 kcal/mol, for **19h** and −9.79 and −50.48 kcal/mol **4c**. Interestingly, as shown in Figure 3, a more detailed conformational analysis of protein–ligand complexes revealed that the intramolecular HBs (hydrogen bonds) of the **19h** malonamide bridge were crucial for its best fitting within the fXa S1 and S4 pockets, helping to generate an angle of 91.2 ± 4° (close to the right angle of a perfect L-shape), by considering three selected carbon atoms that were the centers of the amidine anchoring point (P1), the malonamide/glycinamide group (C), and the terminal phenyl ring of the biphenyl group (P4). On the opposite, the same angle computed for the glycinamide derivative **4c** was 115.7 ± 6°. Moreover, while the P1-C distance was the same for both compounds, the distance length between P1 and P4 showed the highest difference. A significant difference of 3 Å should exist in the P1−P4 distance, between **19h** and **4c**. Together, these geometrical features suggested that **4c** could not achieve the same optimal interaction of the malonamide derivative **19h** with both S1 and S4 pockets in fXa. This conformation-dependent impact of the malonamide bridge may support the rational design of new fXa-selective potent inhibitors.

Overall, from both energetic and geometric points of view, compound **19h** can better tether the binding pocket of fXa than **4c**, engaging critical interactions for its inhibition, this being in agreement with the experimental data.

### 3.3. Anti-Cholinesterase Activity

It is widely reported that elderly Alzheimer's disease (AD) patients present comorbidities, such as cardiovascular conditions, often associated with metabolic imbalance. The risk factors inducing thrombotic events (i.e., hypertension, diabetes, and hyperlipidemia) also increased the risk of thrombotic events, cerebrovascular lesions, and cognitive decline in AD patients or other dementias. Furthermore, a putative role as an inflammatory mediator in the brain has been supposed for coagulation proteases thrombin and fXa. The deposition of A*β* protein plaques, including coagulation factors, may induce leaking of the brain−blood−barrier (BBB), promote a pro-thrombotic state, and induce the release of pro-inflammatory mediators in the brain. It has been recently demonstrated that dabigatran induced mitigation of neuroinflammation, reduction of the histopathological hallmarks of AD, and helped in the recovery of cognitive decline. By following a previously reported paper, [27], herein, we investigated the new promising fXa/thr MalNH_2_-based inhibitors, integrating additional anticholinesterases (ChEs) activity, to discover multimodal agents that inhibit AD-related enzymes, such as AChE and BChE, while maintaining their anticoagulant potency.

The malonamide derivatives showing the highest inhibition potency toward the blood coagulation serine proteases were tested against the acetyl-(AChE) and butyryl- (BChE) cholinesterases, which are related to the progression of AD and related neurodegenerative diseases. Taking into account molecular features critical to the inhibition of fXa and thr, the selected compounds present the amidine function in meta and diverse P4 fragments. The selected compounds were tested, applying the known Ellman method, and the results are shown in Table 5. The physicochemical properties of the P4 fragment deeply affected anticholinesterase activity.

Compounds bearing a P4 biphenyl group showed moderate inhibition of both AChE and BChE. The introduction of a fluorine atom on the distal phenyl did not affect the activity profile, without any difference depending on the F positional isomerism. The introduction of a second F atom with the 2′,4′-F_2_ substitution pattern enhanced BChE; thus, resulting in the selective inhibitor of fXa compound **19h**, endowed of inhibition of BChE (K_i_ = 1.52 μM) in the low micromolar range, as a potential multi-target drug ligand (MTDL) derivative. The combination of fXa and BChE inhibition in the same molecule must not be underestimated. Indeed, BChE has been suggested to be involved in the AD progression, whereas fXa inhibition can prevent the generation of thr and possibly its AD-related neurotoxicity and inflammation. Changing to 3′,5′-F_2_ in compound **19i** resulted in a significant loss of anticholinesterase activity, mostly against AChE, and to a lesser extent against BChE.

The introduction of a gem-dimethyl on C2 of the malonamide bridge did not affect cholinesterase inhibition, resulting in derivative **19l**, slightly close to its analogue **19h**, which indicates tolerance to steric hindrance in that position.

The phenethyloxyaniline as a P4 element was further investigated. The 4-F substituted compound **20a** showed good selective inhibition of BChE (K_i_ = 1.02 μM), whilst the replacement of F with a 4-methoxy group determined a complete reversal of selectivity, with compound **20b** exhibiting notable activity against AChE (K_i_ = 1.56 μM). The introduction of fluorophenyl piperazine as the P4 element did not contribute to the anti-ChE activity. Selective fXa inhibitors **22a–c**, bearing a phenoxypiperidine fragment, emerged as noteworthy inhibitors of AChE. The number and position of the fluorine atoms affected the anti-fXa without causing significant changes in the anticholinesterase activity, the most interesting being the 2,4-F_2_ compound **22b** (fXa K_i_ = 0.089 μM; AChE K_i_ = 2.31 μM). It was finally found that reversing the direction of linked phenoxypiperidine and alkylating piperidine nitrogen resulted in the good cholinesterase inhibitor **23b**, unfortunately lacking inhibition potency against fXa or thr.

**Figure 4:**
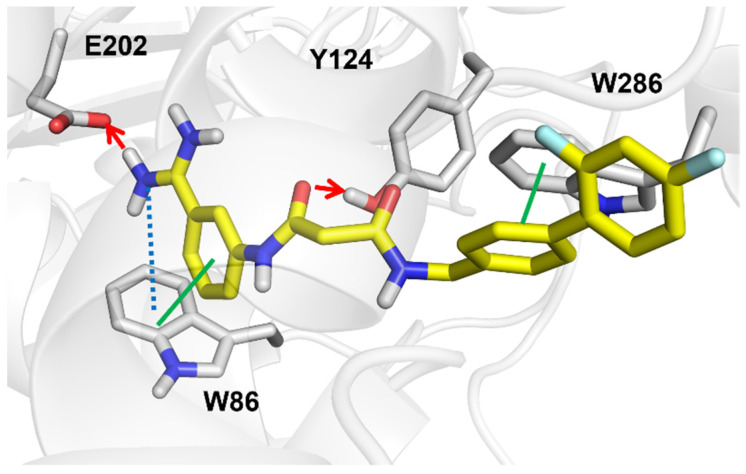
Zoomed in view at AChE binding site. The best **19h** docked pose is rendered in yellow sticks. Red arrows and blue and green dashed lines indicate hydrogen bonds, cation–*π*, and *π*–*π* interactions, respectively.

Finally, given the possible multi-target action of **19h**, molecular docking simulations were also performed within the AChE-binding pocket (Figure 4).

Compound **19h** can bind the AChE-binding pocket by engaging interactions with several key residues. More specifically, the amidine group can establish HB with the side chain of E202, and a cation–π interaction with W86 at CAS. In addition, the aromatic ring can interact with W86, forming the HB contact; the carbonyl group of the malonamide bridge can make HB with the sidechain of Y124 and the biphenyl group can engage aromatic contacts with W286 at PAS. Docking score and binding-free energy values were equal to −10.74 and −53.29 kcal/mol, respectively. Regarding the ligand conformational analysis, **19h** can be accommodated within the AChE-binding site by preferring an extended pose, in which the intramolecular hydrogen bond of the malonamide bridge is no longer observed. Noteworthy, such a binding pose engaged W86 and W286, which are key residues for AChE inhibition.

## 4. Materials and Methods

All chemicals and solvents were purchased from Sigma-Aldrich and Alfa Aesar. Melting points were determined by using the capillary method on a Stuart Scientific SMP3 electrothermal apparatus and were uncorrected. For solid final compounds, the purity was assessed by elemental analyses (C, H, N), performed on a Euro EA3000 analyzer (Eurovector, Milan, Italy), by the Analytical Laboratory Service of the Department of Pharmacy-Drug Sciences of the University of Bari Aldo Moro (Italy), and the results agreed to within 0.4% of theoretical values. All of the tested compounds showed higher than 95% purity. Mass spectra were obtained by Agilent 1100 Series LC–MSD Trap System VL, equipped with an electrospray ionization (ESI) source. The high-resolution molecular masses of the test compounds were assessed by Agilent 6530 Accurate Mass Q-TOF spectrometer (Agilent Technologies, Santa Clara, CA, USA). IR spectra (KBr disks) were recorded on a Spectrum One FT infrared spectrophotometer (PerkinElmer, Beaconsfield, UK), and the most significant absorption bands are listed; 1H NMR spectra were recorded at 300 MHz on a Varian Mercury 300 instrument. Chemical shift data and the coupling constants J are in hertz (Hz); the following abbreviations are used for multiplicity: s, singlet; d, doublet; dd, doublet–doublet; td, triplet of doublets; t, triplet; q, quartet; m, multiplet. Signals due to NH and OH protons were located by deuterium exchange with D_2_O. Chromatographic separations were performed on silica gel 60 for column chromatography (Merck 70–230 mesh).

### 4.1. Chemistry

#### 4.1.1. General Procedure for the Synthesis of Compounds **2a**–**c**

The synthesis of compound **2c** is reported as an example. Hydroxybenzotriazole (HOBt, 1 mmol), N,N′-dicyclohexylcarbodiimide (DCC, 1.1 mmol), and after 20 min, the triethylamine (2.5 mmol) and N-BOC-glycine (1 mmol), were added to a solution of 4-aminobenzonitrile (1 mmol) in 15 mL of dry THF. The reaction mixture was stirred at room temperature for two days and filtered. The obtained solution was extracted twice with ethyl acetate, and the collected organic phases were washed with Na_2_CO_3_ 10% aqueous solution, 2M HCl, and brine. The organic phase was dried over anhydrous Na_2_SO_4_, filtered, and concentrated under reduced pressure. The resulting crude product was purified by flash chromatography on silica gel to achieve the desired amide compound. After the removal of the N-BOC protecting group (4M HCl in dioxane), the hydrochloride salts were used in the next coupling reaction.

*2-amino-N-(3-cyanophenyl)acetamide hydrochloride* (**1b**). [ESI-MS], *m/z*: 192.13 [M+H]^+^ for C_10_H_14_N_3_O.

Compound **1b** (1 mmol) was then coupled (2′,4′-difluorobiphenyl-4-yl) acetic acid (1 mmol), by using the same procedure as before (DCC, HOBt, and TEA in anhydrous THF). The crude product was purified by chromatography on silica gel by eluting with a mixture n-hexane/ethyl acetate 30/70 *v*/*v*, to achieve compound **2c**.

*3-cyano-N-[3-(2*′,*4*′*-difluorophenyl-4-yl)-2-oxopropyl]benzamide* (**2c**). Dark brown oil, yield 45% (100 mg). IR cm^−1^: 3296, 3077, 2233, 1657, 1551, 1494, 1404, 1141, 1100, 964, 849, 804, 682. ^1^H NMR (300 MHz, CDCl_3_), δ_H_ 8.14 (t, J = 1.7 Hz, 1H), 8.03 (d, J = 7.7 Hz, 1H), 7.79 (d, J = 7.7 Hz, 1H), 7.49–7.32 (m, 7H), 6.98–6.87 (m, 2H), 6.65 (t, J = 5.2 Hz, 1H), 4.53 (d, J = 5.8 Hz, 2H), 4.18 (d, J = 5.0 Hz, 2H).

#### 4.1.2. General Procedure for the Synthesis of Compounds **5a**–**c**

The synthesis of malonamic acid **5b** has been reported as an example. A total of 2.13 mL (10.2 mmol) of hydroxymethyl disilazane (HMDS) and a catalytic amount (0.2 g) of Mont K10 were added to a solution of 1 g (8.45 mmol) of 4-aminobenzonitrile in 20 mL of dry methylene chloride (DCM). The reaction mixture was stirred under reflux for 24 h; after cooling, it was filtered on a celite pad and concentrated to dryness under reduced pressure. The obtained oil residue was solubilized in 30 mL of dry tetrahydrofuran (THF) and 1.22 g (8.45 mmol) of Meldrum’s acid (2,2-Dimethyl-1,3-dioxane-4,6-dione) was added to the solution. The mixture was stirred under reflux for 3 h. After cooling, 15 mL of methanol was added and the mixture was stirred for 30 min. After removal of the solvent under reduced pressure, the residue was suspended in ethyl ether and obtained after filtration.

*3-[(3-cyanophenyl)amino]-3-oxopropanoic acid* (**5b**). Brown solid, yield 72% (1.25g), M.P. 96–99 °C. IR cm^−1^: 3023, 2914, 2228, 1709, 1668, 1587, 1295, 796. ^1^H NMR (300 MHz, DMSO-d_6_), δ_H_ 12.01 (s, 1H), 10.47 (s, 1H), 8.06 (s, 1H), 7.78 (m, 1H), 7.52 (m, 2H), 3,39 (s, 2H).

Compounds **5a** and **5c** were obtained by following the same procedure, and by using 4-aminobenzonitrile and Meldrum’s acid, and 3-aminobenzonitrile and tetramethyl-1,3-dioxane-4,6-dione, respectively.

#### 4.1.3. General Procedure for the Synthesis of Malonamide Derivatives **6**–**11**

The synthesis of compound **7h** is reported as an example. Hydroxybenzotriazole (HOBt, 1 mmol), N,N’-dicyclohexylcarbodiimide (DCC, 1.1 mmol), and after 20 min, triethylamine (TEA, 2.5 mmol) and 1-(2′,4′-difluorobiphenyl)-4-yl)methanamine hydrochloride (1 mmol) were added to a solution of the compound **5b** (1 mmol) in 15 mL of dry THF. The reaction mixture was stirred at room temperature for two days and filtered. The obtained solution was extracted twice with ethyl acetate, and the collected organic phases were washed with Na_2_CO_3_ 10% aqueous solution, 2M HCl, and brine. The organic phase was dried over anhydrous Na_2_SO_4_, filtered, and concentrated under reduced pressure. The resulting crude product was purified by flash chromatography on silica gel to achieve the desired amide compound.

*N-(3-cyanophenyl)-N’-[(2*′,*4*′*-difluorobiphenyl-4-yl)methyl]malonamide* (**7h**). Yellow oil, yield 32% (80 mg). IR cm^−1^: 3321, 2231, 1681, 1651, 1431, 964, 795. ^1^H NMR (300 MHz, CDCl_3_), δ_H_ 10.09 (s, 1H), 8.04 (s, 1H), 7.74–7.61 (m, 1H), 7.50–7.28 (m, 8H), 6.96–6.83 (m, 2H), 4.51 (d, J = 5.8 Hz, 2H), 3.45 (s, 2H).

All malonamide analogues were obtained by following the same procedure.

#### 4.1.4. General Procedure for the Synthesis of N-hydroxyamidine Derivatives

The synthesis of compound **13h** is reported as an example. To a solution of cyano derivative **7h** (0.2 mmol) in 2 mL of ethanol, hydroxylamine hydrochloride (0.76 mmol) and Na_2_CO_3_ (0.38 mmol) were added, and the reaction mixture was refluxed 6 h up to disappearance of the starting material (TLC). After cooling, the mixture was filtered and concentrated under reduced pressure to yield the desired hydroxyamidino intermediate, which was used in the next preparation step without any purification.

*N-(3[amino(hydroxyimino)methyl)phenyl]-N’-(2*′*,4*′*-difluoro)benzylmalonamide* (**13h**). White amorphous powder, yield 73% (70 mg). IR cm^−1^: 3258, 2928, 2850, 1637, 1591, 1561, 1494, 1448, 1141, 1100, 806, 789. ^1^H NMR (300 MHz, DMSO-d_6_), δ_H_ 10.18 (s, 1H), 9.60 (s, 1H), 8.63 (t, J = 6.0 Hz, 1H), 7.88 (s, 1H), 7.66–7.25 (m, 9H), 7.17 (td, J = 8.5, 2.6 Hz, 1H), 5.73 (s, 2H), 4.35 (d, J = 5.9 Hz, 2H), 3.32 (s, 2H).

All the hydroxyimino derivatives **3** and **12–17** were obtained by following the same procedure.

#### 4.1.5. General Procedure for the Synthesis of Benzamidine Derivatives

The synthesis of compound **19h** is reported as an example. A total of 50 mg of palladium on carbon (10% Pd-C) and 1M HCl (200 μL) were added to a mixture containing hydroxyimino derivative 13h (0.1 mmol), in 15 mL of a 2/1 *v*/*v* EtOH/water mixture. The suspension was kept under a hydrogen atmosphere at normal pressure (3 bars) up to the complete transformation of the starting material (24 to 72 h, TLC). After completion, the suspension was filtered on a celite pad, and the filtrate was concentrated under reduced pressure and further washed with absolute ethanol, to afford the desired product as the hydrochloride salt.

*N-(3[amino(imino)methyl)phenyl]-N’-(2*′*,4*′*-difluoro)benzylmalonamide hydrochloride* (**19h**). Brown oil, yield 75% (170 mg). IR cm^−1^: 3399, 1895, 1672, 1638, 1602, 1555, 1499, 1433, 1251, 805, 721, 676. ^1^H NMR (300 MHz, DMSO-d_6_), δ_H_ 10.79 (s, 1H), 9.40 (s, 2H), 9.21 (s, 2H), 8.80 (t, J = 5.5 Hz, 1H), 7.7–7.0 (m, 11H), 4.33 (d, J = 5.8 Hz, 2H), 3.35 (s, 2H). HRMS [ESI], m/z: 423.1633 [M+H]^+^ for C_23_H_21_N_4_O_2_F_2_.

All the benzamidino derivatives **4** and **18–23** were obtained by following the same procedure.

### 4.2. Enzymatic Assays

#### 4.2.1. Coagulative Serine Proteases Thrombin and fXa Inhibition Assay

The test compounds were evaluated in vitro for their inhibitory activity toward fXa and fIIa, determining the hydrolysis rates of the synthetic chromogenic substrates, monitored at 405 nm [27]. Enzymes and substrates were used as follows, in final concentrations: 2 nM human fXa and 0.04 mM S-2765 (Z-D-Arg-Gly-Arg-p-NA) from Chromogenix ABInstrumentation Laboratories (Milan, Italy), 0.41 U×mL^−1^ bovine thrombin from Sigma-Aldrich (Milan, Italy), and 0.05 mM S-2238 (D-Phe-Pip-Arg-p-NA) from Chromogenix AB-Instrumentation Laboratories (Milan, Italy). Enzyme solutions were incubated with DMSO solutions of the test inhibitor (DMSO did not exceed 1%) in various concentrations (0.001–50 μM) before the respective chromogenic substrates were added to start the enzyme kinetics. The kinetic studies were performed at pH 8. Reactions were initiated by adding 100 μL of substrate solutions and monitoring the increase in absorbance for 5 min.

The initial velocities were determined within 60 s and the concentration of the inhibitor required to diminish by 50% of the control velocity (IC_50_), calculated by nonlinear (sigmoid) regression. Three independent IC_50_ values were determined for calculating inhibition constants Ki using the Cheng–Prusoff equation [28].

#### 4.2.2. Cholinesterases Inhibition Assay

The test compounds for their inhibitory activity toward AChE (from electric eel) and BChE (from horse serum) were also evaluated by applying Ellman’s assay with some modifications [29,30]. The anti-AChE activity was determined in a reaction mixture containing 20 μL of a solution of AChE (0.9 U/mL in 0.1 M pH 8.0 phosphate buffer, PB), 20 μL of a solution of 5,5-dithio-bis-(2-nitrobenzoic) acid (DTNB 3.3 mM in 0.1 M pH 7.0 PB, containing 0.1 mM NaHCO_3_), 20 μL of a solution of the test compound (five to seven concentrations, ranging from 1×10^−4^ to 1×10^−10^ M in 0.1 M pH 8.0 PB), and 120 μL of pH 8.0 PB. After incubation for 20 min at 25 C, acetylthiocholine iodide (20 μL of 0.05 mM water solution) was added as the substrate; the hydrolysis rates of the substrate were monitored at 412 nm for 5.0 min at 25 C, and the initial reaction rate was determined within 60 s. The concentration of the inhibitor required, diminished by 50% of the rate of the control (IC_50_), was calculated by nonlinear (sigmoid) regression of the response/concentration (log) curve, using Prisma GraphPad software (v. 5.01). From three independent IC_50_ values, the inhibition constants Ki were calculated by using the Cheng–Prusoff equation [15]. Relative SEM values (standard error of means) were all found to be less than 5% of the means. BChE inhibitory activity was determined similarly, by using a solution of BChE (1.8 U/mL in 0.1 M pH 8.0 PB) and butyrylthiocholine iodide (0.05 mM) as the substrate.

### 4.3. Molecular Modeling

X-ray-solved structures of FXA and AChE were obtained from the Protein Data Bank by retrieving entries 3K9X and 4EY7, respectively [31,32]. The protein preparation wizard was employed to refine the crystal structures, carry out steps of energy minimization, and remove non-structural water molecules.

The 3D structures of ligands to be docked were first optimized with the OLPS3 force field [33] and, subsequently, the ab initio generation of tautomers (using water as solvent without charge adjustment, by identifying and delocalizing the proton donor and acceptor atoms within the molecules), and conformers were performed by means of the QM tautomers and the conformers package of Jaguar software [34,35], in a six-step workflow, as follows: (1) a list of tautomers was generated by identifying and delocalizing the proton donor and acceptor atoms within the molecules; (2) a ranking of generated tautomers was performed according to the PM3 heat of formation and the high energy tautomers were excluded; (3) conformers were generated from the survived tautomers by using the MacroModel package [36]; (4) the resulting conformers were filtered, again, according to the PM3 heat of formation, and high energy structures were discarded; (5) DFT geometry optimizations were performed on the selected structures using the B3LYP-D3/LACVP** level of theory; (6) the structures were ranked using single-point energies at the M06-2X/cc-pVTZ(-f) level of theory calculated at the optimal geometries from the previous step. Finally, the top five conformers for each ligand were used for molecular docking analyses. In this respect, the enclosing grid box was centered on the center of mass of the cognate ligands, and the standard precision (SP) docking protocol was employed with default settings by using GLIDE [37]. To validate such docking protocols, redocking analyses were performed on the cognate ligands within their respective binding pockets. Satisfactorily, the root mean square deviation (RMSD) computed on the heavy atoms between the original and the best-docked poses were equal to 0.99 and 0.42 Å for factor Xa and AChE, respectively. In addition, starting from the lowest energy-docked pose, the Embrace Conformational Search panel available in the MacroModel package [6] was used for performing a conformational exploration of a ligand–receptor complex. In detail, the mixed torsional/low sampling method was used and the OPLS3 force field was applied. A region with freely moving atoms was selected, including ligand and receptor residues within 3 Å from the former, whereas all atoms beyond 5 Å from the ligand were considered constrained. The number of outputs was set to 50, and all redundant conformers with maximum atom deviations of 0.5 Å were discarded. A total of 30 compounds were finally considered. Eventually, the molecular mechanics/generalized born surface area (MM-GBSA) method [38], as implemented in Prime [35], was included in the workflow in order to compute the binding-free energies (ΔG_bind_) between the ligands and protein. For completeness, the ΔG_bind_ was computed as follows:

ΔG_bind_ = ΔE_MM_ + ΔG_solv_ + ΔG_SA_

where ΔE_MM_ is the minimized energy of the ligand–protein complex, ΔGs_olv_ is the solvation energy, and ΔG_SA_ is the binding energy of the surface area of the compounds.

## 5. Conclusions

There is compelling evidence for a relationship between atrial fibrillation and AD-related dementia in elderly populations. The amount-dependent role of thr in a brain injury, which could cause (at higher concentrations) neuroinflammation and apoptosis, as well as the involvement of thr in several diseases, including AD and cerebral ischemia, led us to assess the effects of DOACs in preclinical models of cognitive impairments. As a major outcome, the thr-selective potent inhibitor dabigatran proved to be a promising candidate for clinical studies aimed at extending its indications for the treatment of AD cognitive decline.

In this context, we evaluated in vitro the fXa/thr inhibitory activity of newly synthesized peptidomimetics of the tripeptide substrate sequence Phe-Gly-Arg, selectively recognized by fXa, in which the benzyl side chain of Phe (interacting with the subsite S4 of fXa) has been replaced by arylalkyl and biphenyl moieties, the basic Arg side chain (interacting with Asp189 into the S1 pocket) replaced by the benzamidine group, and the central Gly replaced by a malonyl residue, which involves a retro-inversion of the first peptide bond between the *N*-terminal Phe mimetic and Gly. Compound **19h**, bearing the malonamide bridge linking a *meta*-benzamidine and a 2,4-F_2_-biphenyl moieties showed, selectively, the strongest fXa inhibition.

Interestingly, some of the most potent inhibitors of fXa (e.g., **19h** and **22b**) achieved single-digit micromolar inhibition potency against AChE and/or BChE, which are well-known drug targets for symptomatic treatment of mild-to-moderate AD. The combination of the two inhibitory activities against fXa and ChEs may be beneficial. Anticoagulant therapy with DOACs may reduce cardiovascular risk factors in the development of the AD-related dementias, whereas inhibitors of AChE and/or BChE may restore the cholinergic transmission in the AD brain at different disease stages. In this study, the bis-malonamide derivative **19h**, with *K*_i_ against fXa equal to 57 nM and AChE/BChE inhibition potency in the very low (single-digit) micromolar range, may be considered a hit compound for further optimization studies and ex vivo/in vivo pharmacological investigations in animal models of AD-like neurodegeneration syndromes.

## Figures and Tables

**Table 1:** FXa and thrombin inhibition of glycinamide versus malonamide analogues.

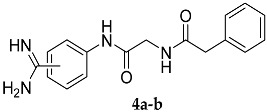		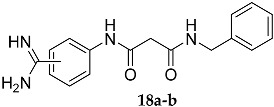	
Cmpd	Amidine Position	*K*_i_ (μM) ^a^	
		fXa	thr
**4a**	*p*	>50	39.4
**4b**	*m*	25.6	24.1
**18a**	*p*	23.2	>50
**18b**	*m*	12.3	>50
**Apixaban ^b^**		0.00012	
**Dabigatran ^b^**			0.0042

^a^ Ki values determined by applying the Cheng–Prusoff equation to IC_50_ values, by regression (GraphPad Prism software ver. 5.01). Data are the means of three independent measurements, each one performed in triplicate (SEM < 5%). ^b^ Apixaban and dabigatran were tested as positive controls against blood coagulation factors fXa and thrombin, respectively.

**Table 2:** Activity of *N*^1^-(amidinophenyl)-*N*^3^-benzylmalonamides on fXa and thr.

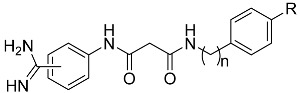						
Cmpd	Amidine Position	n	R	*K*_i_ (μM) ^a^		
				fXa	thr	αCHT ^c^
**18c**	*p*	2	H	>50	>50	
**18d**	*p*	3	H	39.8	25.5	
**18e**	*p*	4	H	18.6	5.50	
**19a**	*p*	1	4-CH_3_	4.40	36.5	
**19b**	*m*	1	4-CH_3_	4.20	36.6	
**19c**	*p*	1	C_6_H_5_	>50	>50	
**19d**	*m*	1	C_6_H_5_	1.56	>50	4.3
**19e**	*m*	1	2-F(C_6_H_4_)	0.932	14.6	>50
**19f**	*m*	1	3-F(C_6_H_4_)	0.348	18.8	26.5
**19g**	*m*	1	4-F(C_6_H_4_)	0.907	6.19	>50
**19h**	*m*	1	2,4-F_2_(C_6_H_3_)	0.057	16.1	>50
**19i**	*m*	1	3,5-F_2_(C_6_H_3_)	0.264	26.7	30.2

^a^ See footnotes in Table 1.

**Table 3:** Malonamide versus glycinamide comparison in the inhibition of the blood coagulation enzymes fXa and thr.

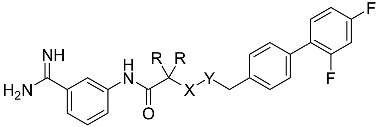					
Cmpd	X-Y	R	*K*_i_ (μM) ^a^		
			fXa	thr	αCHT
**4c**	NHCO	H	1.01	10.7	35.0
**19h**	CONH	H	0.057	16.1	>50
**19l**	CONH	CH_3_	0.214	>50	>50

^a^ See footnotes Table 1.

**Table 4:** Effects of replacement of the P4 group on inhibition of fXa, thr, and α-CHT.

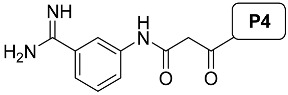					
Cmpd	P4	R	*K*_i_ (μM) ^a^		
			fXa	thr	αCHT
**20a**	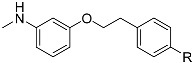	4-F	0.413	12.0	>50
**20b**		4-OCH_3_	0.205	6.66	>50
**21a**	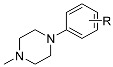	F	0.423	31.4	>50
**22a**	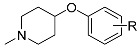	4-F	0.350	>50	>50
**22b**		2,4-F_2_	0.089	>50	>50
**22c**		3,4-F_2_	0.120	27.0	>50
**23a**	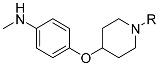	H	0.590	42.4	>50
**23b**		CH(CH_3_)_2_	2.30	>50	>50

^a^ See footnotes in Table 1.

**Table 5:** Inhibitory activity of selected malonamides on cholinesterases (eeAChE, eqBChE).

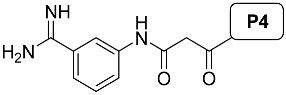				
Cmpd	P4	R	*K*_i_ (μM) ^a^	
			AchE ^b^	BchE ^b^
**19d**	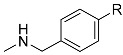	C_6_H_5_	9.33	(28%)
**19e**		2-F(C_6_H_4_)	(35%)	7.70
**19f**		3-F(C_6_H_4_)	8.11	8.09
**19g**		4-F(C_6_H_4_)	4.48	(37%)
**19h**		2,4-F_2_(C_6_H_3_)	2.44	1.52
**19l ^c^**		2,4-F_2_(C_6_H_3_)	3.74	1.55
**19i**		3,5-F_2_(C_6_H_3_)	(5%)	8.02
**20a**	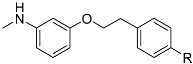	4-F	10.0	1.02
**20b**		4-OCH_3_	1.56	5.84
**21a**	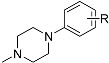	4-F	4.68	10.0
**22a**	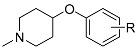	4-F	1.88	(22%)
**22b**		2,4-F_2_	2.31	(25%)
**22c**		3,4-F_2_	2.68	10.9
**23a**	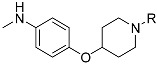	H	(1%)	10.0
**23b**		CH(CH_3_)_2_	1.30	0.650
**Donepezil ^d^**			0.021	2.25
**Galantamine ^d^**			0.55	12.0

^a^ K_i_ values determined by applying the Cheng–Prusoff equation from IC_50_ values, by regression (GraphPad Prism software ver. 5.01), or inhibition percentage at 50 μM. Data are the means of three independent measurements, each one performed in triplicate (SEM < 5%). ^b^ Electric eel acetylcholinesterase and horse serum butyrylcholinesterase were tested as the target enzymes. ^c^C2 gem-dimethyl analogue of compound **19h** (see structure **19l** in Table 3). ^d^ Donepezil and galantamine were used as positive controls against cholinesterases.

## Data Availability

All data presented in this study are available in the article and in Supplementary Information.

## Supplementary Materials

The following supporting information can be downloaded at: https://www.mdpi.com/article/10.3390/molecules27134269/s1, Spectroscopic data and ^1^H NMR spectra of the newly synthesized compounds; Table S1.

## Sample Availability

Samples of the compounds presented are available from the authors.
