# Supplementary material for: Assessing the Role of a Malonamide Linker in the Design of Potent Dual Inhibitors of Factor Xa and Cholinesterases
Source: Molecules. 2022 Jul 2;27(13):4269. doi: 10.3390/molecules27134269 (PMC9268553; doi:10.3390/molecules27134269)

Assessing the Role of Malonamide Linker in the Design of Po-tent Dual Inhibitors of Factor Xa and Cholinesterases

Rosa Purgatorio,[1†] Nicola Gambacorta,[1†] Francesco Samarelli,[1] Gianfranco Lopopolo,[1#] Modesto de Candia,[1*] Marco Catto,[1] Orazio Nicolotti,[1] and Cosimo D. Altomare.[1]

[1]Department of Pharmacy—Pharmaceutical Sciences, University of Bari Aldo Moro, Via Orabona 4, 70125 Bari, Italy; rosa.purgatorio@uniba.it (R.P.); nicola.gambacorta1@uniba.it (N.G.); francesco.samarelli@uniba.it (F.S.); marco.catto@uniba.it (M.C.); orazio.nicolotti@uniba.it (O.N.); cosimodamiano.altomare@uniba.it (C.D.A.)
[#]Present address (G.L.) gianfranco.lopopolo@newchemspa.it; Research & Development Dept., Newchem SpA, via Roveggia 47, 37138 Verona
* Correspondence: modesto.decandia@uniba.it
† These authors contributed equally to this work.

Supplementary information

1. **Synthesis and spectroscopic data** **pag. 2**
2. **Inhibiton potency.** **pag. 20**
3. **1H NMR spectra of selected compounds** **pag. 21**

1. Synthesis and spectroscopic data

Synthesis of compounds 2a-c.

Compounds **2a** and **2b** have been prepared by following the general procedure described in the paper manuscript.

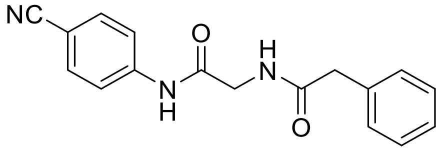

4-cyano-N-[3-(phenyl-4-yl)-2-oxopropyl]benzamide (**2b**). Colorless oil, yield 23% (350 mg). IR cm^{-1}: 3274, 2228, 1683, 1649, 1603. ^{1}H NMR (300 MHz, DMSO-d_6), δ_H: 9.79 (s, 1H), 7.58 (d, *J* = 8.8 Hz, 2H), 7.47 (d, *J* = 8.8 Hz, 2H), 7.30 – 7.19 (m, 5H), 6.92 (m, 1H), 3.99 (d, *J* = 5.2 Hz, 2H), 3.56 (s, 2H).

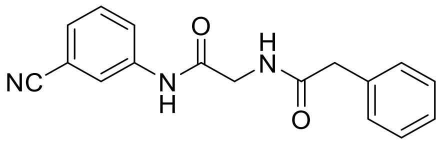

3-cyano-N-[3-(phenyl-4-yl)-2-oxopropyl]benzamide (**2b**). Brown oil, yield 45% (100 mg). IR cm^{-1}: 3290, 2222, 1650, 1100, 704. 1H NMR (300 MHz, $CDCl_3$), δ_H 8.12 (t, J = 1.7 Hz, 1H), 7.99 (s, 1H), 7.90 (d, J = 7.7 Hz, 1H), 7.81 (d, J = 2.0 Hz, 1H), 7.45 (7, J = 7.5 Hz, 1H), 7.24 (d, J = 7.7 Hz, 1H), 7.20 – 7.10 (m, 3H), 6.98 – 6.87 (m, 2H) 4.51 (d, J = 5.8 Hz, 2H), 4.20 (d, J = 5.0 Hz, 2H).

Synthesis of compounds 3a-c.

The synthesis of compound **3c** has been reported as an example in the paper manuscript. Compounds **3a** and **3b** have been prepared by following the same reported procedure.

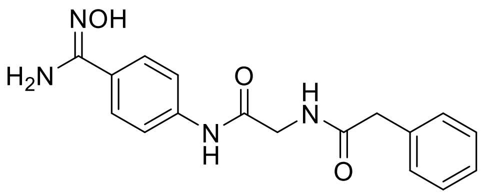

N-[4-(hydroxyamidino)phenyl]-2-(phenylacetamido) acetamide (**4a**). White microcrystalline solid, yield 84% (400 mg). IR cm^{-1}: 3398, 3329, 1690, 1643, 1530, 833. ^{1}H NMR (300 MHz, DMSO-d_6), δ_H: 10.06 (s, 1H), 9.50 (s, 1H), 8.38 (t, *J* = 5.8 Hz, 1H), 7.59 (d, *J* = 9 Hz, 2H), 7.54 (d, *J* = 9 Hz, 2H), 7.27 (s, 2H), 5.72 (s, 2H), 3.89 (d, *J* = 5.8 Hz, 2H), 3.50 (s, 2H).

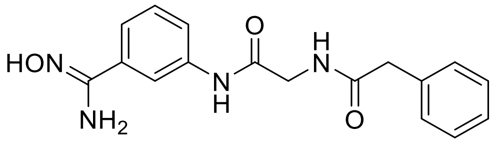

N-{[3-(N-hydroxycarbamimidoil)-phenylcarbamoil]-methyl}-2-phenylacetamide (**3b**). White-grey amorphous powder, yield 51% (255 mg). IR cm^{-1}: 3444.8, 3400.5, 1678.5, 1646.8. ^{1}H-NMR (DMSO-d_6) δ_H: 10.04 (s, 1H), 9.66 (s, 1H), 8.38 (t, J = 8.0 Hz, 1H), 7.85 (s, 1H), 7.60 (s, 1H), 7.30-7.19 (m, 7H), 5.82 (s, 1H), 3.87 (d, J = 5.5 Hz 2H), 3.59 (s, 2H).

Synthesis of compounds 4a-c.

The synthesis of compound **4c** has been reported as an example in the paper manuscript. Compounds 4**a** and **4b** have been prepared by following the same reported procedure.

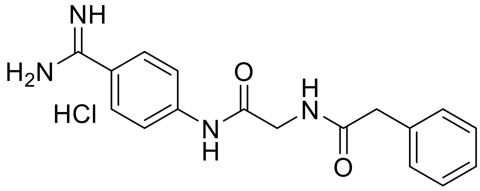

N-(4-amidinophenyl)-2-(phenylacetamido) acetamide hydrochloride (**4a**). White solid, yield 68% (290 mg), M.p. 235-238 °C. IR cm^{-1}: 3375, 3318, 3239, 1685, 1663, 1525, 845, 737. ^{1}H NMR (300 MHz, DMSO-d_6), δ_H: 10.58(s, 1H), 9.20 (s, 2H), 8.97 (s, 2H), 8.47 (t, J = 5.8 Hz, 1H), 7.78 (s, 4H), 7.27 (s, 5H), 3.93 (d, J = 5.8 Hz, 2H), 3.50 (s, 2H). HRMS [ESI], m/z: 311.1500 $[M+H]^+$ for $C_{17}H_{19}N_4O_2^+$. Anal. Calc. for $C_{17}H_{19}N_4O_2Cl$, %: C, 58.87; H, 5.52; N, 16.15; found, %: C, 58.98; H, 5.62; N, 16.22.

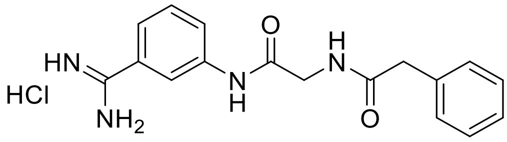

N-[(3-carbamimidoil-phenyl carbamoil)-methyl]-2-phenyl-acetamide (**4b**). White solid, yield 63% (432 mg), m.p. 269-270 °C. IR cm^{-1}: 3434.7, 3255.9, 1701, 1687.3. ^{1}H-NMR (d6-DMSO) δ: 10.48 (s, 1H), 9.35 (s, 2H), 9.10 (s, 2H), 8.48 (s, 1H), 8.07 (s, 1H), 7.80 (d, J = 9 Hz, 1H), 7.25 (t, J = 6 Hz, 1H), 7.44 (d, J = 6 Hz, 1H), 7.2 (m, 5H), 3.91(d, J = 5.6 Hz, 2H), 3.5 (s, 2H). HRMS [ESI], m/z: 311.1511 $[M+H]^+$ for $C_{17}H_{19}N_4O_2^+$. Anal. Calc. for $C_{17}H_{19}N_4O_2Cl$, %: C, 58.87; H, 5.52; N, 16.15; found, %: C, 59.00; H, 5.66; N, 16.12.

Synthesis of compounds 6a-e, 7a-l, 8a-b, 9a-b, 10a-c, 11a-b.

The synthesis of compound **7h** has been reported as an example in the paper manuscript. Compounds **6a-e, 7a-l, 8a-b, 9a-b, 10a-c, 11a-b** have been prepared by following the same reported procedure.

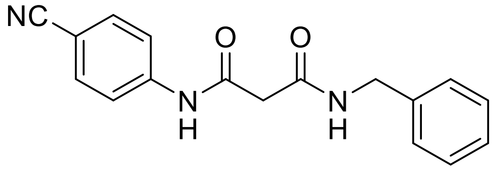

N^1-benzyl-N^3-(4-cyanophenyl)malonamide (**6a**). Brown oil, yield 52% (440 mg). IR cm^{-1}: 3270, 2221, 1670, 1632, 711. ^{1}H NMR (300 MHz, DMSO-d_6), δ_H: 10.3o (s, 1H), 8.01 (s, 1H), 7.57 (d, *J* = 9.0 Hz, 2H), 7.42 (d, *J* = 9.0 Hz, 2H), 7.20-7.11 (m, 3H), 7.05 (d, *J* = 7.5 Hz, 2H), 4.25 (d, *J* = 4.0 Hz, 2H), 3.29 (s, 2H).

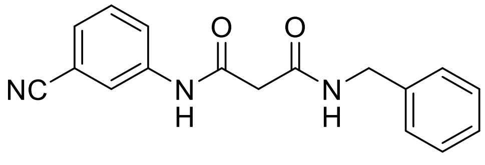

N^1-benzyl-N^3-(3-cyanophenyl)malonamide (**6b**). Brown oil, yield 22% (140 mg). IR cm^{-1}: 3268, 2222, 1672, 1635, 702. ^{1}H NMR (300 MHz, DMSO-d_6), δ_H: 10.45 (s, 1H), 8.65 (t, *J* = 5.5 Hz, 1H), 8.10 (s, 1H), 7.80 – 7.50 (m, 3H), 7.50 (d, *J* = 8.5 Hz, 2H), 7.22-7.10 (m, 3H), 4.29 (d, *J* = 5.0 Hz, 2H), 3.27 (s, 2H).

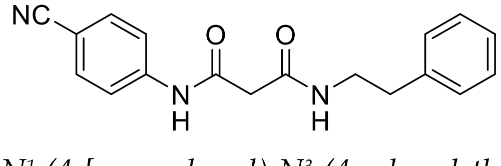

N^1-(4-[cyanophenyl)-N^3-(4- phenylethyl)malonamide (**6c**). White solid, yield 74% (555 mg). IR cm^{-1}: 3296, 2230, 1671, 1643, 1600, 1545, 836, 698. ^{1}H NMR (300 MHz, DMSO-d_6), δ_H: 10.19 (s, 1H), 7.70 (d, *J* = 8.8 Hz, 2H), 7.34 – 7.19 (m, 5H), 6.41 (m, 1H), 3.62 – 3.55 (m, 2H), 3.28 (s, 2H), 2.85 (t, *J* = 6.9 Hz, 2H).

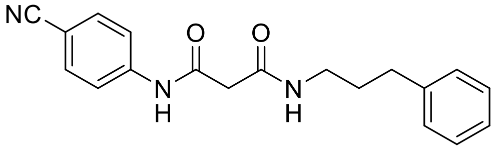

N^1-(4-[cyanophenyl)- N^3-(4-phenylpropyl)malonamide (**6d**). White solid, yield 67% (1.05 g). IR cm^{-1}: 3289, 2226, 1671, 1632, 1599, 1536, 696. ^{1}H NMR (300 MHz, DMSO-d_6), δ_H: 10.13 (s, 1H),

7.71 (d, *J* = 8.7 Hz, 2H), 7.60 (d, J = 8.7 Hz, 2H), 7.31 – 7.16 (m, 5H), 6.28 (m, 1H), 3.34 (q, *J* = 6.5 Hz, 2H), 3.30 (s, 2H), 2.68 (t, *J* = 7.5 Hz, 2H), 1.90 (quintet, *J* = 7.4 Hz, 2H).

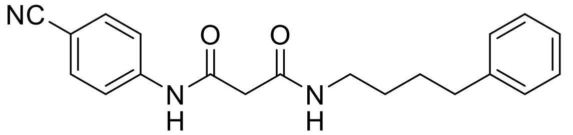

N^1-(4-[cyanophenyl)-N^3-[4-(phenyl)butyl] malonamide (**6e**). White solid, yield 49% (800 mg). IR cm^{-1}: 3442, 3311, 2229, 1673, 1638, 1603, 1543, 832, 700. ^{1}H NMR (300 MHz, DMSO-d_6), δ_H: 10.20 (s, 1H), 7.70 (d, *J* = 8.7 Hz, 2H), 7.59 (d, J = 8.7 Hz, 2H), 7.29 – 7.14 (m, 5H), 6.48 (s, 1H), 3.33 – 3.30 (m, 4H), 2.26 (t, *J* = 7.1 Hz, 2H), 1.71 – 1.54 (m, 4H).

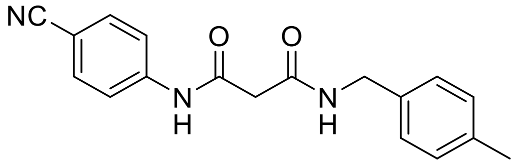

N^1-(4-[cyanophenyl)-N^3-(4-methylbenzyl)malonamide (**7a**). White solid, yield 48% (425 mg). IR cm^{-1}: 3274, 2226, 1672, 1635, 835, 811. ^{1}H NMR (300 MHz, DMSO-d_6), δ_H: 10.32 (s, 1H), 8.02 (s, 1H), 7.58 (d, *J* = 8.9 Hz, 2H), 7.41 (d, *J* = 8.9 Hz, 2H), 7.02 (d, *J* = 6.5 Hz, 2H), 6.96 (d, *J* = 6.5 Hz, 2H), 4.23 (d, *J* = 3.3 Hz, 2H), 3.25 (s, 2H), 2.16 (s, 3H).

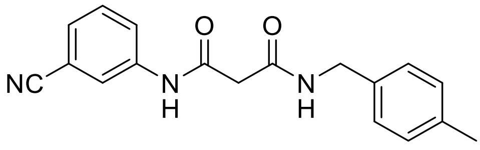

N^1-(3-cyanophenyl)-*N^3*-(4-methyl-benzyl)-malonamide (**7b**). Pale brown solid, yield 40% (300 mg). IR cm^{-1}: 3435, 3297, 2232, 1675, 1650. ^{1}H-NMR (300 MHz, $CDCl_3$) δ: 10.01 (s, 1H), 8.05 (s, 1H), 7.71-7.67 (m, 1H), 7.44-7.39 (m, 2H), 7.25 (s, 1H), 7.16 (s, 3H), 6.5 (s, 1H), 4.43 (d, *J* = 3 Hz, 2H), 3.58 (s, 2H), 2.36 (s, 3H).

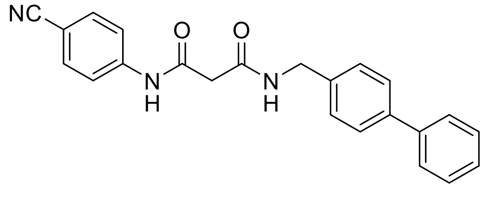

N^1-[4-(cyano)-phenyl]-N^3-biphenylmethylenmalonamide (**7c**). White solid, yield 68% (450 mg). IR cm^{-1}: 3269, 2221, 1662, 1630, 830, 810. ^{1}H NMR (300 MHz, DMSO-d_6), δ_H: 10.32 (s, 1H), 8.02

(s, 1H), 7.60 (d, *J* = 8.5 Hz, 2H), 7.45 (d, *J* = 8.5 Hz, 2H), 7.40-7.35 (m, 4H), 7.25-7.10 (m, 5H), 4.25 (d, *J* = 4.5 Hz, 2H), 3.22 (s, 2H).

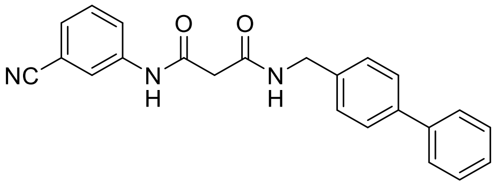

N[1]*-[3-(cyano)-phenyl]-N*[3]*-biphenylmethylenmalonamide* (**7d**). Pale brown oil, yield 30% (150 mg). IR cm^{-1}: 3446, 3284, 2226, 1674, 1632, 1601, 1538, 761, 690. ^{1}H NMR (300 MHz, $CDCl_3$), δ_H: 10.13 (s, 1H), 7.71 (d, J = 8.8 Hz, 2H), 7.65 – 7.32 (m, 11H), 6.57 (br s, 1H), 4.52 (d, J = 5.8 Hz, 2H), 3.4 (s, 2H).

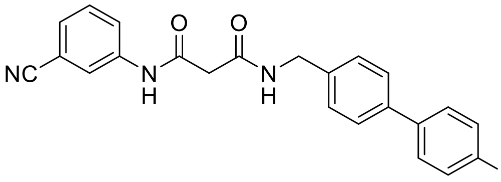

N[1]*-(3-cyanophenyl)-N*[3]*-((4′-fluoro-[1,1′-biphenyl]-4-yl)methyl)malonamide* (**7g**). Brown oil, yield 14% (192 mg). IR cm^{-1}: 3282, 2221, 1672, 1635, 1600, 690. ^{1}H NMR (300 MHz, DMSO-d6), δ_H: 10.49 (s, 1H), 8.63 (t, *J* = 5.8 Hz, 1H), 8.09 (s, 1H), 7.80 – 7.50 (m, 7H), 7.38 – 7.23 (m, 4H), 4.33 (d, *J* = 5.8 Hz, 2H), 3.31 (s, 2H).

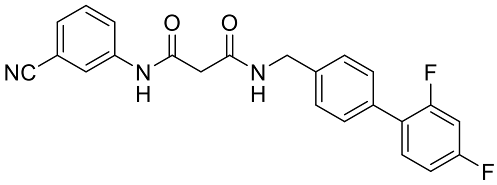

N[1]-(3-cyanophenyl)-*N*[3]-(2′,4′-difluoro)biphenylmethylen malonammide (**7h**). Yellow oil, yield 27% (70 mg). IR cm^{-1}: 3321, 2231, 1681, 1651, 1431, 964, 795. ^{1}H NMR (500 MHz, $CDCl_3$), δ_H: 10.09 (s, 1H), 8.04 (s, 1H), 7.74 – 7.61 (m, 1H), 7.50 – 7.28 (m, 8H), 6.96 – 6.83 (m, 2H), 4.51 (d, J = 5.8 Hz, 2H), 3.45 (s, 2H).

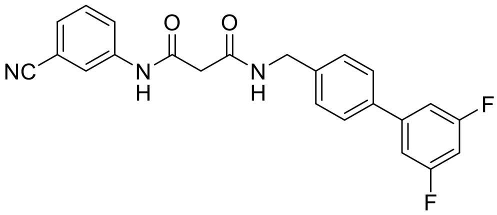

N^1-[3-(cyano)phenyl]-*N^3*-(3′,5′-difluoro) biphenylmethylenmalonamide (**7i**). Brown oil, yield 62% (635 mg). IR cm^{-1}: 3327, 2929, 2226, 1681, 1625, 1485, 1116, 986, 792, 682. ^1H NMR (300 MHz, DMSO-d_6), δ_H: 10.48 (s, 1H), 8.66 (t, J = 6 Hz, 1H), 8.1 (s, 1H), 7.82 – 7.75 (m, 2H), 7.70 (d, J = 8.3 Hz, 2H), 7.65 – 7.35 (m, 5H), 7.23 – 7.15 (m, 1H), 4.35 (d, J = 5.9 Hz, 2H), 3.32 (s, 2H).

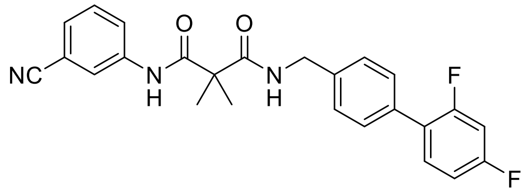

N^1-(3-cyanophenyl)-*N^3*-((2',4'-difluoro-[1,1'-biphenyl]-4-yl)methyl)-2,2-dimethylmalonamide (**7l**). Brown oil, yield 67% (870 mg). IR cm^{-1}: 3345, 3285, 3074, 2233, 1652, 1604, 1527, 1494, 964, 848, 800, 683. ^1H NMR (300 MHz, $CDCl_3$), δ_H: 9.73 (s, 1H), 8.05 (s, 1H), 7.65 (d, *J* = 7.4 Hz, 1H), 7.49 – 7.31 (m, 6H), 6.99 – 6.88 (m, 2H), 6.52 (br s, 1H), 4.53 (d, *J* = 5.5 Hz, 2H), 1.60 (s, 6H).

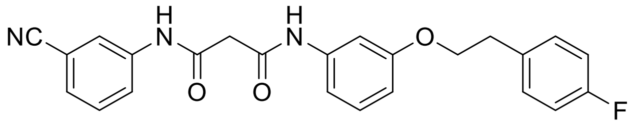

N^1-(3-cyanophenyl)-N^3-(3-(4-fluorophenethoxy)phenyl)malonamide (**8a**). Orange oil, yield 30%. IR cm^{-1}: 3326, 2928, 2232, 1663, 1609, 1594, 1501, 1431, 1220, 1157, 1041, 830, 792, 683. ^1H-NMR ($CDCl_3$): δ 9.71 (s, 1H), 8.76 (s, 1H), 8.06 (d, 1H), 7.72-7.69 (m, 1H), 7.42-7.40 (m, 2H), 7.25-7.19 (m,4H), 7.04-6.95 (m, 3H), 6.71-6.67

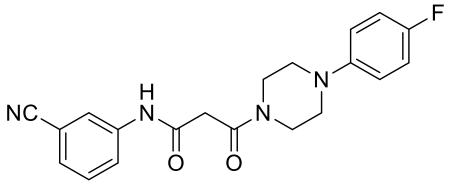

N-[4-(cyano)phenyl]-3-[4-(4-fluorophenyl)piperazin-1-yl] propanamide (**9a**). Pale yellow solid. M.P.= 146-148°C. IR cm^{-1}: 3426, 2226, 1693, 1619, 1557, 1511, 1236, 822, 703, 661. ^1H NMR (300 MHz, $CDCl_3$), δ_H: 10.49 (s, 1H), 8.06 (d, J = 1.1 Hz, 1H), 7.71 (d, J = 7.1 Hz, 1H), 7.46 – 7.36 (2H, m), 7.00 (d, J = 9.07, 1H), 6.97 (d, J = 9.07 Hz, 1H), 6.90 (d, J = 9.13 Hz, 1H), 6.88 (d, J = 9.13 Hz, 1H), 3.84 (t, J = 5.09 Hz, 2H), 3.75 (t, J = 5.09 Hz, 2H), 3.55 (s, 2H), 3.18 – 3.09 (m, 4H).

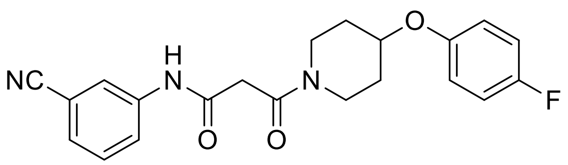

N-(3-cyanophenyl)-3-(4-(4-fluorophenoxy)piperidin-1-yl)-3-oxopropanamide (**10a**). Brown oil, yield 31% (250 mg). IR cm^{-1}: 3331, 2861, 2238, 1702, 1639, 1500, 1428, 1225, 1053, 830, 762. ^{1}H NMR (300 MHz, DMSO-d6), δ_H: 10.45 (s, 1 H), 8.08 (s, 1 H), 7.76 (m, 1 H), 7.52 (m, 2 H), 7.09 (m, 2 H), 6.99 (m, 2 H), 4.55 (m, 1 H), 3.85 (m, 1 H), 3.71 (m, 1 H), 3.56 (s, 2 H), 3.32 (m, 2 H), 1.90 (m, 2 H), 1.63 (m, 1 H), 1.50 (m, 1 H).

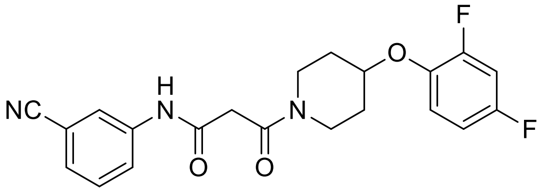

N-(3-cyanophenyl)-3-(4-(2,4-difluorophenoxy)piperidin-1-yl)-3-oxopropanamide (**10b**). Dark brown oil, yield 60% (715 mg). IR cm^{-1}: 3335, 1700, 1634, 831, 760. ^{1}H NMR (300 MHz, CDCl3), δ_H: 10.60 (s, 1H), 8.05 (s, 1H), 7.73 – 7.65 (m, 1H), 7.46 – 7.35 (m, 2H), 7.02 – 6.77 (m, 3H), 4.46 (quintet, *J* = 4.4 Hz, 1H), 3.90 – 3.67 (m, 4H), 3.50 (s, 2H), 1.95 – 1.85 (m, 4H).

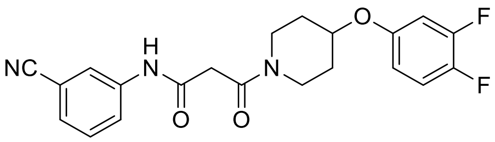

N-(3-cyanophenyl)-3-(4-(3,4-difluorophenoxy)piperidin-1-yl)-3-oxopropanamide (**10c**). Dark brown oil, yield 35% (590 mg). IR cm^{-1}: 3328, 1675, 1631, 831, 760. ^{1}H NMR (300 MHz, CDCl3), δ_H: 10.55 (s, 1H), 8.05 (s, 1H), 7.70 (dt, *J* = 7.1, 1.1 Hz, 1H), 7.48 – 7.36 (m, 2H), 7.06 (q, *J* = 9.4 Hz, 1H), 6.79 – 6.70 (m, 1H), 6.65 – 6-58 (m, 1H), 4.50 (quintet, *J* = 4.4 Hz, 1H), 3.90 – 3.67 (m, 4H), 3.50 (s, 2H), 1.95 – 1.85 (m, 4H).

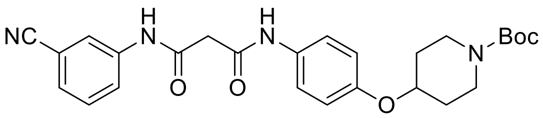

tert-butyl-4-[4-({3-[(3-cyanophenyl)amino]-3-oxopropanoylamino)phenoxy]piperidine-1 carboxylate (**11a**). Pale brown amorphous solid, yield 75% (950mg). IR cm^{-1}: 3311, 2331, 1693, 1239, 1173, 1030, 867, 831, 794, 684. ^{1}H NMR (300 MHz, $CDCl_3$), δ_H: 9.80 (s, 1H), 8.58 (s, 1H), 8.07 (d, *J* = 1 Hz, 1H) 7.79-7.69 (m, 1H), 7.45 – 7.26 (m, 4H), 6.90 (d, *J* = 9 Hz, 2H), 4.45 – 4.39

(m, 1H), 3.73 – 3.64 (m, 2H), 3.57 (s, 2H), 3.40-3.25 (m, 2H), 1.94-1.85 (m, 2H), 1.80 - 1.65 (m, 2H), 1.47 (s, 9H).).

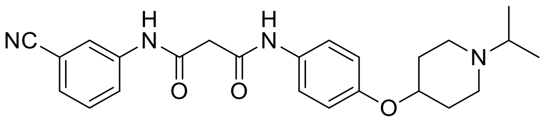

N[1]*-{3-(cyano)phenyl}-N*[3]*-[4-(1-isopropylpiperidin-4-iloxy)phenyl]malonamide* (**11b**). Dark brown oil, yield 42% (490 mg). IR cm^{-1}: 3327, 2238, 1678, 1639, 1225, 1053, 760.^{1}H NMR (300 MHz, $CDCl_3$), δ_H: 9,83 (s, 1H), 8,46 (s, 1H), 7,42 (m, 2H), 7,25 (m, 4H), 6,96 (m, 2H), 4,18 (m, 1H), 3,46 (m, 2H), 2,82 (m, 4H), 2,47 (m, 4H), 2,16 (d, 6H), 1,09 (m, 1H). ESI-MS: *m/z:* 421.0 $[M+H]^+$.

Synthesis of compounds 12a-e, 13a-l, 14a-b, 15a-b, 16a-c, 17a-b.

The synthesis of compound **13h** has been reported as an example in the paper manuscript. Compounds **12a-e, 13a-l, 14a-b, 15a-b, 16a-c, 17a-b** have been prepared by following the same reported procedure.

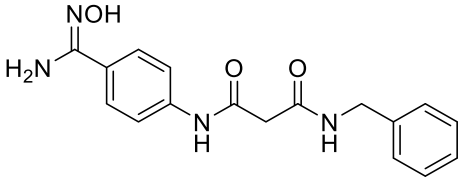

N[1]*-{4-[amino(hydroxyimino)methyl]phenyl}-N*[3]*-(4-phenylethyl)malonamide* (**12a**). Amorphous white powder, yield 42% (311mg). IR cm^{-1}: 3410, 3322, 1683 ^{1}H NMR (300 MHz, DMSO-d_6), δ_H: 10.18 (s, 1H), 9.52 (s, 1H), 7.59 (d, *J* = 9 Hz, 2H), 7.48 (d, *J* = 9 Hz, 2H), 7.28 – 7.15 (m, 5H), 5.65 (d, *J* = 5.5 Hz, 2H), 3.33 (s, 2H).

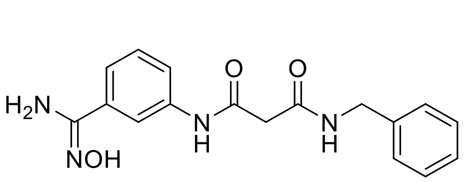

N[1]*-{4-[amino(hydroxyimino)methyl]phenyl}-N*[3]*-(4-phenylethyl)malonamide* (**12b**). Amorphous white powder, yield 66% (365 mg). IR cm^{-1}: 3429, 3390, 3318, 1645, 801 ^{1}H NMR (300 MHz, DMSO-d_6) δ: 10.15 (s, 1H), 9.60 (s, 1H), 8.29 (s, 1H), 7.87 (s, 1H), 7.62-7.60 (m, 1H), 7.30-7.28 (m, 2H), 7.25-7.09 (m, 4H), 5.73 (s, 2H), 4.24 (d, *J* = 5.3 Hz, 2H), 3.28 (s, 2H).

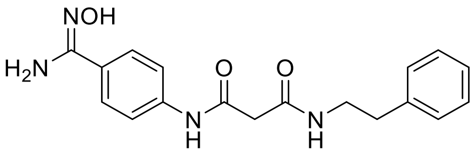

N^1-{4-[amino(hydroxyimino)methyl]phenyl}-N^3-(4-phenylethyl)malonamide (**12c**). Amorphous white powder, yield 82% (911mg). IR cm^{-1}: 3416, 3324, 1683, 1635, 1527, 841, 698. ^{1}H NMR (300 MHz, DMSO-d_6), δ_H: 10.17 (s, 1H), 9.53 (s, 1H), 8.16 (t, *J* = 5.3 Hz, 1H), 7.60 (d, *J* = 9 Hz, 2H), 7.56 (d, *J* = 9 Hz, 2H), 7.29 – 7.16 (m, 5H), 5.77 (s, 2H), 3.28 (t, *J* = 7.7 Hz, 2H), 3.22 (s, 2H), 2.71 (t, *J* = 7.7 Hz, 2H).

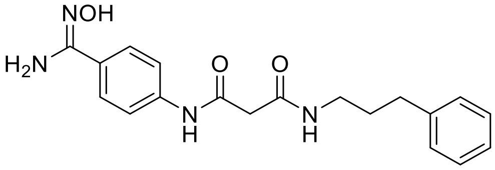

N^1-{4-[amino(hydroxyimino)methyl]phenyl}-N^3-(4-phenylpropyl)malonamide (**12d**). Amorphous white powder, yield 86% (862mg). IR cm^{-1}: 3330, 1662, 1634, 1543, 842, 697. ^{1}H NMR (300 MHz, DMSO-d_6), δ_H: 10.18 (s, 1H), 9.52 (s, 1H), 8.10 (t, *J* = 5.5 Hz, 1H), 7.59 (d, *J* = 9 Hz, 2H), 7.55 (d, *J* = 9 Hz, 2H), 7.28 – 7.15 (m, 5H), 5.74 (s, 2H), 3.23 (s, 2H), 3.11 – 3.04 (m, 2H), 2.59 (t, *J* = 7.5 Hz, 2H), 1.70 (m, *J* = 7.5 Hz, 2H).

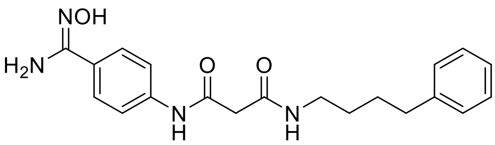

N^1-{4-[amino(hydroxyimino)methyl]phenyl}- N^3-[4-(phenyl)butyl] malonamide (**12e**). Amorphous white powder, yield 58% (446mg). IR cm^{-1}: 3425, 3354, 1682, 1635, 1532, 700. ^{1}H NMR (300 MHz, DMSO-d_6), δ_H: 10.18 (s, 1H), 9.56 (s, 1H), 8.05 (t, *J* = 5.2 Hz, 1H), 7.59 (d, *J* = 9.1 Hz, 2H), 7.54 (d, *J* = 9.1 Hz, 2H), 7.27 – 7.14 (m, 5H), 5.81 (s, 2H), 3.20 (s, 2H), 3.08 (q, *J* = 6 Hz, 2H), 2.55 (t, *J* = 7.4 Hz, 2H), 1.56 (quintet, *J* = 7.4 Hz, 2H), 1.41 (quintet, J = 7.4 Hz, 2H).

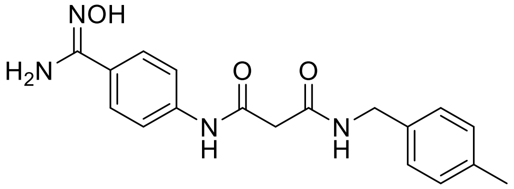

N^1-{4-[amino(hydroxyimino)methyl]phenyl}-N^3-(4-methylbenzyl)malonamide (**13a**). Amorphous white powder, yield 68% (450mg). IR cm^{-1}: 3282, 1634, 1532, 837. ^{1}H NMR (300 MHz, DMSO-d_6), δ_H: 10.19 (s, 1H), 9.52 (s, 1H), 8.51 (m, 1H), 7.59 (d, *J* = 8.9 Hz, 2H), 7.55 (d,

J = 9 Hz, 2H), 7.16 (d, *J* = 8.1 Hz, 2H), 7.11 (d, *J* = 8.1 Hz, 2H), 5.72 (s, 2H), 4.24 (d, *J* = 5.8 Hz, 2H), 3.32 (s, 2H).

13b.

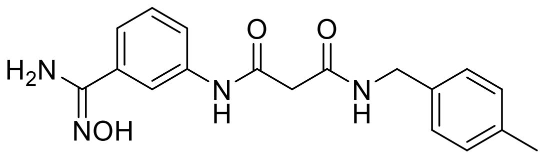

N¹-(3-hydroxycarbamimidoylphenyl)-N³-(4-methyl-benzyl)-malonamide (**13b**). Amorphous white powder, yield 66% (730 mg). IR cm^{-1} : 3428, 3389, 3319, 1645, 1609, 800. ^{1}H NMR (300 MHz, d6-DMSO) δ: 10.15 (s, 1H), 9.60 (s, 1H), 8.29 (s, 1H), 7.87 (s, 1H), 7.62-7.60 (m, 1H), 7.30-7.28 (m, 2H), 7.25-7.09 (m, 4H), 5.73 (s, 2H), 4.24 (d, *J* = 5.5 Hz,2H), 3.28 (s, 2H), 2.06 (s, 3H).

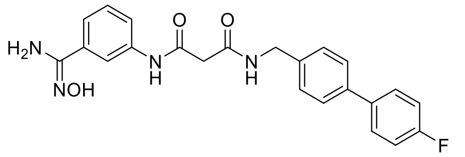

N¹-((4′-fluoro-[1,1′-biphenyl]-4-yl)methyl)-N³-(3-(N′-hydroxycarbamimidoyl)phenyl)malonamide (**13g**). Amorphous white powder, yield 64% (134 mg). IR cm^{-1} : 3428, 3389, 3319, 1645, 1609, 800. ^{1}H NMR (300 MHz, DMSO-d6), δ_H: 10.25 (s, 1H), 9.64 (s, 1H), 8.61 (t, *J* = 5.8 Hz, 1H), 7.88 (s, 1H), 7.7 – 7.56 (m, 5H), 7.38 – 7.21 (m, 6H), 5.80 (br s, 2H), 4.33 (d, *J* = 5.8 Hz, 2H), 3.31 (s, 2H).

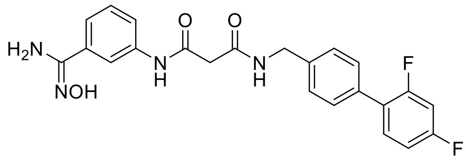

N¹-((2′,4′-difluoro-[1,1′-biphenyl]-4-yl)methyl)-N³-(3-(N′-hydroxycarbamimidoyl)phenyl)malonamide (**13h**). Amorphous white powder,yield 73% (67 mg). IR cm^{-1}: 3258, 2928, 2850, 1637, 1591, 1561, 1494, 1448, 1141, 1100, 806, 789; ^{1}H NMR (300 MHz, DMSO-d6), δ_H: 10.18 (s, 1H), 9.60 (s, 1H), 8.63 (t, *J* = 6.0 Hz, 1H), 7.88 (s, 1H), 7.66 – 7.25 (m, 9H), 7.17 (td, *J* = 8.5, 2.6 Hz, 1H), 5.73 (s, 2H), 4.35 (d, *J* = 5.9 Hz, 2H), 3.32 (s, 2H).

13i.

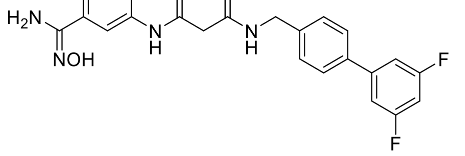

N¹-{3-[amino(hydroxyimino)methyl]phenyl}-N³-(3′,5′-difluoro) biphenylmethylenmalonamide (**13i**). Amorphous white powder, yield 90% (582 mg). IR cm^{-1}: 3250, 2852, 1637, 805; ^{1}H NMR (300 MHz, DMSO-d$_6$), δ_H: 10.18 (s, 1H), 9.60 (s, 1H), 8.66 (t, J = 6.0 Hz, 1H), 7.88 (s, 1H), 7.66 – 7.18 (m, 10H), 5.73 (s, 2H), 4.34 (d, J = 5.8 Hz, 2H), 3.32 (s, 2H).

15a.

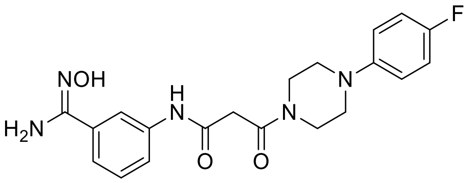

3-(4-(4-fluorophenyl)piperazin-1-yl)-N-(4-(N′-hydroxycarbamimidoyl)phenyl)-3-oxopropanamide (**15a**). Amorphous white powder, yield 81% (920 mg). IR cm^{-1}: 3407.1, 1675.7, 1627.7, 1529.7, 1510.4, 1227.9, 828.7, 667.8. ^{1}H NMR (300 MHz, DMSO-d6), δ_H: 10.20 (s, 1H), 9.51 (s, 1H), 7.59 (d, J = 8.8 Hz, 2H), 7.54 (d, J = 8.8 Hz, 2H), 7.08 – 6.97 (m, 4H), 5.72 (s, 2H), 3.61 – 3.56 (m, 4H), 3.41 (t, J = 6.23, 2H), 3.10 – 3.03 (m, 4H).

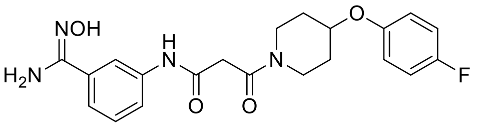

3-(4-(4-fluorophenoxy)piperidin-1-yl)-N-(3-(N′-hydroxycarbamimidoyl)phenyl)-3-oxopropanamide (**16a**). Amorphous white powder, yield 79% (320 mg). IR cm^{-1}: 3256, 2928, 1635, 1591, 1562, 1494, 1447, 1142, 963. ^{1}H NMR (300 MHz, DMSO-d$_6$), δ_H: 10.18 (s, 1H), 8.63 (br s, 1H), 7.92 (s, 1H), 7.62 – 7.54 (m, 1H), 7.35 – 7. 23 (m, 2H), 7.15 – 6.95 (m, 4H), 5.73 (s, 2H), 4.55 (quintet, *J* = 3.8 Hz, 1H), 3.88 -3.68 (m, 2H), 3.32 (s, 2H), 3.45 – 3.23 (m, 2H), 1.98 – 1.82 (m, 2H), 1.7 – 1.4 (m, 2H).

17a.

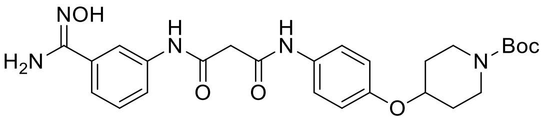

t-butyl-4-(4-{[3-({3-[amino(hydroxyimino)methyl]phenyl}amino)-3-oxopropanoyl]amino}phenoxy)piperidine-1-carboxylate (**17a**). Amorphous white powder, yield 79% (930 mg). IR cm^{-1}: 3310, 1668, 1546, 1508, 1428, 1237, 1109, 1031, 943, 831, 795. ^{1}H NMR (300 MHz, $CDCl_3$), δ_H: 9.76 (s, 1H), 9.47 (s, 1H), 7.63 (s, 1H), 7.50-7.23 (m, 4H), 7.19-7.06 (m, 2H), 6.69 (d, *J* = 8.5 Hz, 2H), 5.20 (s, 2H), 4.26 (m, 1H), 3.70 (s, 2H), 3.68-3.50 (m, 2H), 3.44-3.20 (m, 2H), 1.86-1.79 (m, 2H), 1.65-1.51 (m, 2H), 1.44 (s, 9H).

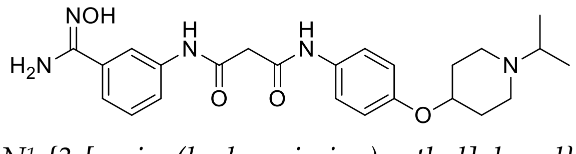

N^1-{3-[amino(hydroxyimino)methyl]phenyl}-N^3-[4-(1-isopropylpiperidin-4-iloxy)phenyl]malonamide (**17b**). Amorphous white powder, yield 97% (512 mg). ^{1}H NMR (300 MHz, DMSO-d_6), δ_H: 10,24 (s, 1H), 9,61 (t, J = 4,6 Hz, 1H), 7,56 (m, 2H), 7,39 (m, 2H), 6,68 (m, 2H), 6,47 (m, 2H), 5,73 (s, 2H), 4,34 (m, 2H), 4,07 (m, 1H), 3,32 (m, 8H), 2,48 (s ,6H).

Synthesis of compounds 18a-e, 19a-l, 20a-b, 21a-b, 22a-c, 23a-b.

The synthesis of compound **19h** has been reported as an example in the paper manuscript. The amidine compounds have been prepared by following the same reported procedure.

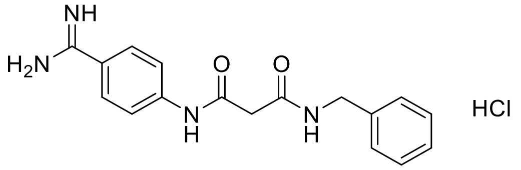

N^1-{4-[amino(imino)methyl]phenyl}-N^3-(benzyl)malonamide hydrochloride (**18a**). Pale yellow solid, yield 61% (290mg), M.p. 242 – 245 °C. IR cm^{-1}: 3363, 3240, 165, 1627.1, 1600, 843, 735. ^{1}H NMR (300 MHz, DMSO-d_6), δ_H: 10.78 (s, 1H), 9.25 (s, 2H), 8.99 (s, 2H), 8.64 (t, *J* = 5.5 Hz, 1H), 7.80 (s, 4H), 7.16 (d, *J* = 8.1 Hz, 2H), 7.10-6.95 (m, 3H), 4.25 (d, *J* = 6.0 Hz, 2H), 3.37 (s, 2H). HRMS [ESI], m/z: 311.1504 $[M+H]^+$ for $C_{17}H_{19}N_4O_2$. Anal. Calc. for $C_{17}H_{19}N_4O_2Cl$, %: C, 58.87; H, 5.52; N, 16.15; found, %: C, 59.01; H, 5.54; N, 16.42.

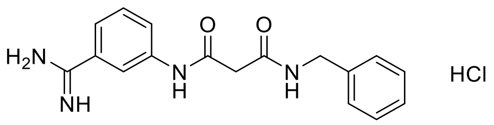

N^1-(3-carbamimidoil-phenyl)-N^3-(benzyl) malonamide hydrochloride (**18b**) –White solid, yield 27% (150 mg) m.p. = 166-168 °C. IR cm^{-1} : 3292, 1680, 1625, 802; ^{1}H-NMR (300 MHz, DMSO-d6) δ : 10.64 (s, 1H), 9.32 (s, 2H), 9.10 (s, 2H), 8.63 (t, *J* = 5.5 Hz, 1H), 8.09 (s, 1H), 7.80 (d, *J* = 8.5 Hz, 1H), 7.53 (t, *J* = 8.5 Hz, 1H), 7.40 (d, *J* = 8.5 Hz, 1H), 7.20-7.00 (m, 5H), 4.22 (d, *J* = 5.5 Hz, 2H), 3.28 (s, 2H). HRMS [ESI], m/z: 311.1499 $[M+H]^+$ for $C_{17}H_{19}N_4O_2$. Anal. Calc. for $C_{17}H_{19}N_4O_2Cl$, %: C, 58.87; H, 5.52; N, 16.15; found, %: C, 58.91; H, 5.60; N, 16.51.

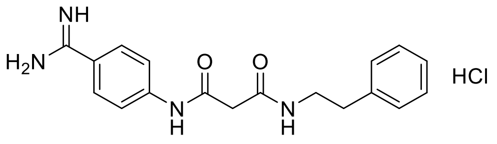

N^1-{4-[amino(imino)methyl]phenyl}-N^3-phenylethyl malonamide hydrochloride (**18c**). White solid, yield 46% (340 mg), M.p. 242–245 °C. IR cm^{-1}: 3231.8, 1676.8, 1624.2, 1522.4, 842.5. ^{1}H NMR (300 MHz, DMSO-d_6), δ_H: 10.77 (s, 1H), 9.25 (s, 2H), 9.00 (s, 2H), 8.30 (t, *J* = 5.5 Hz, 1H), 7.80 (s, 4H), 7.28 – 7.16 (m, 5H), 3.32 – 3.25 (m, 4H). HRMS [ESI], m/z: 325.1662 [M+H]$^+$ for $C_{18}H_{21}N_4O_2$.Anal. Calc. for $C_{18}H_{21}N_4O_2Cl$, %: C, 59.91; H, 5.87; N, 15.52; found, %: C, 59.87; H, 5.80; N, 15.57.

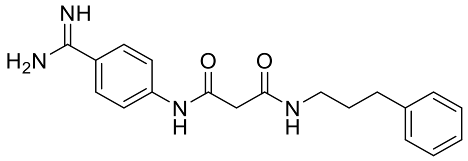

HCl

N^1-{4-[amino(imino)methyl]phenyl}-N^3-[3-(phenyl)]propyl malonamide hydrochloride (**18d**). White solid, yield 47% (350 mg), M.p. 220–223 °C. IR cm^{-1}: 3232, 1675, 1603, 841, 1524. ^{1}H NMR (300 MHz, DMSO-d_6), δ_H: 10.78 (s, 1H), 9.24 (s, 2H), 8.98 (s, 2H), 8.26 (t, *J* = 5.5 Hz, 1H), 7.80 (s, 4H), 7.28 – 7.17 (m, 5H), 3.32 (s, 2H), 3.07 (q, *J* = 5.8 Hz, 2H), 2.58 (t, *J* = 7.5 Hz, 2H), 1.69 (quintet, *J* = 7.5 Hz, 2H). HRMS [ESI], m/z: 339.1820 [M+H]$^+$ for $C_{19}H_{23}N_4O_2$. Anal. Calc. for $C_{19}H_{23}N_4O_2Cl$, %: C, 60.87; H, 6.18; N, 14.94; found, %: C, 61.00; H, 6.44; N, 14.87.

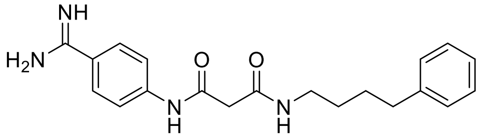

HCl

N^1-{4-[amino(imino)methyl]phenyl}-N^3-[4-(phenyl)butyl] malonamide hydrochloride (**18e**). White crystalline solid, yield 81% (340 mg), M.p. 244 – 246 °C. IR cm^{-1}: 3385.3, 3025.0, 1675.9, 1602.3, 1523.0, 841.2. ^{1}H NMR (300 MHz, DMSO-d_6), δ_H: 10.75 (s, 1H), 9.24 (s, 2H), 8.98 (s, 2H), 8.18 (t, *J* = 5.5 Hz, 1H), 7.80 (s, 4H), 7.26 – 7.11 (m, 5H), 3.29 (s, 2H), 3.08 (q, *J* = 6.0 Hz, 2H), 2.55 (t, J = 7.5 Hz, 2H), 1.56 (quintet, *J* = 7.5 Hz, 2H), 1.41 (quintet, *J* = 7.5 Hz, 2H). HRMS [ESI], m/z: 353.1977 [M+H]$^+$ for $C_{20}H_{25}N_4O_2$. Anal. Calc. for $C_{20}H_{25}N_4O_2Cl$, %: C, 61.77; H, 6.48; N, 14.41; found, %: C, 61.82; H, 6.54; N, 14.67.

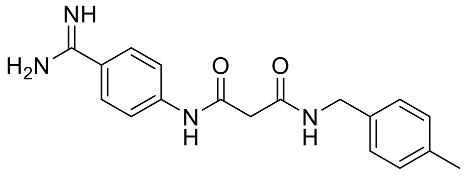

HCl

N^1-{4-[amino(imino)methyl]phenyl}-N^3-(4-methylbenzyl)malonamide hydrochloride (**19a**). Pink solid, yield 61% (290 mg), M.p. 248 – 249.7 °C. IR cm^{-1}: 3363, 3239, 1679, 1625, 1602, 1524, 845, 738. ^{1}H NMR (300 MHz, DMSO-d_6), δ_H: 10.78 (s, 1H), 9.25 (s, 2H), 8.99 (s, 2H), 8.64 (t, 1H, *J* = 5.6 Hz), 7.81 (s, 4H), 7.16 (d, *J* = 8.1 Hz, 2H), 7.10 (d, *J* = 7.8 Hz, 2H), 4.23 (d, *J* = 6.0 Hz, 2H), 3.37 (s, 2H), 2.25 (s, 3H). HRMS [ESI], m/z: 325.1660 [M+H]$^+$ for $C_{18}H_{21}N_4O_2$.Anal. Calc. for $C_{18}H_{21}N_4O_2Cl$, %: C, 59.91; H, 5.87; N, 15.52; found, %: C, 59.97; H, 5.91; N, 15.66.

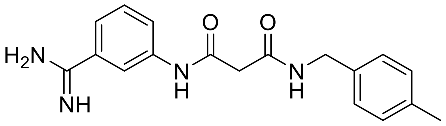

N¹-(3-carbamimidoil-phenyl)-N³-(4-mehyl-benzyl) malonamide hydrochloride (**19b**) White solid, yield 20% (0.140 g) m.p. = 162-164 °C. IR cm^{-1} : 3292.2, 3066.2, 1678.4, 1625.3, 802.5; ^{1}H-NMR (300 MHz, DMSO-d6) δ : 10.64 (s, 1H), 9.36 (s, 2H), 9.09 (s, 2H), 8.63 (t, *J* = 5.5 Hz,1H), 8.09 (s, 1H), 7.83 (d, *J* = 9 Hz 1H), 7.53 (t, *J* = 9 Hz, 1H), 7.41 (d, *J* = 9 Hz, 1H), 7.17-7.09 (q, *J* = 9 Hz, 4H), 4.24 (d, *J* = 3 Hz, 2H), 3.28 (s, 2H), 2.06 (s, 3H). HRMS [ESI], m/z: 325.1661 [M+H]$^{+}$ for $C_{18}H_{21}N_4O_2$.Anal. Calc. for $C_{18}H_{21}N_4O_2Cl$, %: C, 59.91; H, 5.87; N, 15.52; found, %: C, 60.07; H, 5.95; N, 15.56.

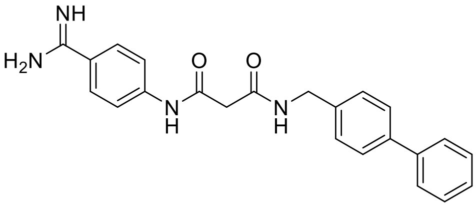

N¹-{3-[amino(imino)methyl]phenyl}-N³-biphenylmethylenmalonamide hydrochloride (**19c**). White solid, yield 55% (165 mg). M.p. 164-165 °C. IR cm^{-1} : 3292, 1678, 1630, 800; ^{1}H NMR (300 MHz, DMSO-d$_6$), δ_H: 10.80 (s, 1H), 9.40 (s, 2H), 9.11 (s, 2H), 8.67 (t, 1H, *J* = 5.5 Hz), 7.84 (s, 4H), 7.40 (d, *J* = 8.5 Hz, 2H), 7.40-7.25 (m, 5H), 7.16 (d, *J* = 8.5 Hz, 2H), 4.23 (d, *J* = 5.5 Hz, 2H), 3.37 (s, 2H). HRMS [ESI], m/z: 387.1822 [M+H]$^{+}$ for $C_{23}H_{23}N_4O_2$.Anal. Calc. for $C_{23}H_{23}N_4O_2Cl$, %: C, 65.32; H, 5.48; N, 13.25; found, %: C, 65.39; H, 5.55; N, 13.44.

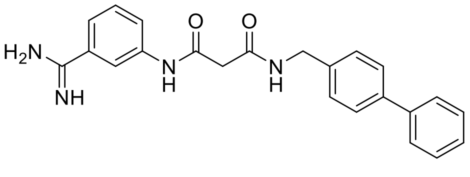

N¹-{3-[amino(imino)methyl]phenyl}-N³-biphenylmethylenmalonamide hydrochloride (**19d**). Brown solid, yield 43% (110 mg). M.p. 170-180 °C. IR cm^{-1} : 3290, 1680, 1627, 805; . ^{1}H NMR (300 MHz, DMSO-d$_6$), δ_H: 10.78 (s, 1H), 9.36 (s, 2H), 9.06 (s, 2H), 8.74 (m, 1H), 8.05 (s, 1H), 7.84 (d, J = 7.9 Hz, 1H), 7.3 – 7.7 (m, 12H), 4.33 (d, *J* = 5.8 Hz, 2H), 3.38 (s, 2H). HRMS [ESI], m/z: 387.1821 [M+H]$^{+}$ for $C_{23}H_{23}N_4O_2$.Anal. Calc. for $C_{23}H_{23}N_4O_2Cl$, %: C, 65.32; H, 5.48; N, 13.25; found, %: C, 65.45; H, 5.65; N, 13.52.

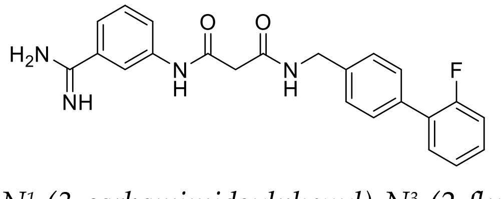

N¹-(3-carbamimidoylphenyl)-N³-(2-fluorobenzyl)malonamide (**19e**). Pale brown solid, yield 33% (85 mg) M.p. 88–91 °C. IR cm^{-1}: 3112, 1677, 1645, , 1548, 1484, 1407, 1251, 753, 694. ^{1}H NMR

(300 MHz, DMSO-d6) δ 10.85 (d, J = 5.3 Hz, 1H), 9.42 (s, 2H), 9.27 (s, 2H), 8.84 (t, J = 5.0 Hz, 1H), 8.09 (s, 1H), 7.93 – 7.14 (m, 7H), 4.34 (d, J = 5.8 Hz, 3H), 3.43 (s, 4H). HRMS [ESI], m/z: 405.1721 $[M+H]^+$ for $C_{23}H_{22}N_4O_2F$. Anal. Calc. for $C_{23}H_{22}N_4O_2FCl$, %: C, 68.13; H, 5.47; N, 13.82; found, %: C, 68.35; H, 5.65; N, 13.62.

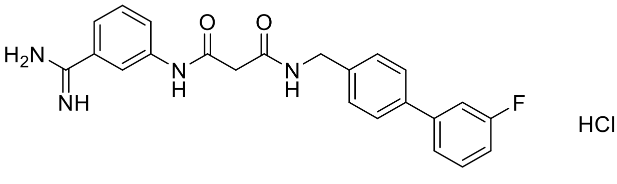

N¹-(3-carbamimidoylphenyl)-N³-(3-fluorobenzyl)malonamide hydrochloride (**19f**). Brown powder, yield 32% (110 mg). M.p. 165–170 °C. IR cm^{-1}: 3139, 3053, 1678, 1651, 1548, 1403, 1259, 1188, 880, 776, 715, 679. ^1H NMR (300 MHz, DMSO-d6) δ 10.82 (s, 1H), 9.45 (s, 2H), 9.32 (s, 2H),, 8.82 (t, J = 5.8 Hz, 1H), 8.11 (d, J = 7.4 Hz, 1H), 7.98 - 7.34 (m, 6H), 7.17 (tt, J = 4.7, 4.1 Hz, 1H), 4.34 (d, J = 5.9 Hz, 2H), 3.42 (s, 1H). HRMS [ESI], m/z: 405.1723 $[M+H]^+$ for $C_{23}H_{22}N_4O_2F$. Anal. Calc. for $C_{23}H_{22}N_4O_2FCl$, %: C, 68.13; H, 5.47; N, 13.82; found, %: C, 68.24; H, 5.61; N, 13.91.

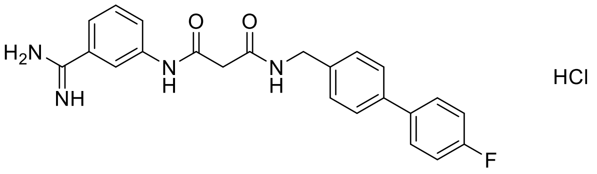

N¹-(3-carbamimidoylphenyl)-*N³*-((4'-fluoro-[1,1'-biphenyl]-4-yl)methyl)malonamide hydrochloride (**19g**). Brown powder, yield 26% (74 mg). M.p. 250 °C dec. IR cm^{-1}:3399, 1895, 1672, 1638, 1602, 1555, 1499, 1433, 1251, 805, 721, 676. ^1H NMR (300 MHz, DMSO-d6), δ_H: 10.79 (s, 1H), 9.40 (s, 2H), 9.21 (s, 2H), 8.80 (t, *J* = 5.5 Hz, 1H), 7.7 – 7.0 (m, 11H), 4.33 (d, *J* = 5.8 Hz, 2H), 3.35 (s, 2H). HRMS [ESI], m/z: 405.1719 $[M+H]^+$ for $C_{23}H_{22}N_4O_2F$. Anal. Calc. for $C_{23}H_{22}N_4O_2FCl$, %: C, 68.13; H, 5.47; N, 13.82; found, %: C, 68.21; H, 5.55; N, 13.99.

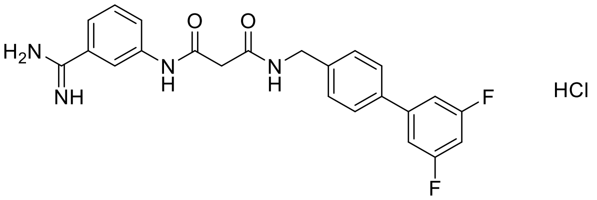

N¹-{3-[amino(imino)methyl]phenyl}-N³-(3′,5′-difluoro) biphenylmethylenmalonamide hydrochloride (**19i**). Brown solid, yield 68% (200 mg). M.p. 160-165 °C.. IR cm^{-1}: 3276, 1677, 1646, 965, 805, 701; ^1H NMR (300 MHz, DMSO-d_6), δ_H: 10.71 (s, 1H), 9.37 (s, 2H),9.12 (s, 2H),8.78 (t, *J* = 5.8 Hz, 1H), 8.10 (s, 1H), 7.85 (m, 1H), 7.69 (d, *J* = 8.3 Hz, 2H), 7.39 (d, *J* = 8.3 Hz, 2H), 7.2 (m, 1H), 4.34 (d, *J* = 5.8 Hz, 2H), 3.40 (s, 2H). HRMS [ESI], m/z: 423.1629 $[M+H]^+$

for $C_{23}H_{21}N_4O_2F_2$. Anal. Calc. for $C_{23}H_{22}N_4O_2F_2Cl$, %: C, 60.20; H, 4.61; N, 12.21; found, %: C, 60.23; H, 4.72; N, 12.19.

19l.

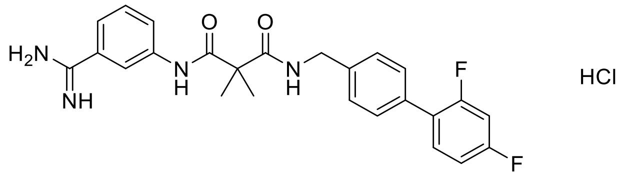

N¹-(3-carbamimidoylphenyl)-N³-((2′,4′-difluoro-[1,1′-biphenyl]-4-yl)methyl)-2,2-dimethylmalonamide hydrochloride (**19l**). Pale brown solid, yield 43% (110 mg). M.p. 132-136 °C. IR cm^{-1}: 3273, 3074, 1673, 1595, 1494, 1425, 1403, 1297, 1262, 1140, 1100, 964, 801, 720, 678. ^{1}H NMR (300 MHz, DMSO-d6) δ 9.81 (s, 1H), 9.13 (br s, 4H), 8.41 (t, *J* = 5.9 Hz, 1H), 8.15 (t, *J* = 1.8 Hz, 1H), 7.95 (d, *J* = 8.1 Hz, 1H), 7.58 – 7.47 (m, 2H), 7.46 – 7.39 (m, 3H), 7.38 – 7.29 (m, 3H), 7.17 (td, *J* = 8.4, 2.7 Hz, 1H), 4.34 (d, *J* = 5.8 Hz, 2H), 3.31 (s, 1H), 1.48 (s, 6H). HRMS [ESI], m/z: 451.1942 $[M+H]^+$ for $C_{25}H_{25}N_4O_2F_2$. Anal. Calc. for $C_{25}H_{25}N_4O_2F_2Cl$, %: C, 61.66; H, 5.17; N, 11.51; found, %: C, 61.97; H, 5.36; N, 11.50.

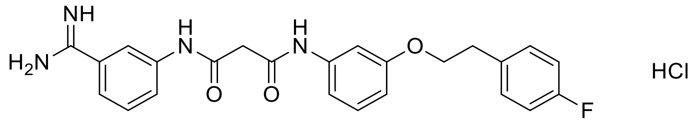

*N¹-{3-[amino(imino)methyl]phenyl}-N³-{3-[2-(4-fluoro-phenyl)-ethossi]-phenyl}-malonammide (**20a**). hydrochloride* (**20a**). Pale brown solid, yield 61% (290 mg). M.p. 108-110 °C. IR cm^{-1}: 3258, 2931, 1676, 1598, 1551, 1510, 1481, 1220, 1157, 1038, 721, 687. ^{1}H NMR (300 MHz, DMSO-d6), δ_H: δ 10.89 (s, 1H), 10.49 (s, 1H), 9.39 (s, 2H), 9.18 (s, 2H), 8.12 (s, 1H), 7.86 (d, J=3HZ, 1H), 7.59-7.55 (t, 1H), 7.52 (d, J=6Hz, 1H), 7.48-7.28 (m, 3H), 7.21-7.06 (m, 4H), 6.61(d, J=6Hz, 1H), 4.11-4.07 (t, J=6Hz, 2H), 3.54 (s, 2H), 3.01-2.97 (t, J=6Hz, 2H). HRMS [ESI], m/z: 435.1829 $[M+H]^+$ for $C_{24}H_{24}N_4O_3F$. Anal. Calc. for $C_{25}H_{25}N_4O_2FCl$, %: C, 61.21; H, 5.14; N, 11.90; found, %: C, 61.37; H, 5.26; N, 11.79.

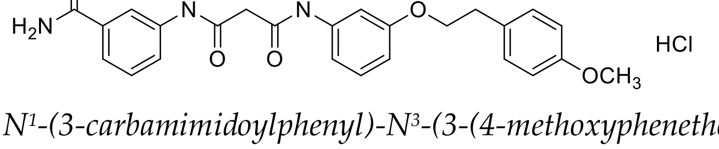

N¹-(3-carbamimidoylphenyl)-N³-(3-(4-methoxyphenethoxy)phenyl)malonamide hydrochloride (**20b**). Brown solid, yield 44% (120 mg). M.p. 93-95 °C. IR cm^{-1}:3017, 1682, 1645, 1555, 1402, 1248, 1034, 753, 682. ^{1}H NMR (300 MHz, DMSO-d6) δ 10.85 (s, 1H), 10.45 (s, 1H), 9.38 (s, 2H), 9.15 (s, 2H), 8.09 (dt, *J* = 16.5, 1.6 Hz, 1H), 7.86 (t, *J* = 7.4 Hz, 1H), 7.59 – 7.25 (m, 7H), 7.21 (d,

J = 8.6 Hz, 2H), 6.85 (d, J = 8.7 Hz, 3H), 6.61 (d, J = 7.7 Hz, 2H), 4.06 (t, J = 6.9 Hz, 2H), 3.70 (s, 3H), 3.54 (s, 2H), 2.93 (t, J = 6.9 Hz, 2H). HRMS [ESI], m/z: 447.2028 $[M+H]^+$ for $C_{25}H_{27}N_4O_4$. Anal. Calc. for $C_{25}H_{27}N_4O_4Cl$, %: C, 62.17; H, 5.63; N, 11.60; found, %: C, 62.27; H, 5.86; N, 11.77.

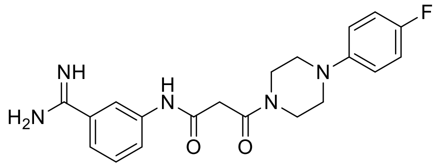

HCl

N-{4-[amino(imino)methyl]phenyl}-3-[4-(4-fluorophenyl)piperazin-1-yl] propanamide hydrochloride (**21a**). Brown solid, yield 20% (160 mg), M.p. 148-151 °C. IR cm^{-1}: 3444, 1682, 1627,1560, 1510, 1450, 1232, 826, 725. ^1H NMR (300 MHz, DMSO-d_6), δ_H: 10.52 (s, 1H), 7.98 (s, 1H), 7.82 (d, J = 7.98 Hz, 1H), 7.51 (t, J = 7.98 Hz, 1H), 7.39 (d, J = 7.98 Hz, 1H), 7.08-6.95 (m, 4H), 3.65 – 3.57 (m, 4H), 3.15-3.00 (m, 6H). HRMS [ESI], m/z: 384.1832 $[M+H]^+$ for $C_{20}H_{23}N_5O_2F$. Anal. Calc. for $C_{20}H_{23}N_5O_2FCl$, %: C, 57.21; H, 5.52; N, 16.68; found, %: C, 57.27; H, 5.82; N, 16.79.

22a.

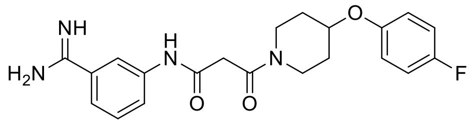

HCl

N-{3-[amino(imino)methyl]phenyl}-3-(4-(4-fluorophenoxy)piperidin-1-yl)-3-oxopropanamide hydrochloride (**22a**). Dark brown oil, yield 45% (112 mg). IR cm^{-1}: 3423, 1674, 1619, 1560, 1504, 1460, 1205, 1027, 873, 828, 802, 763. ^1H NMR (300 MHz, DMSO-d6), δ_H: 10.75 (s, 1 H), 9.39 (s, 2 H), 9.21 (s, 2 H), 8.09 (s, 1 H), 7.84 (d, J=7.7 Hz, 1 H), 7.51 (t, J=8.2 Hz, 2 H), 7.15 (m, 2 H), 6.99 (m, 2 H), 4.56 (s, 2 H), 3.85 (m, 1 H), 3.68 (m, 1 H), 3.54 (s, 2 H), 3.30 (m, 2 H), 1.90 (m, 2 H), 1.65 (m, 1 H), 1.48 (m, 1 H). HRMS [ESI], m/z: 399.1831 $[M+H]^+$ for $C_{21}H_{24}N_4O_3F$.

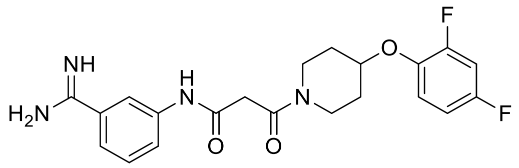

HCl

N-(3-carbamimidoylphenyl)-3-(4-(2,4-difluorophenoxy)piperidin-1-yl)-3-oxopropanamide hydrochloride (**22b**). Dark brown oil, yield 90% (210 mg). IR cm^{-1}: 3420, 1675, 1620, 1200, 1024, 703. ^1H NMR (300 MHz, DMSO-d6), δ_H: 10.78 (s, 1H), 9.37 (s, 2H), 9.14 (s, 2H), 8.08 (s, 1H), 7.84 – 7.82 (m, 1H), 7.53 (t, J = 7.8 Hz, 1H), 7.41 (d, J = 7.7 Hz, 1H), 7.38 – 7.23 (m, 2H), 7.05 –

6.95 (m, 1H), 5.32 (br s, 2H), 4.56 (quintet, *J* = 3.6 Hz, 1H), 3.98 – 3.65 (m, 2H), 3.61 (s, 2H), 3.33 – 3.12 (m , 2H), 2.00 – 1.83 (m, 1H), 1.69 – 1.47 (m, 1H). HRMS [ESI], m/z: 417.1731 $[M+H]^+$ for $C_{21}H_{23}N_4O_3F_2$.

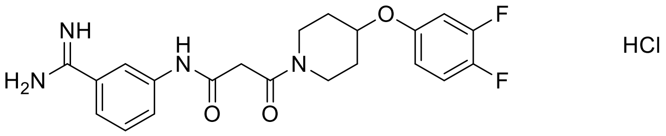

N-(3-carbamimidoylphenyl)-3-(4-(3,4-difluorophenoxy)piperidin-1-yl)-3-oxopropanamide hydrochloride (**22c**). Dark brown oil, yield 37% (251 mg). IR cm^{-1}: 3422, 1673, 1625, 1200, 1025, 801. ^1H NMR (300 MHz, DMSO-d6), δ_H: 10.68 (s, 1H), 10.31 (s, 1H), 9.37 (s, 2H), 9.13 (s, 2H), 8.08 (s, 1H), 7.83 (d, *J* = 9.1 Hz, 1H), 7.53 (t, *J* = 8.0 Hz, 1H), 7.41 (d, *J* = 8.0 Hz, 1H), 7.38 -7.25 (m, 1H), 7.30 – 7.08 (m, 1H), 6.84 – 6.78 (m, 1H), 4.6 (quintet, *J* = 4.5 Hz, 1H), 3.90 – 3.65 (m, 2H), 3.54 (s, 2H), 3.30 – 3.10 (m, 2H), 2.0 – 1.8 (m, 2H), 1.70 – 1.40 (m, 2H). HRMS [ESI], m/z: 417.1735 $[M+H]^+$ for $C_{21}H_{23}N_4O_3F_2$.

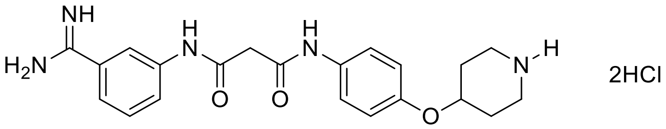

N^1*-{3-[amino(imino)methyl]phenyl}-N*^{3}*-[4-(piperidin-4-iloxy)-phenyl]-malonamide dihydrochloride* (**23a**). Dark brown oil, yield 15% (60 mg). IR cm^{-1}: 3288, 2974, 1689, 1553, 1508, 1479, 1414, 1234, 1169, 1031, 831, 802, 770, 723, 654. ^1H NMR (300 MHz, DMSO-d_6), δ_H: 10.85 (s, 1H), 10.34 (s, 1H), 9.38 (s, 2H), 9.15 (s, 2H), 7.41-8.12 (m, 4H), 7.53 (d, *J* = 9.0 Hz, 2H), 6.94 (d, *J* = 9.0 Hz, 2H), 3.52 (s, 2H), 4.55 (quintet, *J* = 3.3 Hz, 1H), 3.17 (br s, 2H), 3.03 (br s, 2H), 2.08 (m, 2H), 1.80 (m, 2H). HRMS [ESI], m/z: 396.2032 $[M+H]^+$ for $C_{21}H_{26}N_5O_3$.

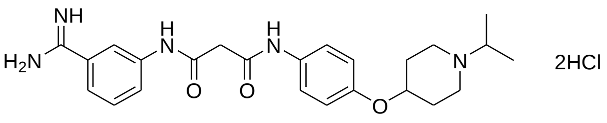

N^1*-{3-[amino(imino)methyl]phenyl}-N*^{3}*-[4-(1-isopropylpiperidin-4-iloxy)phenyl]malonamide hydrochloride* (**23b**). Dark brown oil, yield 22% (120 mg). ^1H NMR (300 MHz, DMSO-d_6), δ_H: 10,83 (s, 1H), 10,35 (d, *J* = 6,87Hz, 1H), 9.37 (s, 2H), 9.12 (s, 2H), 7.6-7.3 (m, 8H), 3.51 (s, 2H), 3.44 (m, 6H), 1.28 (m, 5H), 1.02(m, 4H). HRMS [ESI], m/z: 438.2506 $[M + H]^+$ for $C_{24}H_{32}N_5O_3^+$.

Table S1. FXa and thrombin inhibition of succinimide analogues.

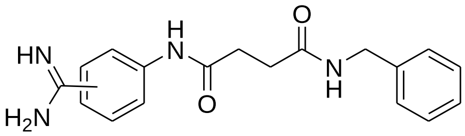

Cmpd	amidine	K_i (μM)[a]	
		fXa	thr
**S1**	p	>50 (10% at 50 μM)	>50 (5% at 50 μM)
**S2**	m	>50 (8% at 50 μM)	>50 (7% at 50 μM)
**Apixaban**[b]		0.00012	
**Dabigatran**[b]			0.0042

[a]Ki values determined by applying the Cheng–Prousoff equation to IC_{50} values, by regression (GraphPad Prism software ver. 5.01). Data are means of three independent measurements, each one performed in triplicate (SEM < 5%). [b]Apixaban and dabigatran have been tested as positive controls against blood coagulation factors fXa and thrombin, respectively.

HNMR spectra of selected compounds

4b

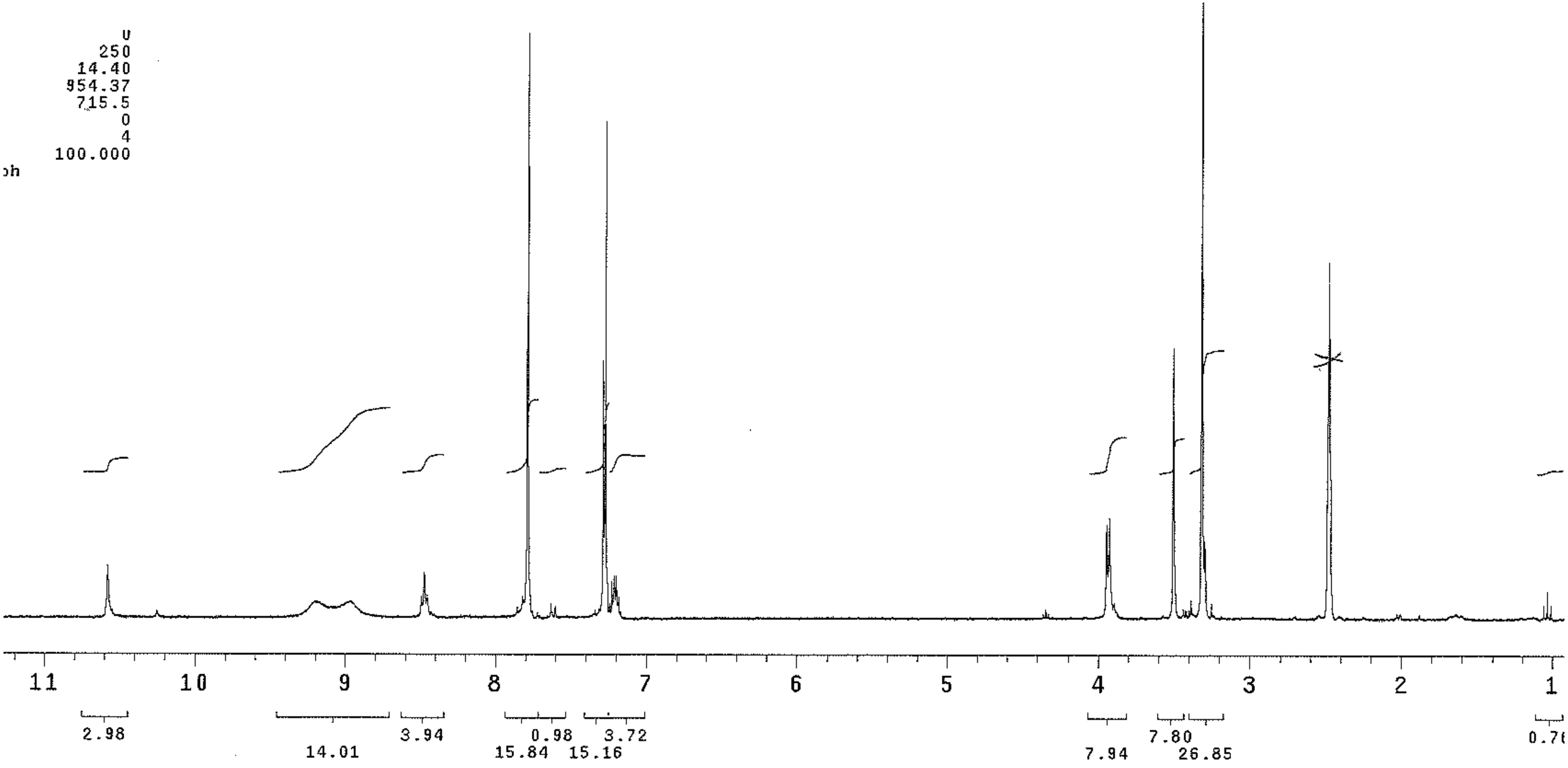

4c

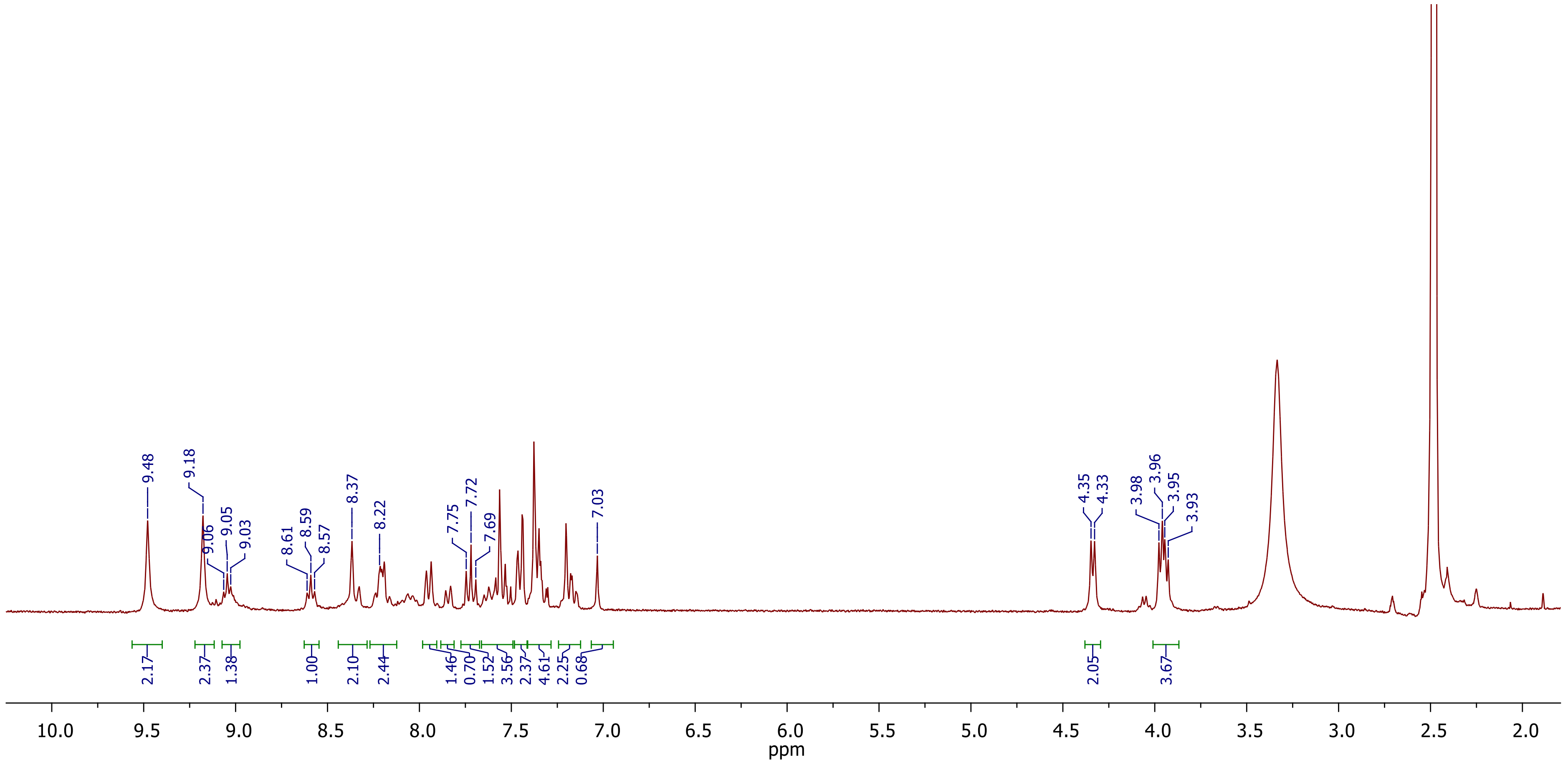
9.48
9.18
9.06
9.05
9.03
8.61
8.59
8.57
8.37
8.22
7.75
7.72
7.69
7.03
4.35
4.33
3.98
3.96
3.95
3.93
2.17
2.37
1.38
1.00
2.10
2.44
1.46
0.70
1.52
3.56
2.37
4.61
2.25
0.68
2.05
3.67
10.0
9.5
9.0
8.5
8.0
7.5
7.0
6.5
6.0
5.5
5.0
4.5
4.0
3.5
3.0
2.5
2.0
ppm

18c

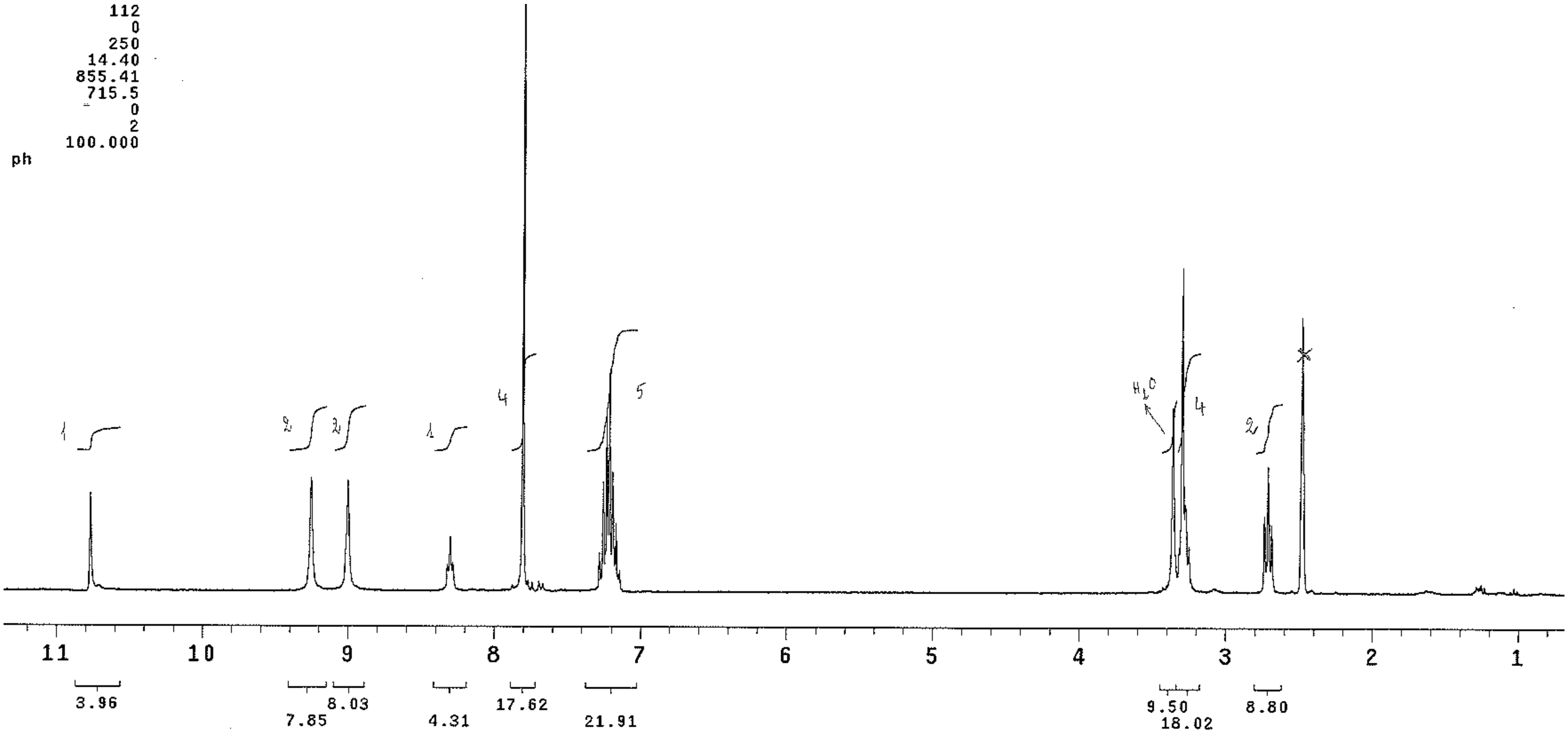

112
0
250
14.40
855.41
715.5
0
2
100.000
ph
11
10
9
8
7
6
5
4
3
2
1
3.96
7.85
8.03
4.31
17.62
21.91
9.50
18.02
8.80

18d

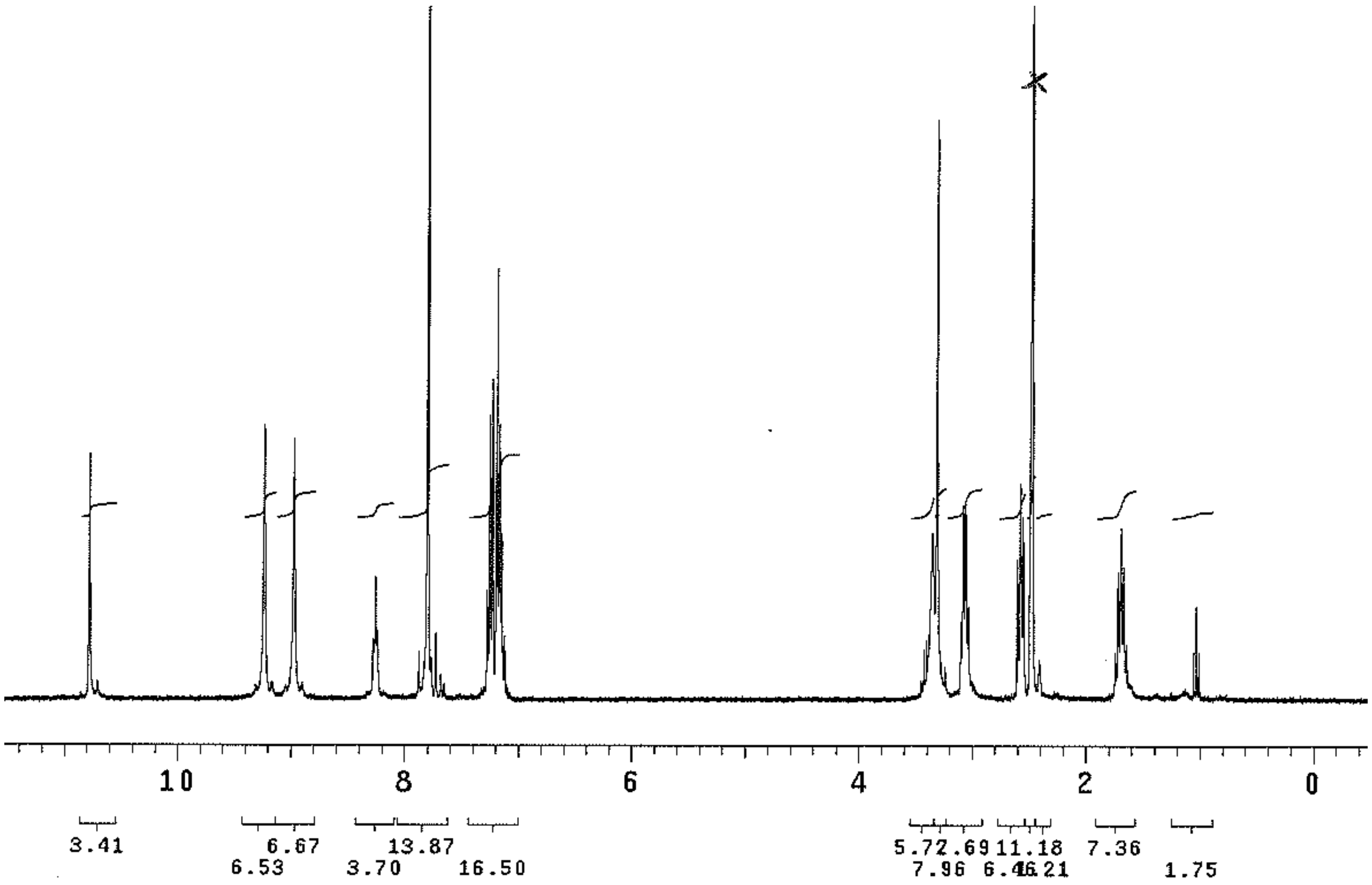

10
8
6
4
2
0
3.41
6.67
6.53
13.87
3.70
16.50
5.72
2.69
11.18
7.96
6.46
16.21
7.36
1.75

18e

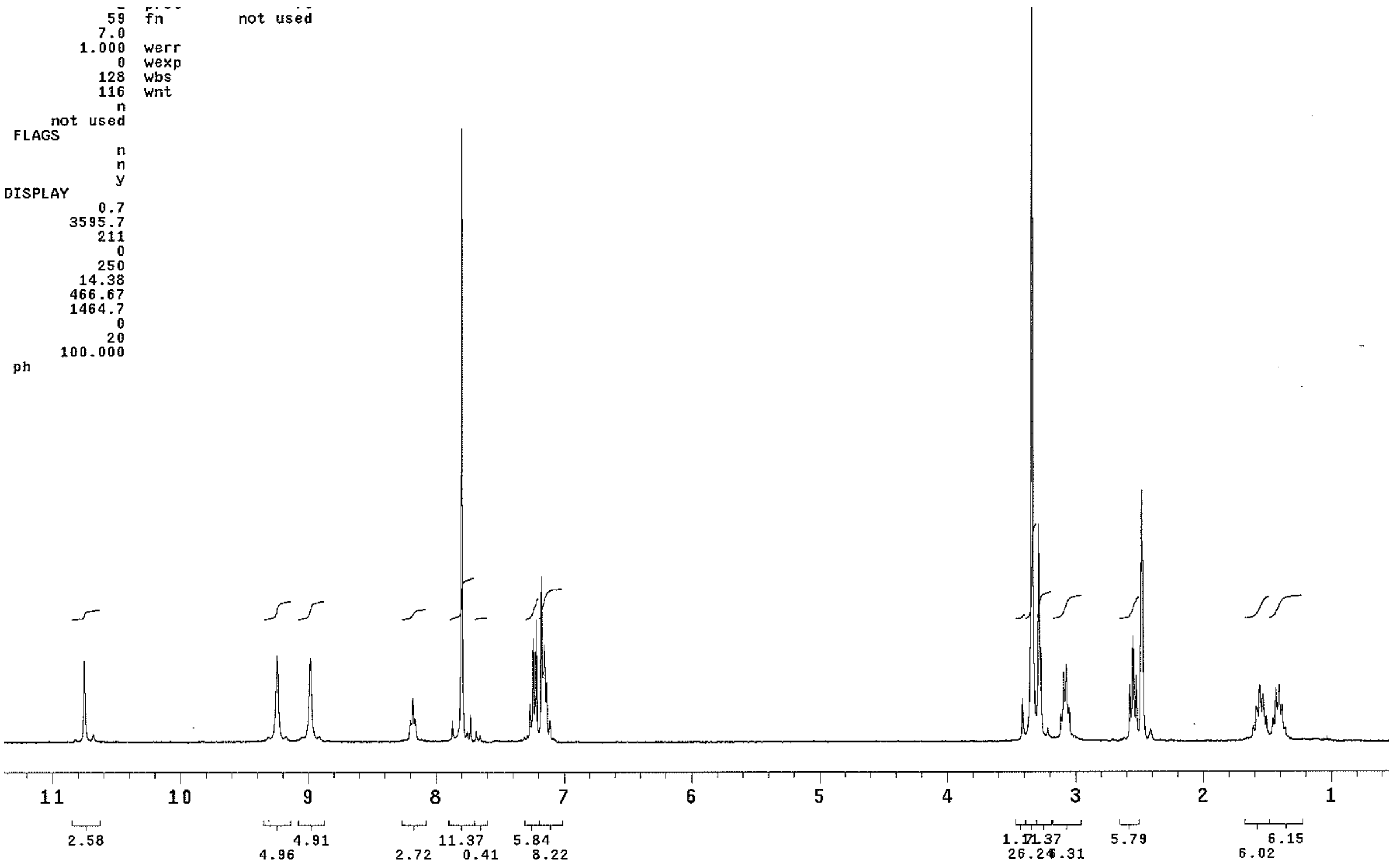
59 fn not used
7.0
1.000 werr
0 wexp
128 wbs
116 wnt
n
not used
FLAGS
n
n
y
DISPLAY
0.7
3595.7
211
0
250
14.38
466.67
1464.7
0
20
100.000
ph
11 10 9 8 7 6 5 4 3 2 1
2.58
4.96 4.91
2.72 11.37 0.41
5.84 8.22
1.17 1.37
26.24 6.31
5.79
6.02 6.15

18d

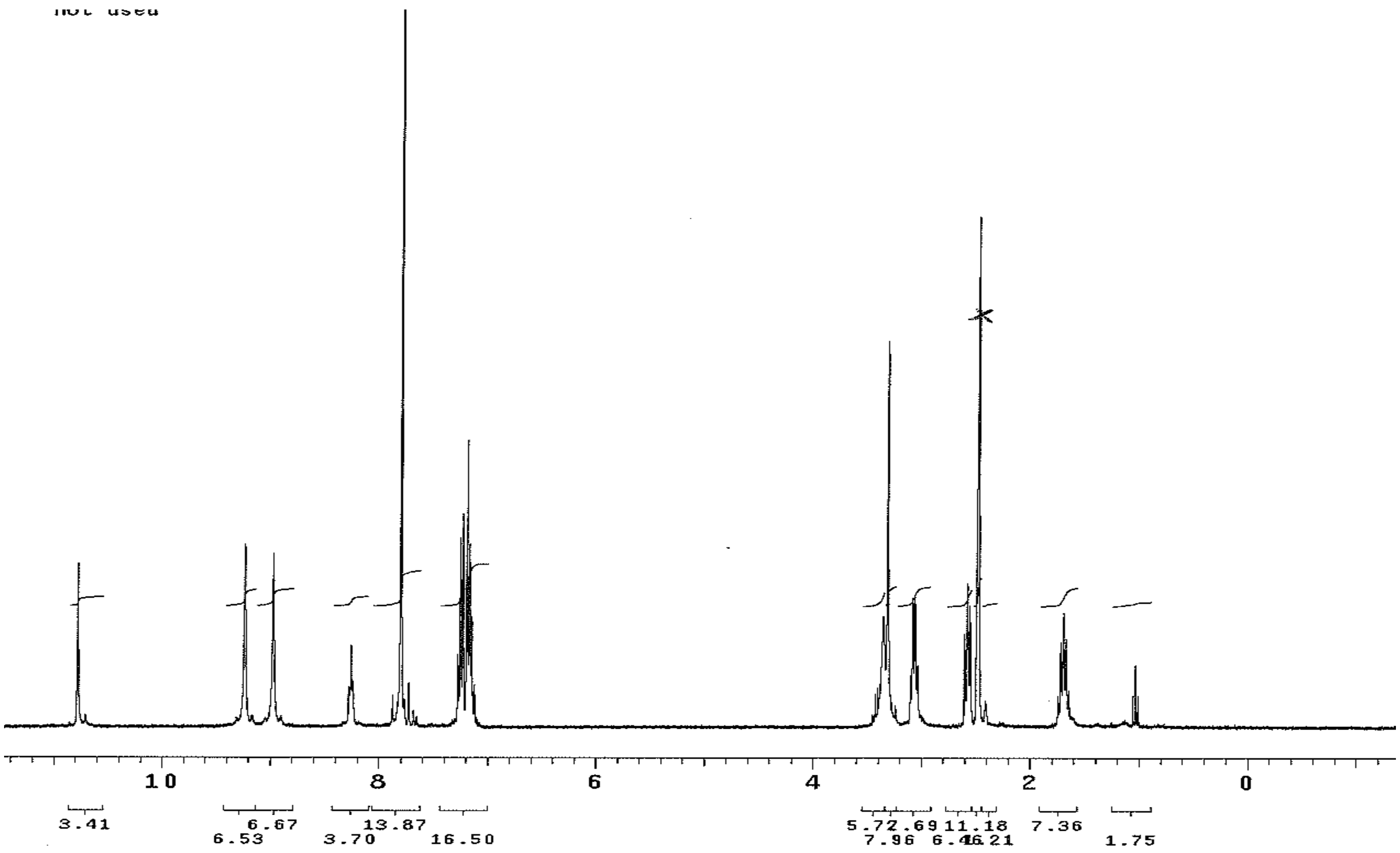

19a

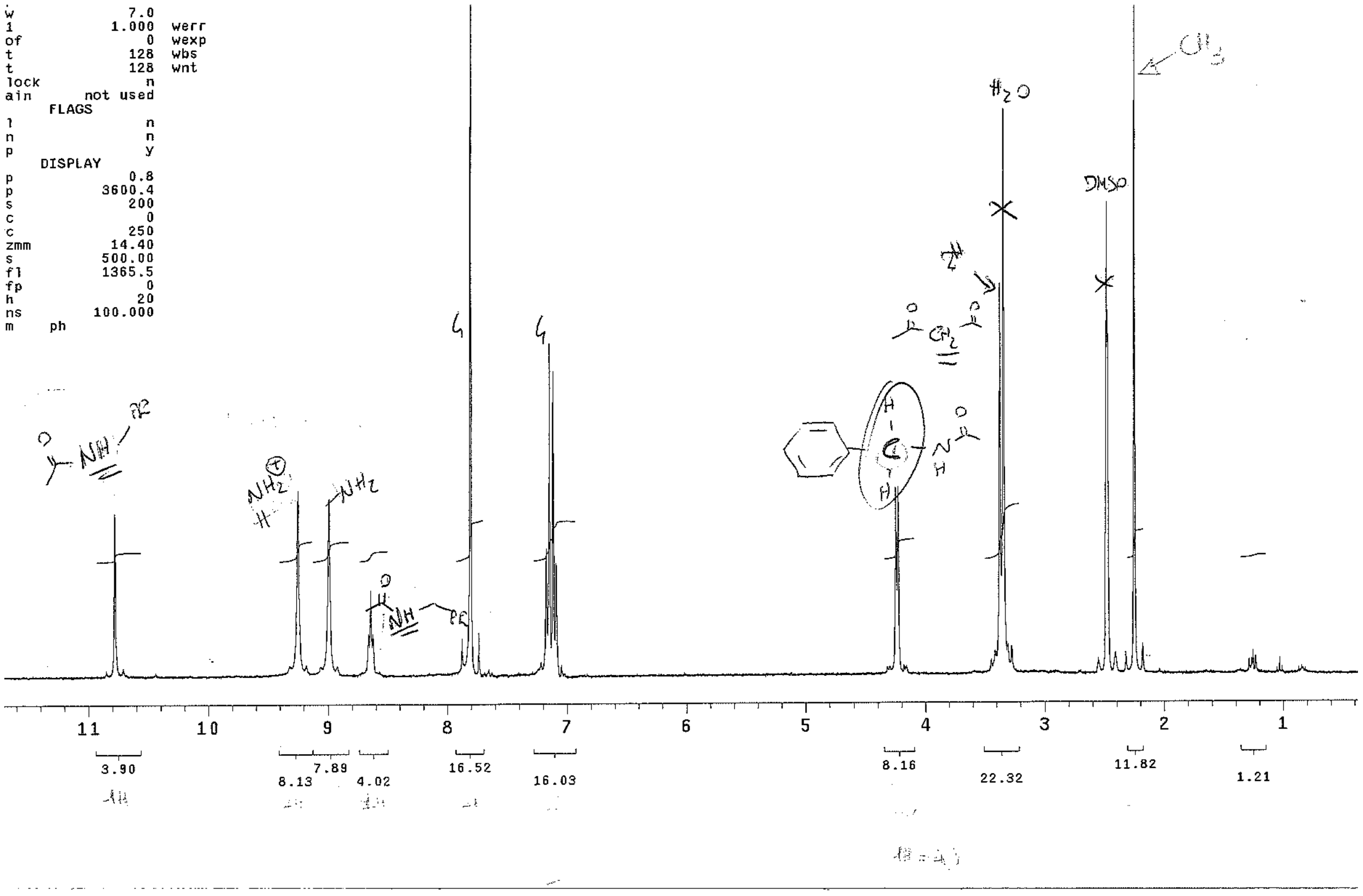
w 7.0
1 1.000 werr
of 0 wexp
t 128 wbs
t 128 wnt
lock n
ain not used
FLAGS
l n
n n
p y
DISPLAY
p 0.8
p 3600.4
s 200
c 0
c 250
zmm 14.40
s 500.00
fl 1365.5
fp 0
h 20
ns 100.000
m ph
11 10 9 8 7 6 5 4 3 2 1
3.90
8.13
7.89
4.02
16.52
16.03
8.16
22.32
11.82
1.21

19d

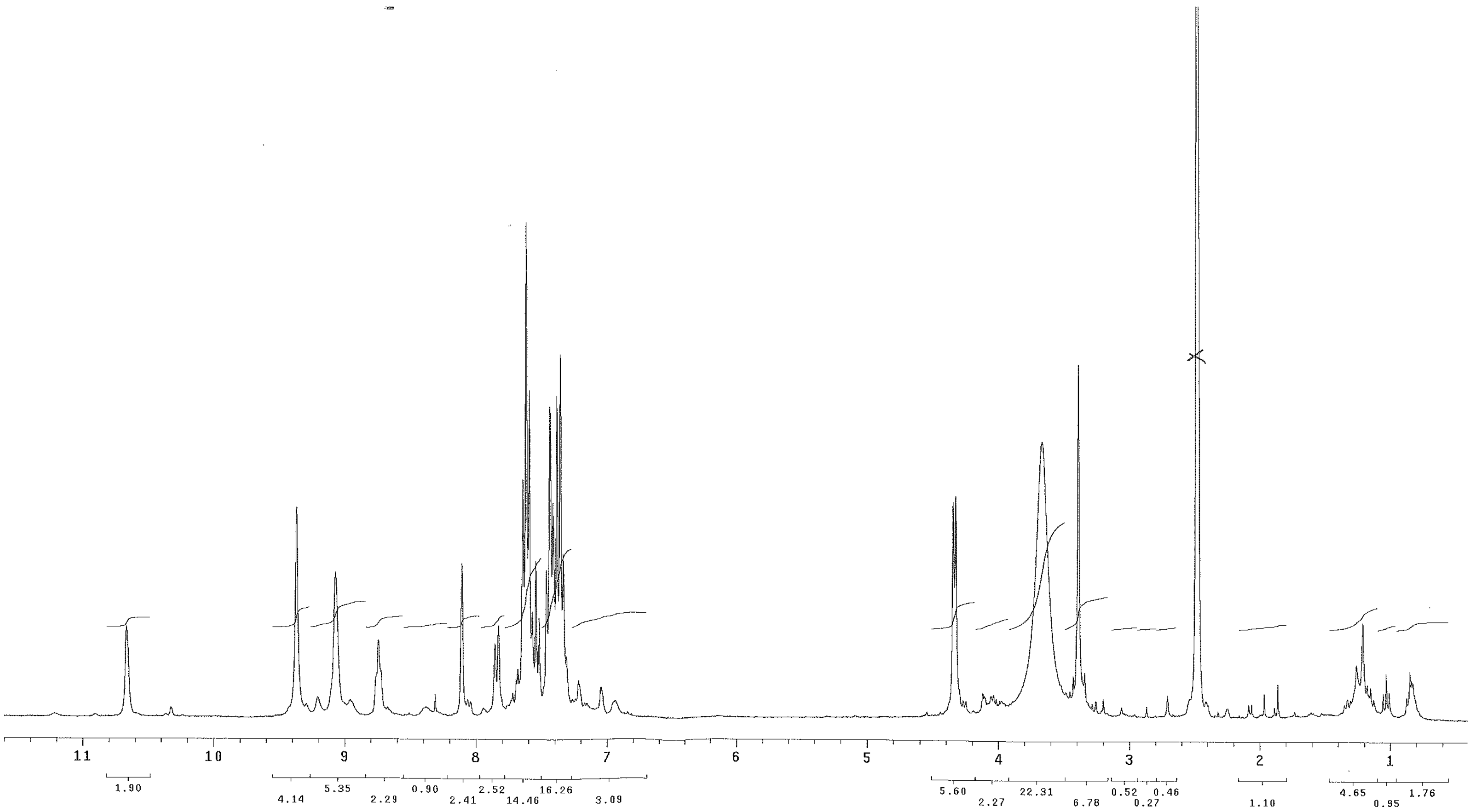
11
10
9
8
7
6
5
4
3
2
1
1.90
4.14
5.35
2.29
0.90
2.41
2.52
14.46
16.26
3.09
5.60
2.27
22.31
6.78
0.52
0.27
0.46
1.10
4.65
0.95
1.76

19e.

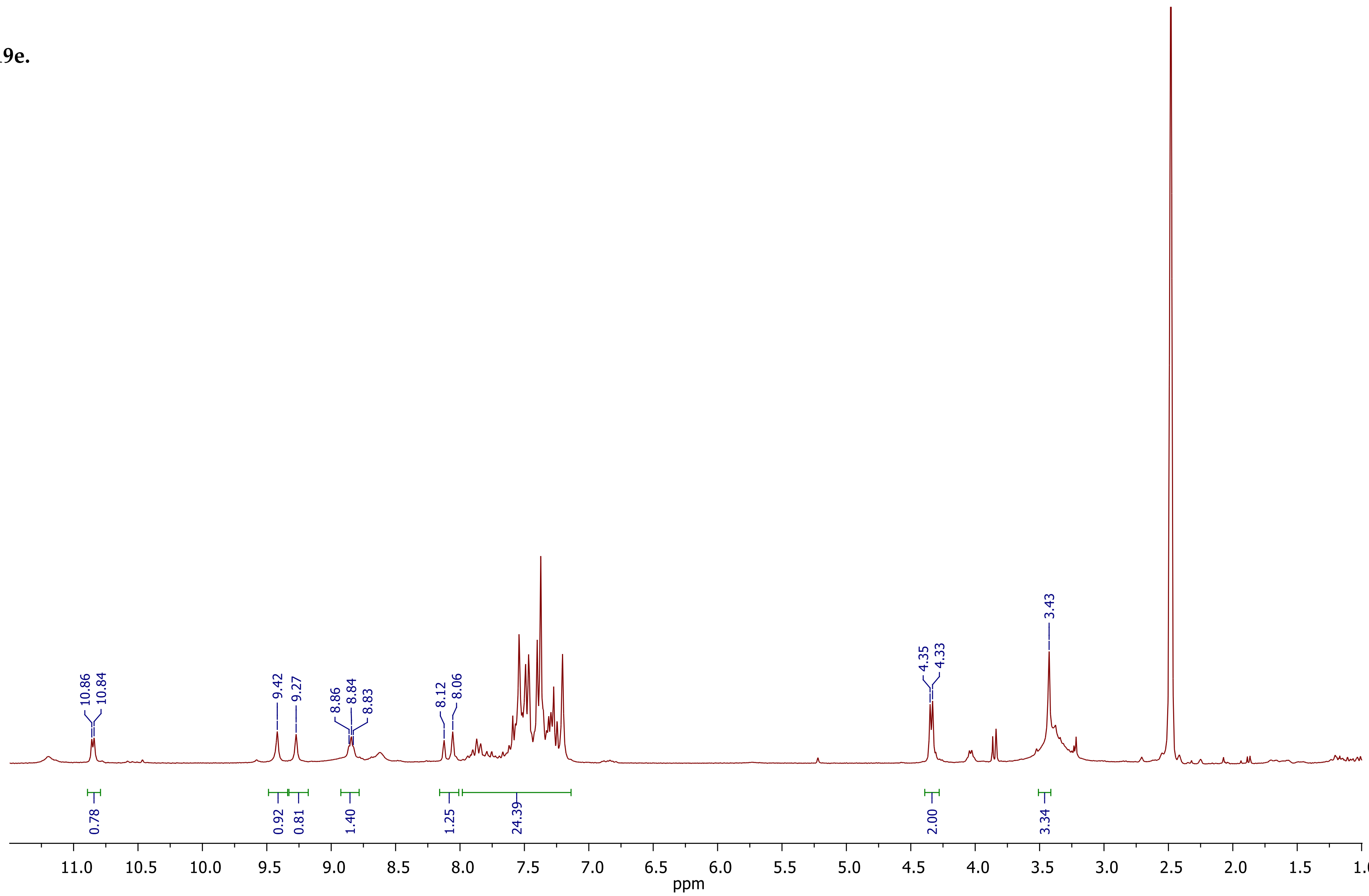
10.86
10.84
9.42
9.27
8.86
8.84
8.83
8.12
8.06
4.35
4.33
3.43
0.78
0.92
0.81
1.40
1.25
24.39
2.00
3.34
11.0
10.5
10.0
9.5
9.0
8.5
8.0
7.5
7.0
6.5
6.0
5.5
5.0
4.5
4.0
3.5
3.0
2.5
2.0
1.5
1.0
ppm

19f

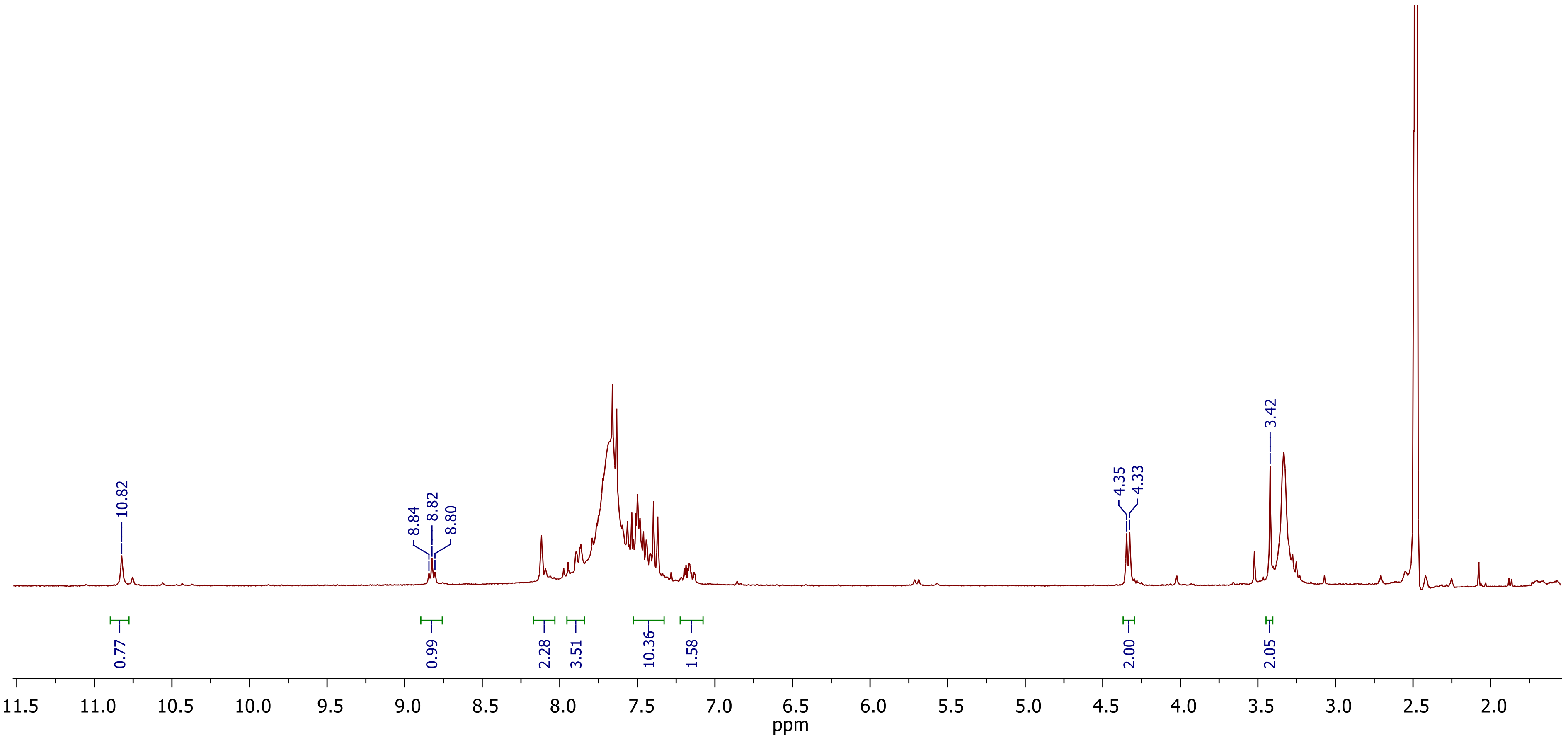
10.82
8.84
8.82
8.80
4.35
4.33
3.42
0.77
0.99
2.28
3.51
10.36
1.58
2.00
2.05
11.5
11.0
10.5
10.0
9.5
9.0
8.5
8.0
7.5
7.0
6.5
6.0
5.5
5.0
4.5
4.0
3.5
3.0
2.5
2.0
ppm

19g

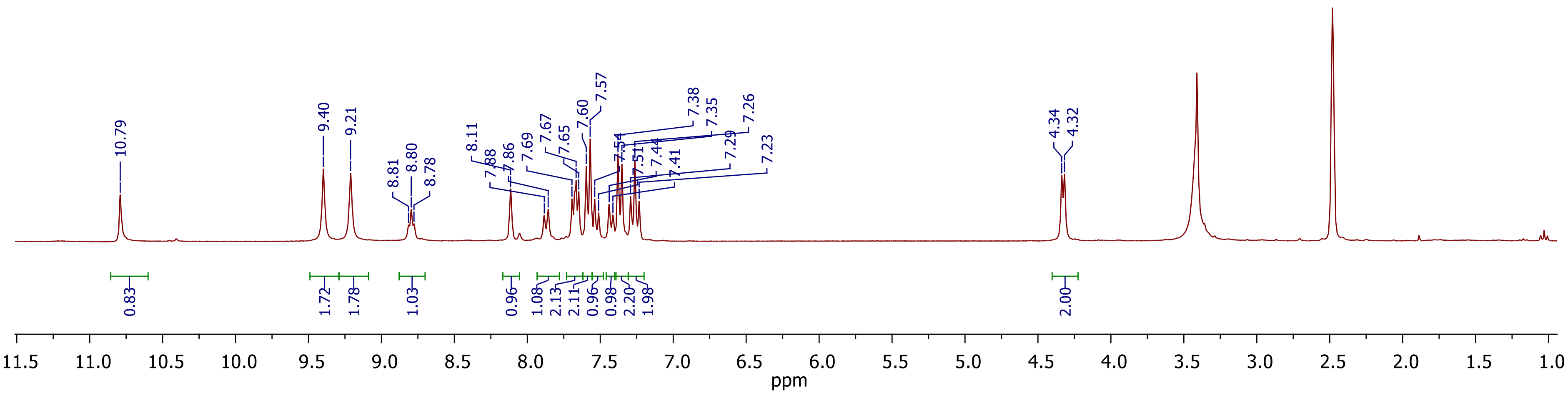
10.79
9.40
9.21
8.81
8.80
8.78
8.11
7.88
7.86
7.69
7.67
7.65
7.60
7.57
7.54
7.51
7.44
7.41
7.38
7.35
7.29
7.26
7.23
4.34
4.32
0.83
1.72
1.78
1.03
0.96
1.08
2.13
2.11
0.96
0.98
2.20
1.98
2.00
11.5
11.0
10.5
10.0
9.5
9.0
8.5
8.0
7.5
7.0
6.5
6.0
5.5
5.0
4.5
4.0
3.5
3.0
2.5
2.0
1.5
1.0
ppm

19i

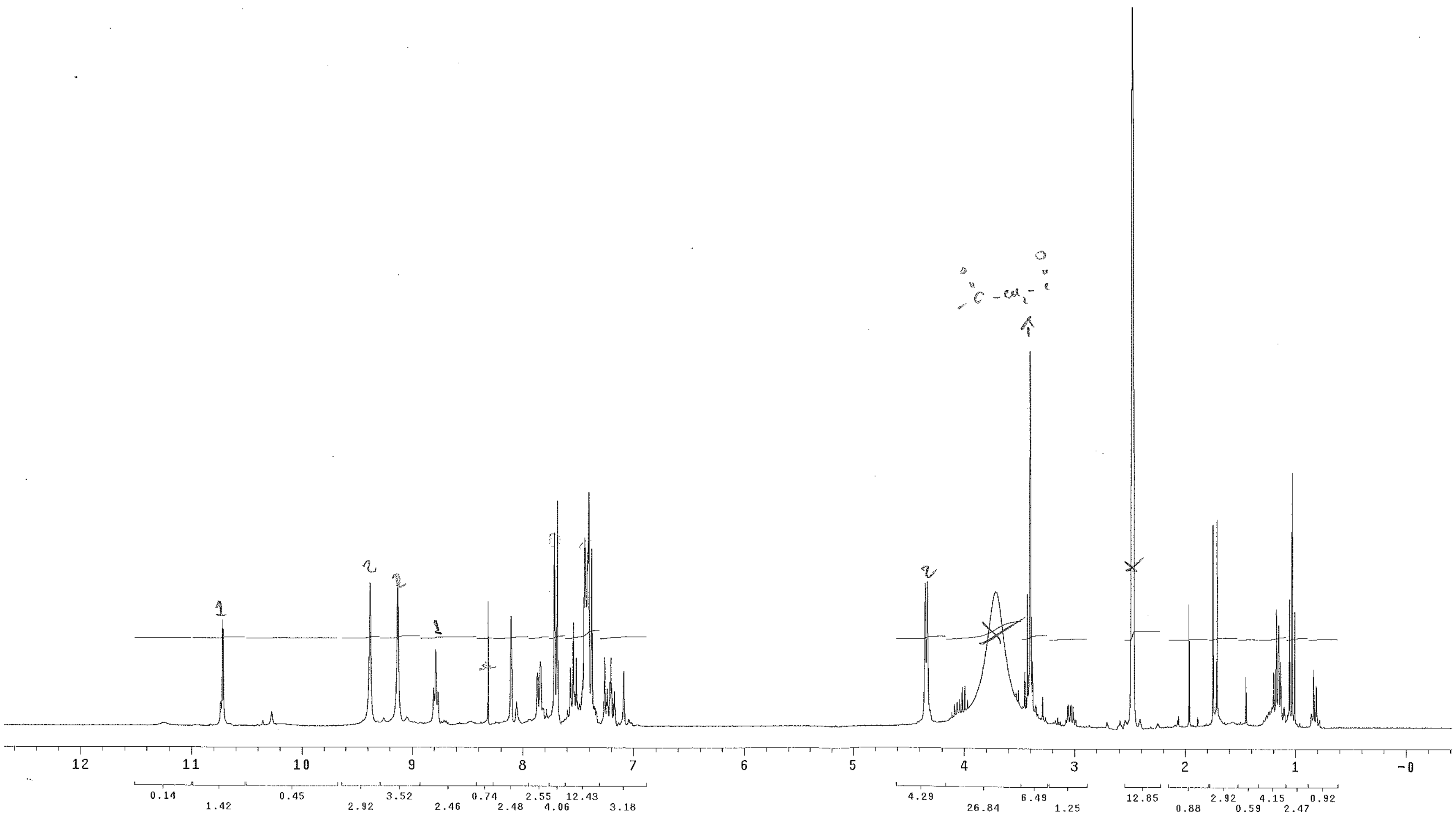
12
11
10
9
8
7
6
5
4
3
2
1
-0
0.14
1.42
0.45
2.92
3.52
2.46
0.74
2.48
2.55
4.06
12.43
3.18
4.29
26.84
6.49
1.25
12.85
0.88
2.92
0.59
4.15
2.47
0.92

19h

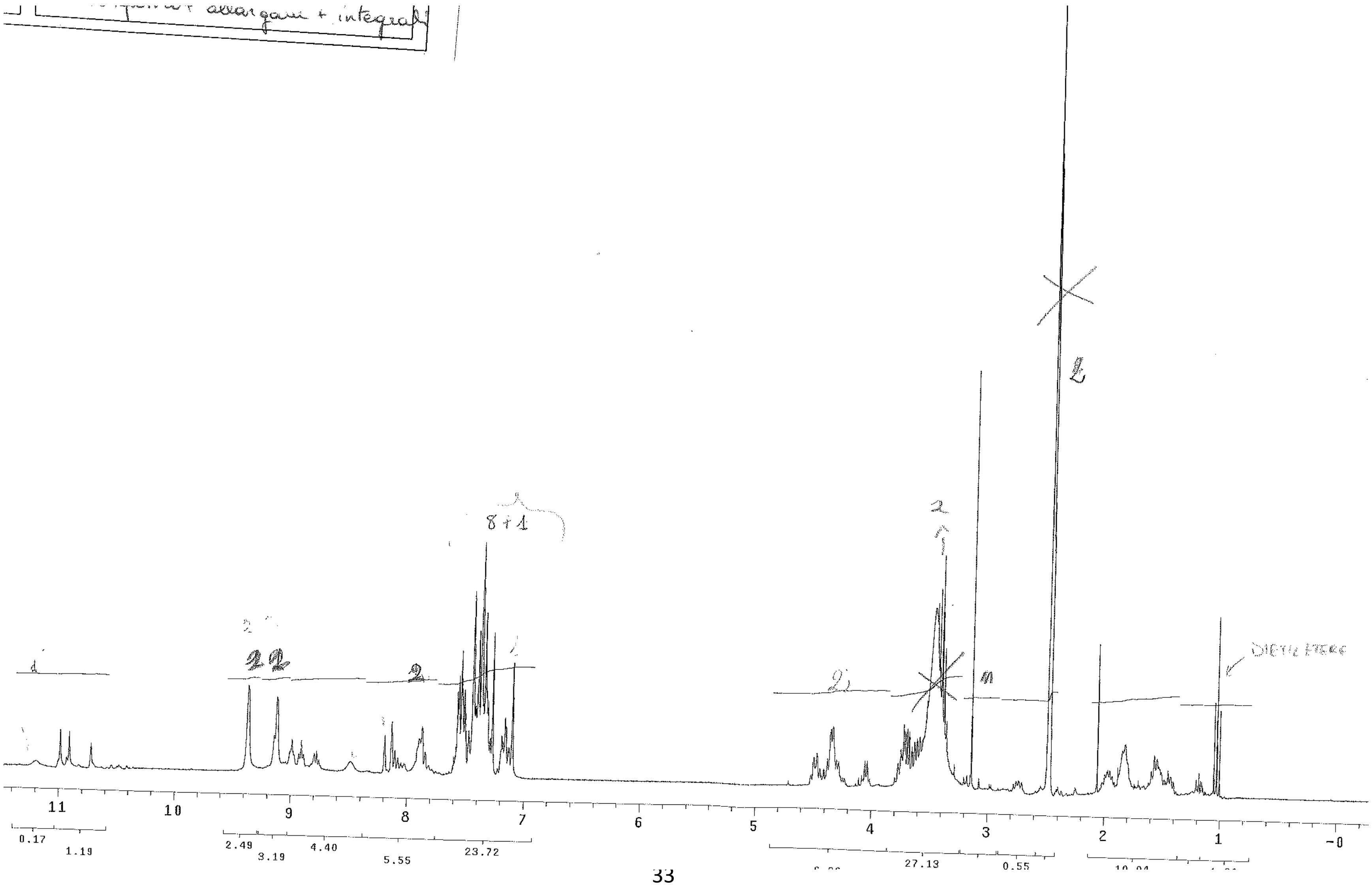

19l

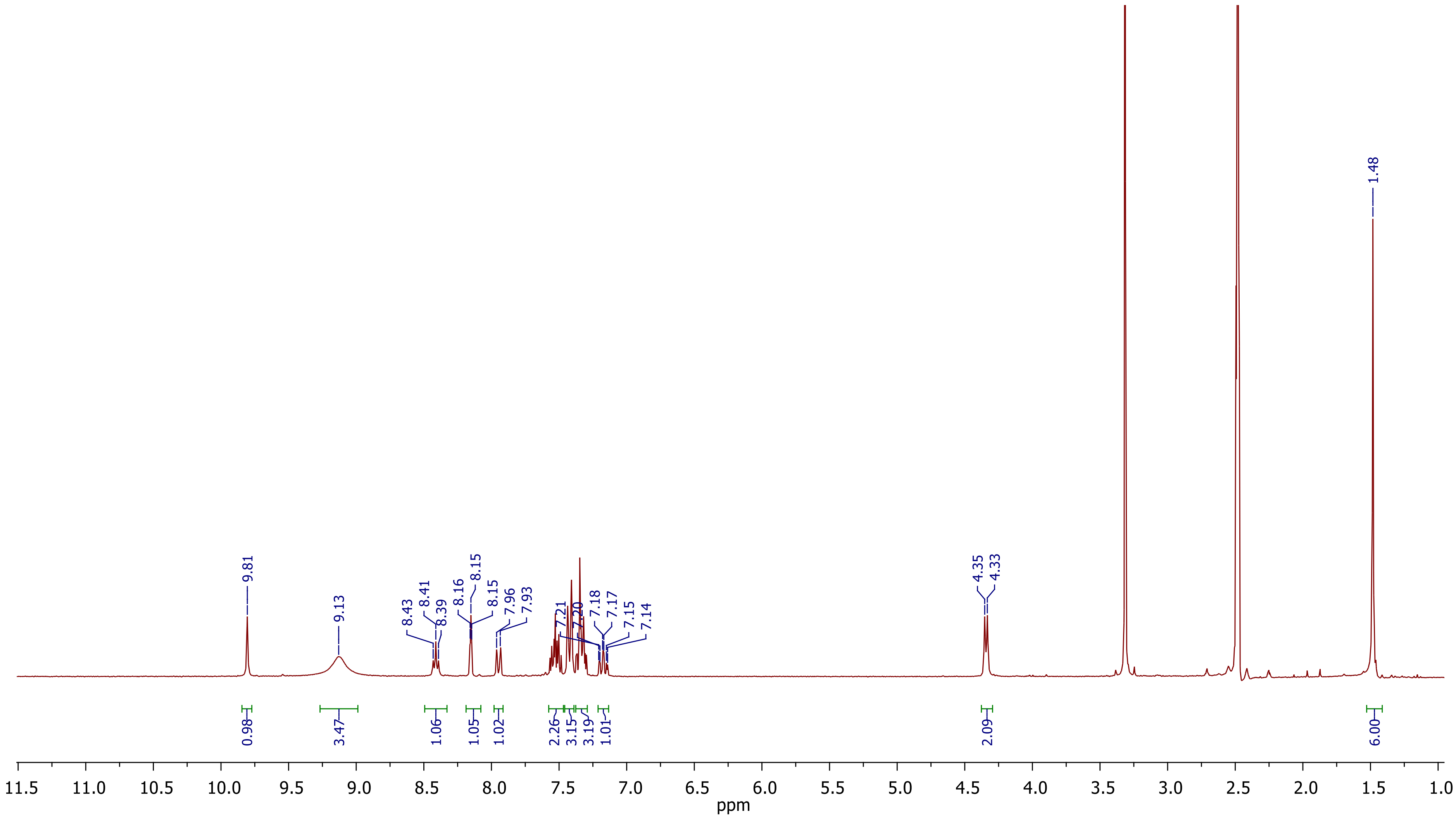
9.81
9.13
8.43
8.41
8.39
8.16
8.15
8.15
7.96
7.93
7.21
7.20
7.18
7.17
7.15
7.14
4.35
4.33
1.48
0.98
3.47
1.06
1.05
1.02
2.26
3.15
3.19
1.01
2.09
6.00
11.5
11.0
10.5
10.0
9.5
9.0
8.5
8.0
7.5
7.0
6.5
6.0
5.5
5.0
4.5
4.0
3.5
3.0
2.5
2.0
1.5
1.0
ppm

20a

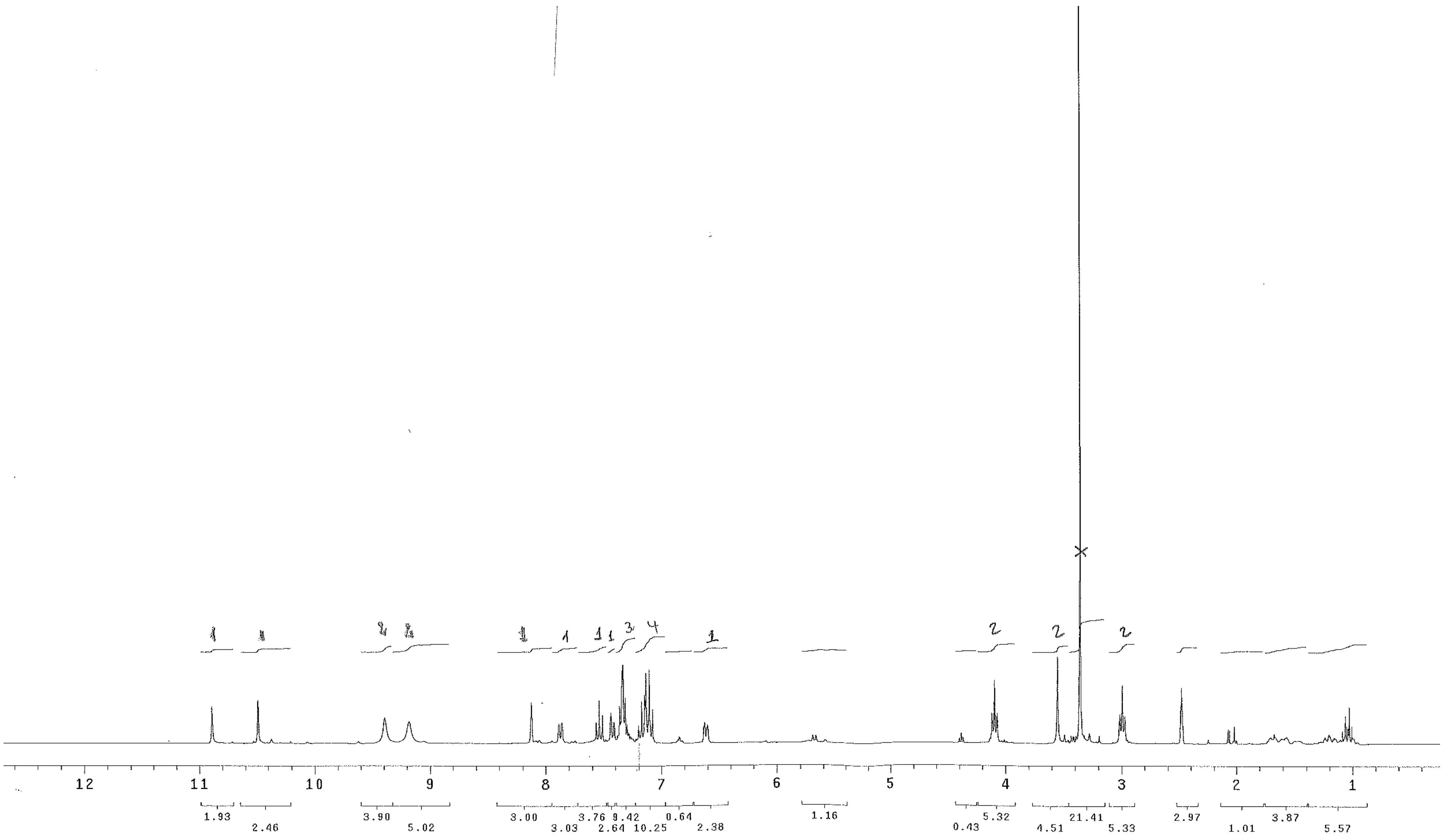

12
11
10
9
8
7
6
5
4
3
2
1
1.93
2.46
3.90
5.02
3.00
3.03
3.76
2.64
9.42
10.25
0.64
2.38
1.16
0.43
5.32
4.51
21.41
5.33
2.97
1.01
3.87
5.57

20b

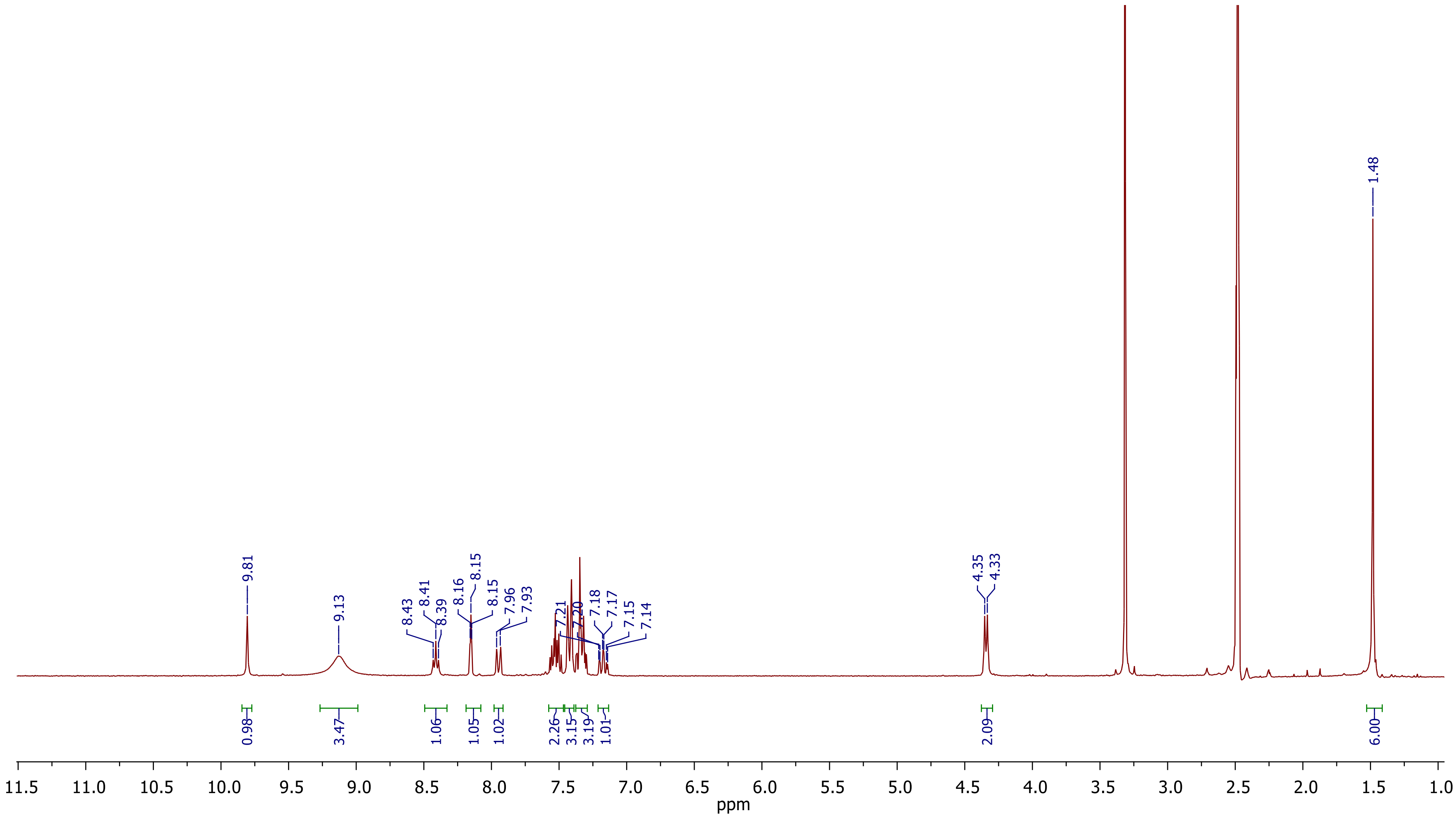
9.81
9.13
8.43
8.41
8.39
8.16
8.15
8.15
7.96
7.93
7.21
7.20
7.18
7.17
7.15
7.14
4.35
4.33
1.48
0.98
3.47
1.06
1.05
1.02
2.26
3.15
3.19
1.01
2.09
6.00
11.5
11.0
10.5
10.0
9.5
9.0
8.5
8.0
7.5
7.0
6.5
6.0
5.5
5.0
4.5
4.0
3.5
3.0
2.5
2.0
1.5
1.0
ppm

21a

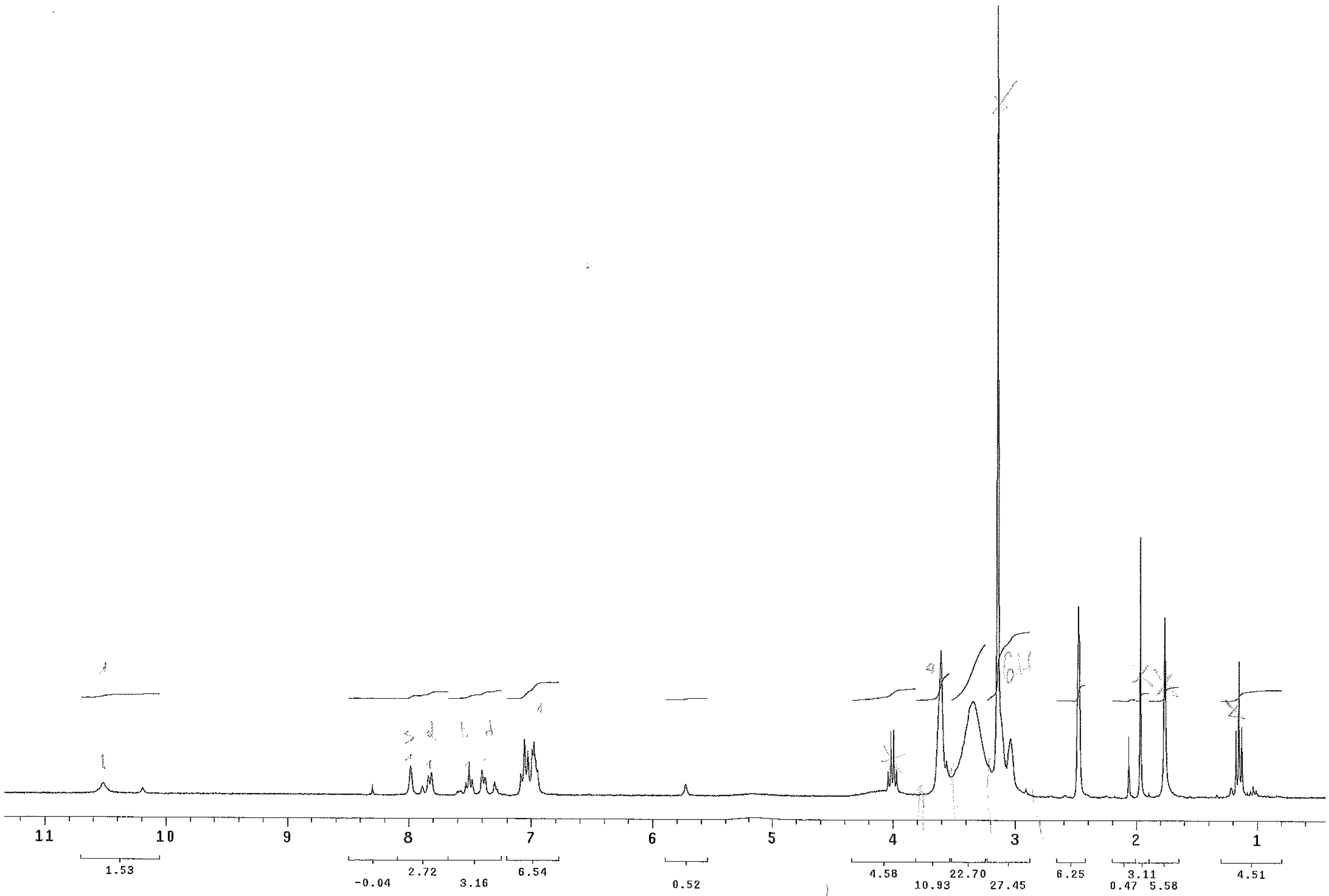

11
10
9
8
7
6
5
4
3
2
1
1.53
-0.04
2.72
3.16
6.54
0.52
4.58
10.93
22.70
27.45
6.25
3.11
0.47
5.58
4.51

22a

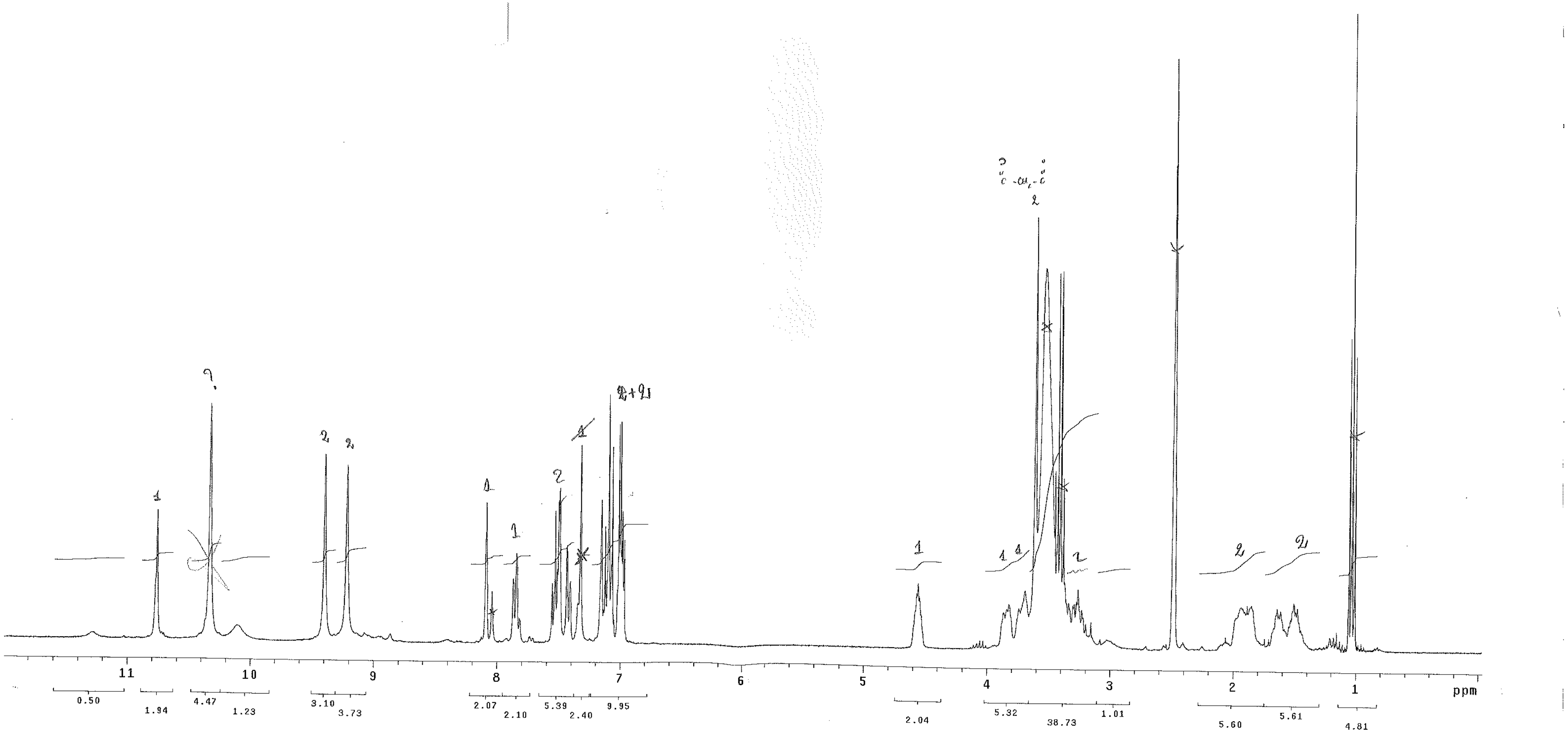

11
10
9
8
7
6
5
4
3
2
1
ppm
0.50
1.94
4.47
1.23
3.10
3.73
2.07
2.10
5.39
2.40
9.95
2.04
5.32
38.73
1.01
5.60
5.61
4.81

22b

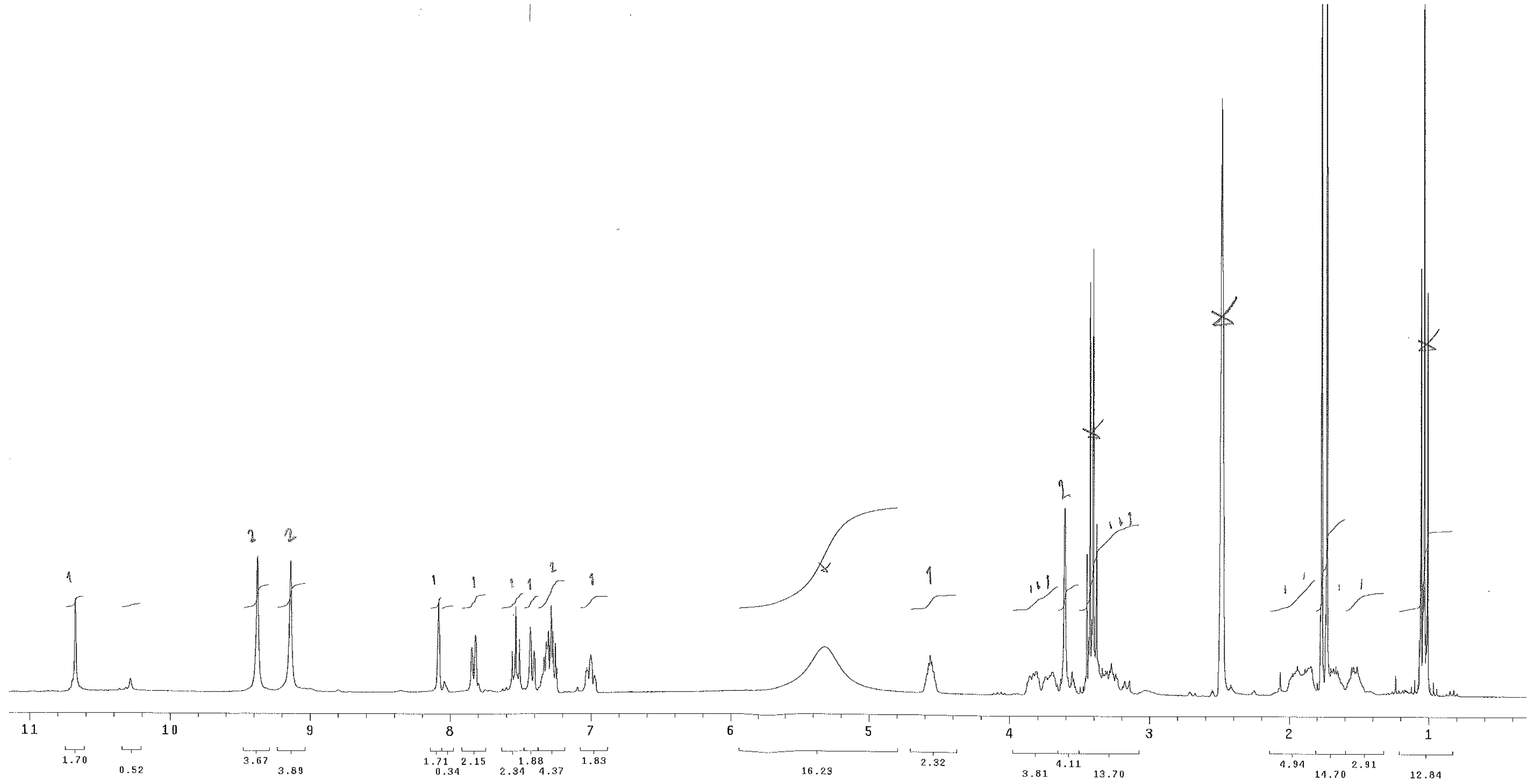
11 10 9 8 7 6 5 4 3 2 1
1.70
0.52
3.67
3.89
1.71
0.34
2.15
2.34
1.88
4.37
1.83
16.23
2.32
3.81
4.11
13.70
4.94
14.70
2.91
12.84

22c

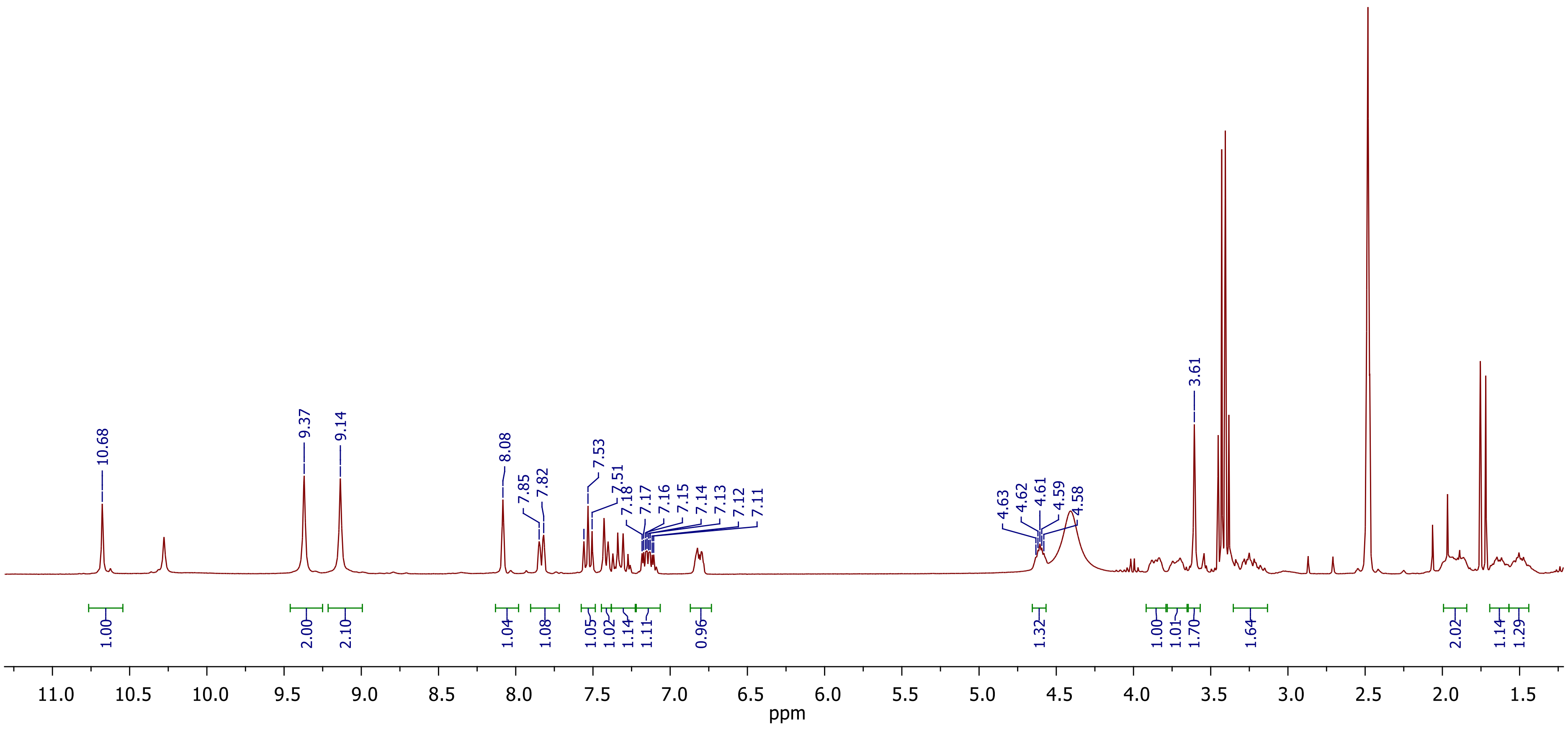
10.68
9.37
9.14
8.08
7.85
7.82
7.53
7.51
7.18
7.17
7.16
7.15
7.14
7.13
7.12
7.11
4.63
4.62
4.61
4.59
4.58
3.61
1.00
2.00
2.10
1.04
1.08
1.05
1.02
1.14
1.11
0.96
1.32
1.00
1.01
1.70
1.64
2.02
1.14
1.29
11.0 10.5 10.0 9.5 9.0 8.5 8.0 7.5 7.0 6.5 6.0 5.5 5.0 4.5 4.0 3.5 3.0 2.5 2.0 1.5
ppm

23a

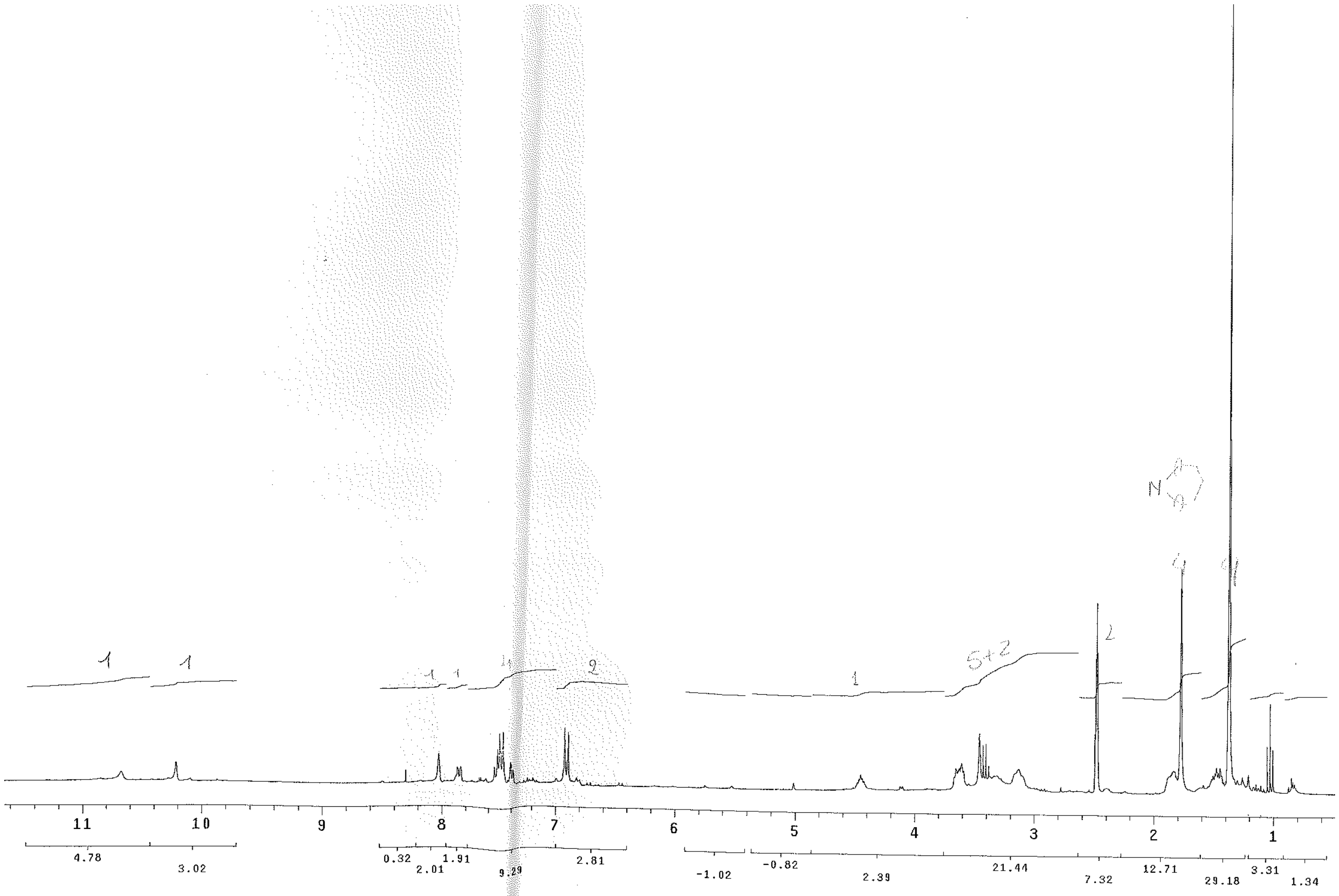

11
10
9
8
7
6
5
4
3
2
1
4.78
3.02
0.32
2.01
1.91
9.29
2.81
-1.02
-0.82
2.39
21.44
7.32
12.71
29.18
3.31
1.34

23b

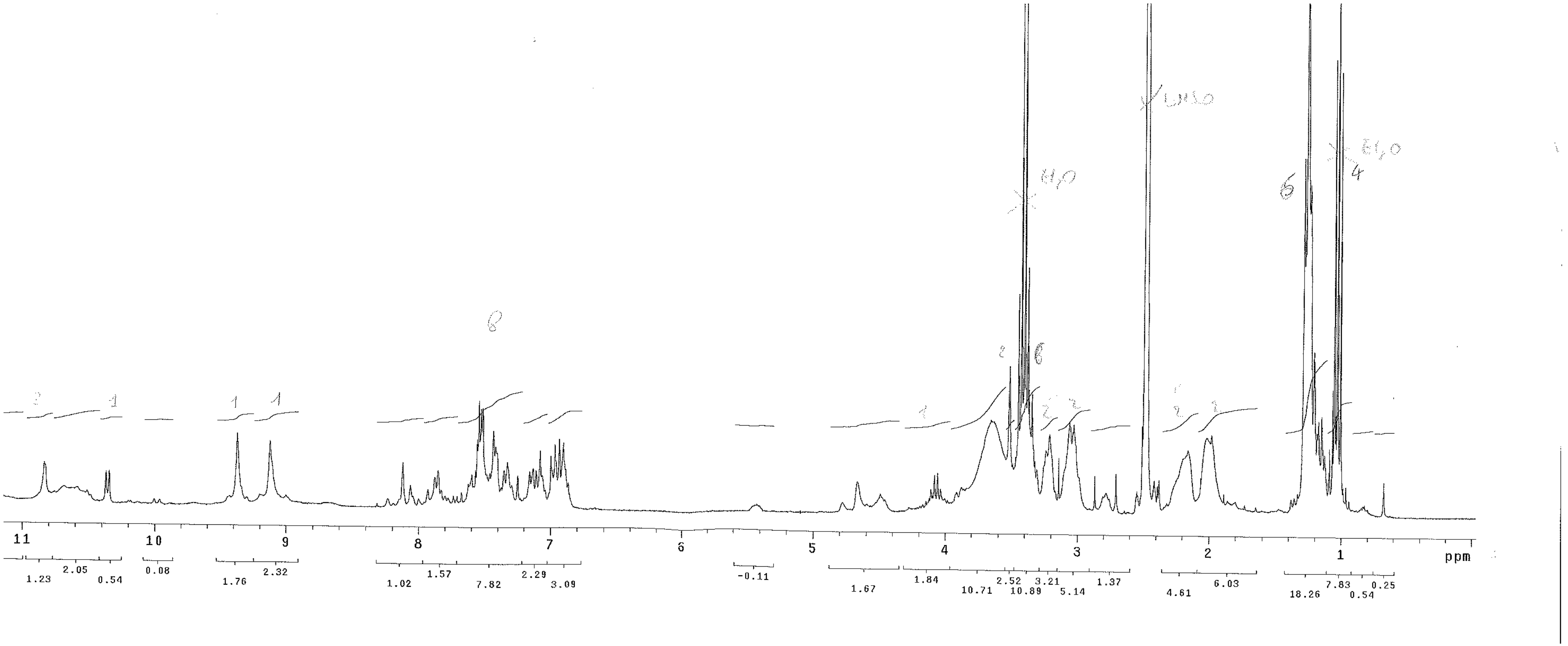

Supplement: Supplementary file 1 [file molecules-27-04269-s001.zip › molecules-1783806-supplementary.pdf]
